# Productivity gains and work conditions in coercive labor markets: Experimental evidence from the Bangladesh brick sector

**DOI:** 10.1073/pnas.2528388123

**Published:** 2026-07-16

**Authors:** Grant Miller, Debashish Biswas, Aprajit Mahajan, Kimberly Singer Babiarz, Nina Brooks, Jessie Brunner, Sania Ashraf, Jack Shane, Alvise Scarabosio, Sameer Maithel, Shoeb Ahmed, Moogdho Mahzab, Mohammad Rofi Uddin, Mahbubur Rahman, Stephen P. Luby

**Affiliations:** ^a^ Department of Health Policy, School of Medicine, Stanford University, Stanford, CA 94305; ^b^ National Bureau of Economic Research (NBER), Cambridge, MA 02138; ^c^ Abdul Latif Jameel Poverty Action Lab (J-PAL), Massachusetts Institute of Technology, Cambridge, MA 02142; ^d^ Health Systems and Population Studies Division, The International Centre for Diarrheal Disease Research, Bangladesh (ICDDR,B), Dhaka 1212, Bangladesh; ^e^ School of Population and Global Health, The University of Western Australia, Perth, WA 6009, Australia; ^f^ Department of Agricultural and Resource Economics, University of California, Berkeley, CA 94720; ^g^ School for Environment and Sustainability, University of Michigan, Ann Arbor, MI 48190; ^h^ Center for Human Rights and International Justice, Stanford Global Studies Division, School of Humanities and Sciences, Stanford University, Stanford, CA 94305; ^i^ Department of Economics, Stanford University, Stanford, CA 94305; ^j^ Stanford Institute for Economic Policy Research, Stanford University, Stanford, CA 94305; ^k^Greentech Knowledge Solutions, New Delhi, 110078, India; ^l^ Department of Chemical Engineering, Bangladesh University of Engineering and Technology (BUET), Dhaka 1000, Bangladesh; ^m^ Poverty, Gender, and Inclusion Unit, International Food Policy Research Institute (IFPRI), Washington, DC 20005; ^n^ Global Health and Migration Unit, Department of Women’s and Children’s Health, Uppsala University, Uppsala SE-75185, Sweden; ^o^ Division of Infectious Diseases and Geographic Medicine, Stanford University, Stanford, CA 94305

**Keywords:** human trafficking, child labor, coercive labor, productivity, informal industry

## Abstract

Productivity growth in competitive labor markets can improve worker welfare over time through higher wages or better conditions, but it is unclear if productivity gains benefit workers in coercive labor markets (where force or threats shape employment). We examine this issue in the Bangladesh brick sector using a randomized trial that introduced a more efficient production method. Despite large productivity improvements, we find no reduction in (high) rates of labor trafficking or child labor. These findings suggest that in the short-term, productivity growth alone may be insufficient to improve work conditions in coercive settings.

## Significance

Standard economic theory predicts that productivity growth should benefit workers through higher wages and/or better conditions in competitive labor markets. We test if this holds in coercive labor markets–characterized by forced labor and threats–using a randomized experiment that introduced a more efficient technology across hundreds of brick kilns in Bangladesh. Despite significant productivity gains, we find no improvements in work conditions or reductions in coercive practices. These findings suggest that coercive conditions can allow firms to fully capture the benefits from improved productivity–and that at least in the short-term, productivity growth alone may be insufficient to improve worker welfare in coercive settings.

Productivity growth is central to theories of economic development (1–5). In these accounts, higher productivity improves worker welfare, in part through improvements in wages and other aspects of work. Such reasoning is compelling in settings with competitive labor markets–firms face incentives to share gains with workers through higher wages or better conditions. However, improvements in productivity may not improve worker welfare in noncompetitive labor markets, such as those with coercive labor practices.

Coercive labor (including labor trafficking (*SI Appendix*, Note 1) and forced labor) as well as child labor are prevalent world-wide, particularly in lower-income countries. Obtaining accurate numbers of these often hidden phenomena is difficult, but at least 27 million people around the world are thought to be victims of forced labor (6, 7), and more than 137 million children are engaged in child labor, with 54 million of them working under hazardous conditions (8). A better understanding of whether or not productivity gains improve work conditions in coercive labor markets is critical for public policy in environments with limited state capacity to enforce labor regulations. However, theoretical models are ambiguous—productivity improvements could decrease or increase coercive labor, underscoring an important role for empirical analyses (9–11) (*SI Appendix*, Note 2). Moreover, empirical studies of the effects of productivity improvements on coercive labor are rare and based on observational, typically historical, analyses (10, 12, 13) (*SI Appendix*, Note 3).

In this project, we experimentally test if a productivity-enhancing technology affects short-term work conditions in a setting with high rates of coercive labor and child labor—brick kilns in Bangladesh. Coercive labor is pervasive in global brick production (14) and has been documented extensively in Bangladesh (*SI Appendix*, Note 4).

More generally, work at brick kilns across South Asia is often characterized by debt bondage, excessive work hours (12 to 16 h work days), and hazardous or degrading work conditions (including lack of personal protective equipment (PPE) and exposure to toxic chemicals) (15). Many workers are seasonal migrants, often bringing their families with them to kilns, leading to substantial child labor as well (16–19) (*SI Appendix*, Note 5).

Our specific context is a large-scale effort to introduce a more productive and energy efficient brick production method across six districts in Bangladesh (20). Through relatively modest changes to coal feeding and brick stacking practices, the improved method reduces spending on coal and increases quality-adjusted output (by increasing the fraction of high-quality bricks, which command a higher price) (20). Building on this work originally designed to study productivity improvements and emissions, we extended our experiment to also study if this more productive manufacturing method influences coercive labor and child labor practices. The trial had three arms: 1) a measurement-only control group (in which we only collected data), 2) a technical intervention group, and 3) a technical+incentive information group. The technical intervention focused on improving productivity, providing information, training, and technical support to kiln owners (along with their managers and workers) to adopt a package of improved operational practices. Because these changes to kiln operations also required important changes in worker routines, the technical+incentive information arm also provided explicit information to kiln owners about positively incentivizing workers for better adoption of these operational improvements—information which could also improve work conditions independent of any productivity gains.

We study the short-term impact of these interventions on both adult labor trafficking and child labor (as well as work conditions generally) through privately conducted surveys with kiln workers.^*^ We measure work conditions and labor trafficking indicators based on U.S. Department of State Office to Monitor and Combat Trafficking in Persons (TIP Office) criteria, several of which were explicitly addressed by the incentive information (21–23). Importantly, these also include outcomes which can change within a single brick-firing season.^†^ Although we did not interview children directly, we collected information about child labor as well from adult workers at each kiln (*SI Appendix*, Note 6).

Despite significant productivity gains, we do not find corresponding short-term improvements in work conditions (including the prevalence of labor trafficking and child labor as well as other measures of welfare like wages and health). Taken together, our results suggest that in coercive labor markets, productivity growth alone may be insufficient to improve worker welfare, at least in the short-run.

## 1. Context, Data, and Methods

### 1.1. Context.

Bangladesh is the fourth-largest producer of bricks globally, with high demand for fired clay bricks driven by rapid urbanization and infrastructure investment (24). Almost 8,000 traditional informal “zigzag” brick kilns operate across the country, supplying more than 90% of domestic consumption (25). We conducted our study in Bangladesh’s Khulna Division, which contains about 700 zigzag kilns operating across 10 districts (according to government data) and 532 zigzag kilns in the 6 districts where we worked. The Bangladesh Brick Manufacturers Owners Association (BBMOA) provided us with a list of 410 kilns from these districts, implying that the 246 kilns in our study (Section 1.2) account for nearly half of all zigzag kilns in study districts. Kilns generally draw workers from nearby–in our data, about 50% of workers were recruited from the surrounding 10 km, and more than two-thirds were recruited from the surrounding 50 km (*SI Appendix*, Fig. A2).

Work at brick kilns in Bangladesh and across South Asia is characterized by debt bondage, excessive hours, and hazardous conditions (15). Labor arrangements are facilitated by the sardar system, with labor recruiters (sardars) also acting as on-site supervisors and intermediaries between kiln owners and workers. Sardars often recruit workers seasonally from their same home communities and use advance payments and informal enforcement to create debt bondage and limit worker mobility (26–28). These arrangements severely constrain workers’ ability to seek alternative employment or negotiate better terms once a brick production season begins. The ability of kilns to replace workers mid-season varies by worker type and specialization (*SI Appendix*, section A5 for more detail on worker roles).

Although public perceptions of human trafficking (including labor trafficking) are often shaped by media depictions of its most egregious forms, potentially creating the impression that human trafficking only occurs in instances of physical violence and restraint, a wider range of conditions meet the formal definition of trafficking (21). Qualitatively, as we found in our initial planning work, typical cases of labor trafficking in our context involve workers taking advance payments from sardars to secure employment for a season. Once onsite, workers find conditions harsh (sometimes harsher than expected or different than disclosed in advance), wages withheld to repay debt from advances, and freedom to leave worksites constrained by credible threats of retaliation (including against workers’ families). Workers are effectively trapped by debt bondage, and in more extreme circumstances, physical restraint also occurs. Our measures of labor trafficking are designed to capture the entire spectrum of coercive conditions.

### 1.2. Experimental Design.

We conducted our study in six districts of the Khulna Division of Bangladesh (Chuadanga, Jashore, Jhenaidah, Khulna, Kushtia, and Narail Districts—see *SI Appendix*, Fig. A1). To identify kilns for inclusion, we first contacted the Brick Manufacturing Owners Association in each district, compiling a list of 410 zigzag kilns (ZZK) (*SI Appendix*, Note 7) from which we aimed to enroll 300 kilns in the trial (based on power calculations and logistical considerations). We collected baseline data from an initial sample of 328 kilns. Due to high coal prices in 2022, some kilns in our initial sample switched to exclusive firewood use, a fuel source for which our technical intervention is not suitable, and some kilns also did not operate during that season. We therefore also enrolled an additional 29 kilns in Jashore District, resulting in a total initial sample of 357 kilns.

We randomly assigned study kilns to experimental arms, stratifying assignment both by district and by quality of bricks—a summary proxy measure of kiln productivity—produced during the previous season (above or below median share of the highest quality (“class 1”) bricks) (*SI Appendix*, Note 8). Using this approach, we generated 1,000 random allocation sets, and for actual treatment assignment, we chose the allocation that maximized the sum of the *P*-values of the *t* tests for kiln characteristics and for which none of the individual *t* tests between arms was statistically significant at the 5% level (29).^‡^ After randomization, we found that 63 kilns were ineligible for treatment (due either to exclusive firewood combustion or nonoperation during 2022). Some kilns were also no longer operating or did not participate in the survey when endline surveys were collected, yielding a final sample of 246 kilns.^§^ Our final sample was powered to detect changes of 18% with 80% power (at the 5% significance level) in counts of labor trafficking indicators. *SI Appendix*, Table A2 shows evidence of balance on observable baseline kiln characteristics in this final study sample, consistent with attrition uncorrelated with treatment assignment.

Our three randomly assigned study arms include: 1) a measurement-only control group, 2) a technical intervention group focused on improving kiln productivity, and 3) a technical+incentive information group receiving both the technical intervention and information about positive worker incentives to encourage adoption of the technical intervention.

Kilns assigned to the technical intervention arm received information, training, and technical support for making technical and operational improvements to their ZZKs (*SI Appendix*, Note 9). The technical intervention also highlighted the financial benefits of these improvements, directly addressing kiln owners’ uncertainty about economic returns. Because the intervention decreased coal use and costs, and increased brick quality and estimated revenue (20), we explicitly test if these improvements in productivity led to improved work conditions (a form of worker compensation, broadly defined).

Because the technical intervention required changes in worker routines, the technical+incentive information arm also provided guidance to kiln owners on using positive incentives to encourage adoption (*SI Appendix*, Note 10). We shared strategies to motivate workers including financial incentives (wages, bonuses, return bonuses) and nonfinancial amenities (better work conditions, improved housing, protective equipment, school facilities for children), see *SI Appendix*, section A4 for details (*SI Appendix*, Note 11). These examples were directly informed by the experience of other kiln owners successfully operating ZZKs, our own pilot study in Jashore district (30), and the management literature (31)—including evidence from brick kilns in Nepal (32) and garment factories in Bangladesh (33) (*SI Appendix*, Note 12). We conducted two follow-up visits with technical+incentive information arm kilns to reinforce this information. This intervention tests if, in addition to the technical intervention and any gains in kiln productivity, providing information about the ability of positive worker incentives to increase worker productivity (and hence profitability) leads owners to improve work conditions.

*SI Appendix*, Fig. A4 shows the timeline of our study activities during the 2022–2023 brick production season.

This project was reviewed and approved by Institutional Review Boards (IRBs) at Stanford University (#67263) and ICDDR,B (PR-22052). A preanalysis plan was submitted to AEA (#0010127) and ISCRTN (#15354089) RCT registries and is accessible here.

### 1.3. Data Collection, Measurement, and Balance.

To measure work conditions, we conducted a detailed survey in private with workers at brick kilns between March and May 2023.^¶^ In accordance with local IRB guidelines, research staff first secured verbal permission from kiln owners, managers, and sardars before approaching brick workers at kilns for participation. Eligible workers were then informed about the purpose of the survey, what participation involved, the voluntary nature of participation, and their right to decline or withdraw at any time. Consent was obtained before survey administration, using approved consent forms available in Bangla. At each study kiln, we interviewed 6 individuals (5 workers and 1 sardar, or recruiter and on-site supervisor who act as an intermediary between kiln owners and workers), focusing on four types of workers: brick molders (who shape clay to form “green” bricks before they are fired), brick loaders (who load “green” bricks into kilns), brick unloaders (who remove fired bricks from kilns), and firemen (who feed coal into kilns to bake bricks).^#^ Through these surveys, we collected detailed information about wages, work conditions, migration status, occupational hazards, and the age range of workers at kilns.

**Fig. 1.**
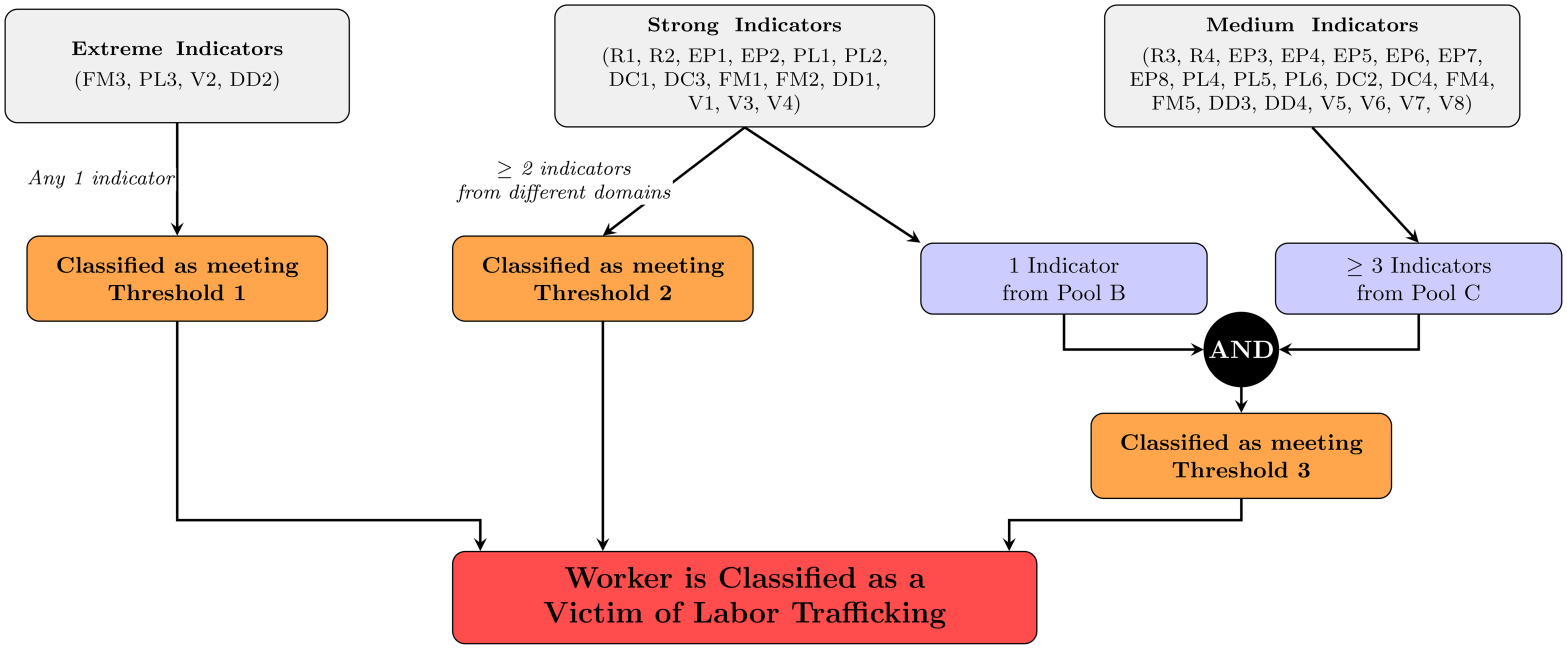
Labor trafficking classification flowchart. *Notes:* This flowchart illustrates the three thresholds used to classify a worker as a victim of labor trafficking, following the framework of Okech et al. (23). This framework encompasses all forms of human trafficking, and we list all indicators included in Okech et al. (23) for completeness. However, this paper only measured, and studies, labor trafficking (not sex trafficking and other forms of human trafficking)—see *SI Appendix*, Table A1 for the subset of indicators collected. A worker is classified if they meet the criteria for Threshold 1, Threshold 2, OR Threshold 3. The codes (e.g., FM3, R1) correspond to specific trafficking indicators as listed in *SI Appendix*, section 1.

Our survey instrument also included a multi-indicator human trafficking measurement tool developed by the U.S. Department of State through its Prevalence Reduction Innovation Forum (PRIF) (22, 23).^‖^ This measurement tool consists of 39 indicators of human trafficking across seven domains (recruitment, employment practices, control over personal life and property, degrading conditions, freedom of movement, debt and dependency, and violence, see *SI Appendix*, Table A1). Following Okech et al. (23), we classify indicators by strength as either “strong” (14 indicators) or “medium” (21 indicators). Additionally, we refer to 4 other strong indicators in Okech et al. (23) as “extreme” because, unlike the others, each of these individually constitutes trafficking (*SI Appendix*, Note 13). As depicted in Fig. 1, a worker is determined to be trafficked if the combination of indicators they experience meets one of three thresholds: 1) Threshold 1 is met if any one of four extreme indicators are experienced (no freedom of movement/communication, hereditary bonded labor, being sold for labor or sex, or being made to work in commercial sex); 2) Threshold 2 is met if two or more strong indicators from two different domains are experienced (e.g., coercive or deceptive recruitment, confiscation of documents or identification, etc.); and 3) Threshold 3 is met if at least one strong and three medium indicators are experienced (e.g., debt imposed without consent along with the absence of a formal contract, wages withheld and not guaranteed, and constant surveillance at work). Throughout the rest of the paper, we define “prevalence” as the occurrence of labor trafficking using this indicator-based definition. Although trafficking indicators are standard measures established by the TIP Office, subjective judgment is required to measure some indicators. Because we are not aware of guidance for these cases, we present results using a more “conservative” coding, but *SI Appendix* also shows equivalent results using a more “liberal” coding (*SI Appendix*, Note 14). *SI Appendix*, section A1 gives a complete description of our human trafficking classification methodology.

The sensitive nature of the indicators suggests that misreporting could also be a concern.^**^ To the extent that any misreporting is independent of treatment assignment (which is plausible), its impact on our treatment effect estimates depends on the nature of the outcome variable. For a continuously distributed outcome (with unbounded support), our estimates would be consistent and unbiased, but estimated with reduced precision. Alternatively, for a binary dependent variable (with limited misclassification), treatment effects estimated using Ordinary Least Squares (OLS) would be attenuated toward zero (*SI Appendix*, Note 15).

Our analysis has four prespecified primary outcomes. The first outcome is a weighted count of labor trafficking indicators at the kiln level. To account for differences in severity among medium, strong, and extreme indicators (as specified by TIP Office guidelines), we assign medium indicators two-thirds of the weight of strong and extreme indicators (as stated in our preanalysis plan). Second, again at the kiln level, we code a count of the number of individual trafficking indicators that were explicitly linked to the incentive information intervention (there are three in total).^††^ Third, we code prevalence of labor trafficking at the kiln level using combinations of indicators that constitute trafficking according to guidelines established by the TIP Office (22, 23). Fourth, we measure child labor at the kiln level using reports by surveyed adult workers, each of whom was asked about the presence of children working at kilns (and these children’s approximate ages). We consider any child under age 14 who was working and any child age 17 or younger who was working under hazardous conditions (conditions at brick kilns are generally hazardous) to be child labor.^‡‡^ To better distinguish between the presence of these practices at a kiln and the extent (or prevalence) of these practices, we analyze outcomes at both the kiln level (whether or not a practice occurred at a kiln) and the worker level (how many workers interviewed at a kiln reported a practice).

We use October 2022 baseline data collected on kilns to demonstrate balance on observable kiln characteristics (given that we did not conduct a baseline worker survey) (*SI Appendix*, Table A2), and we also demonstrate balance using time-invariant worker characteristics (*SI Appendix*, Table A3). *SI Appendix*, Table A4 presents descriptive statistics from our worker survey.

### 1.4. Estimation.

We estimate Intention-to-Treat (ITT) effects of the interventions on our four primary outcomes. Specifically, we estimate ITT effects for each treatment arm using the following basic framework:

[1] $$\begin{array}{c} Y_{i}=\beta_{0}+\beta_{1}T_{i}+\beta_{2}I_{i}+\gamma_{s}+\epsilon_{i}, \end{array}$$

where $Y_{i}$ is an outcome of interest for kiln (or worker) i, $T_{i}$ is a binary indicator for assignment to the technical intervention arm, $I_{i}$ is a binary indicator for assignment to the technical+incentive information intervention arm, and $\gamma_{s}$ are randomization strata fixed effects. The coefficients for each treatment indicator ($\beta_{1}$ and $\beta_{2}$) capture the ITT effect of assignment to the treatment arms on each of the outcomes relative to the control arm. For worker-level outcomes, we compute heteroskedasticity-robust SEs clustered at the kiln level, and for kiln-level outcomes, we compute heteroskedasticity-robust SEs. If either of our randomized interventions improves the productivity of brick kilns, this ITT estimation framework then allows us to study how they also directly lead to changes in labor conditions as well (*SI Appendix*, Note 16).

## 2. Results

In this section, we present results that analyze the impact of our interventions on coercive labor practices, child labor, and other work conditions. We report detailed estimates of the impact of the technical intervention elsewhere (20) and include key findings on adoption of the technical intervention, fuel use and spending, brick quality, and other input costs in *SI Appendix*, Tables A6 and A7. For context, the technical intervention decreased fuel costs by 9.6% and increased brick quality by 8.1% relative to the control group. These effects did not differ significantly between the two treatment groups and there were no significant changes in kiln costs.

### 2.1. Prevalence of Trafficking Indicators, Labor Trafficking, and Child Labor.

**Fig. 2.**
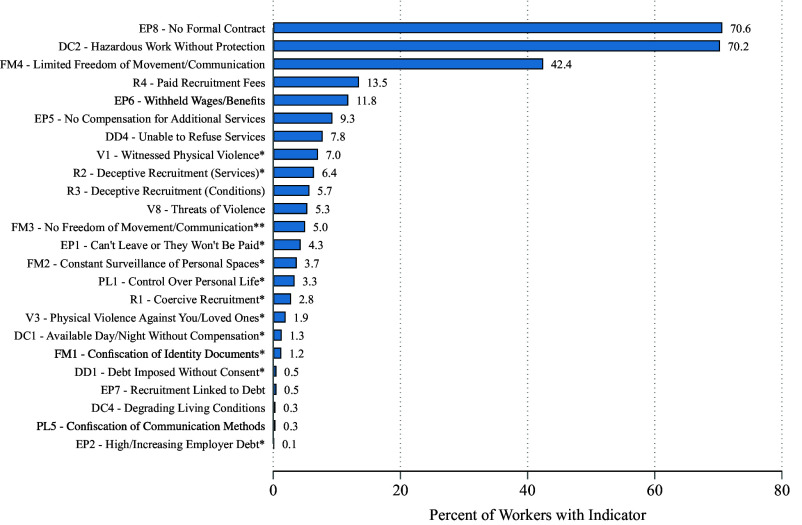
Prevalence of labor trafficking indicators at study kilns. *Notes:* Sample includes data from 246 brick kilns, with a total of 1,442 workers interviewed. All indicators come from Okech et al. (23). Note that not all indicators mentioned in Okech et al. (23) are included here, because our survey does not ask about sex trafficking or sexual violence. See *SI Appendix*, section A1 for more information on trafficking indicators. * = Strong Indicator. ** = Extreme Indicator.

Fig. 2 shows the prevalence of individual trafficking indicators at study kilns. Overwhelmingly, workers report a lack of PPE. A large majority of workers (70%) do so using a conservative coding (and all workers (100%) do so using a liberal coding, as shown in *SI Appendix*, Fig. A5). Lack of PPE at brick kilns commonly leads to burns, head injuries, eye irritation, and smoke inhalation (37–39). 71% of workers also report that they do not have a formal labor contract (*SI Appendix*, Note 17). 42% of workers say that they have limited freedom of movement or communication, conditions often implying inability to leave a worksite voluntarily (and 5% report that they have no freedom of movement or communication). Other trafficking indicators reported less commonly by workers include wages or benefit withholding (11.8%), deceptive or coercive recruitment (6.4% and 2.8%, respectively), constant surveillance of personal space (3.7%), and violence against other workers or people about whom they care (7.0% and 1.9%, respectively). *SI Appendix*, Figs. A6–A8 show the corresponding distributions of trafficking indicators by study arm (*SI Appendix*, Note 18).

**Fig. 3.**
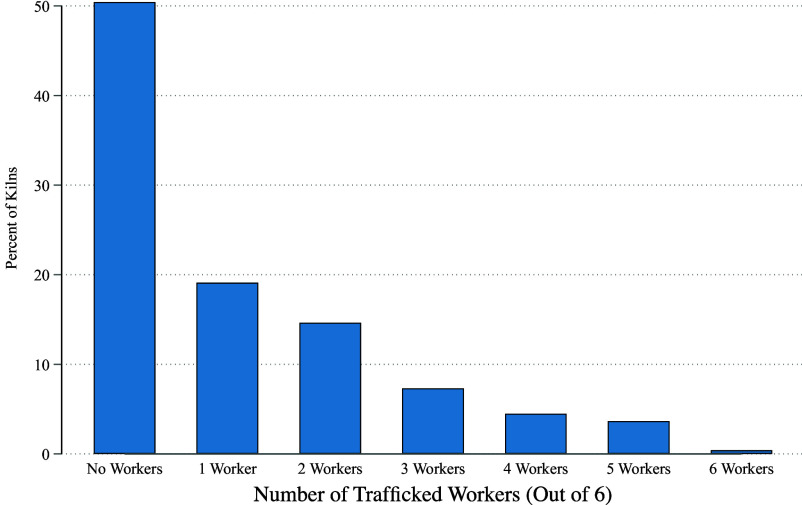
Share of study kilns by number of workers who are trafficked (out of 6). *Notes:* Sample includes data from 246 brick kilns, with a total of 1,442 workers interviewed. Trafficking classifications follow Okech et al. (23). See Table 1 and *SI Appendix*, section A1 and Table A1 for more information on trafficking classification.

We next apply PRIF definitions to work conditions to measure the prevalence of labor trafficking (23). Fig. 3 shows the share of study kilns by number of surveyed workers (out of 6) who were classified as trafficked (and *SI Appendix*, Fig. A9 shows this using both liberal and conservative codings). At half of kilns (49.6%), at least 1 worker out of 6 meets the definition of labor trafficking. Across all surveyed workers, the average number of trafficked workers per kiln (out of 6 workers) is about 1.1 worker per kiln—a trafficking rate of roughly 20%.

**Fig. 4.**
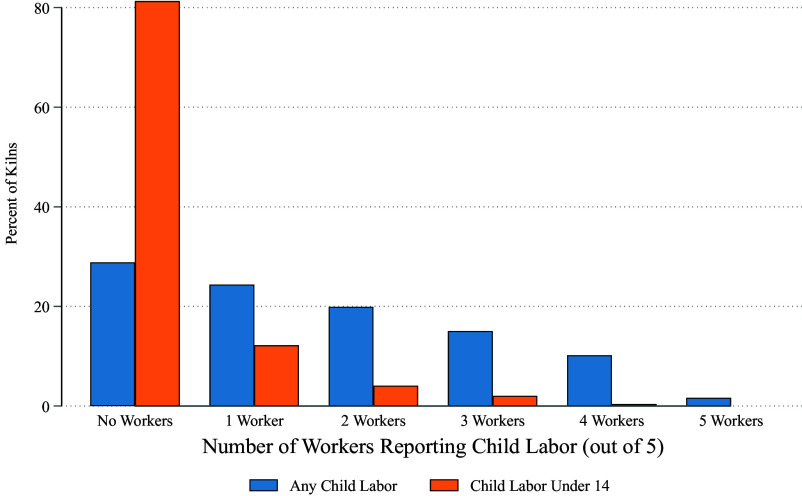
Share of study kilns by number of workers (Out of 5) who report seeing child labor. *Notes:* Sample includes data from 245 brick kilns, with a total of 1,198 workers interviewed. This differs from the Trafficking Indicators sample because sardars were not asked about children working at the kiln. In one kiln, only a sardar was interviewed, so the observation is lost. Respondents are classified as having seen child labor if they either have their own child work at the kiln with them or if they report that at least one member of their team is underage. The blue bars show results for all children (17 y old or younger). The orange bars show results for children under 14. Note that we were unable to directly interview children at study kilns.

Fig. 4 presents results for the prevalence of any child labor (shown in blue), showing that more than 70% of kilns in our sample use child labor according to reports by adult workers. The average number of workers per kiln reporting child labor is about 1.6 (out of 5). We present results for the youngest group of children (under age 14) in orange on Fig. 4. In about 20% of kilns, at least one worker reported seeing children under the age of 14 working, and the average number of workers per kiln reporting child labor under age 14 is about 0.3 (a prevalence rate of about 5%). Taken together, Fig. 4 implies that most of the child labor that we observe is concentrated among children ages 14 to 17.

### 2.2. Experimental ITT Estimates for Labor Trafficking, Child Labor, and Incentivized Work Conditions.

#### 2.2.1. Labor trafficking indicators and prevalence.

Columns 1 to 4 in Table 1 show ITT estimates from Eq. **1** for the effect of the technical and technical+incentive information interventions on the number of all trafficking indicators and also the number of indicators targeted by the incentive information intervention, using conservative codings. We measure these outcomes both at the worker level (the number of indicators each individual worker experiences) and at the kiln level (the number of indicators experienced by at least one surveyed worker at the kiln). See Columns 1 to 4 in *SI Appendix*, Table A5 for results using liberal codings and *SI Appendix*, Fig. A10 for results using a range of alternative weights for the medium indicators. Our results are generally insensitive to these choices. As the table shows, neither intervention significantly reduced the number of trafficking indicators, either at the worker or at the kiln level. We also conducted equivalence tests using a Two One-Sided *t* Tests (TOST) procedure (40), finding that we can rule out reductions in labor trafficking indicator counts of 16 to 17% (P<0.05), or standardized effect sizes of 0.25 to 0.27.

**Table 1.** ITT estimates for labor trafficking by study arm

Treatment arm	Weighted count of indicators				Trafficking prevalence	
	(1)	(2)	(3)	(4)	(5)	(6)
Technical only	0.097	−0.021	0.206	−0.022	0.029	0.071
	(0.131)	(0.026)	(0.341)	(0.054)	(0.037)	(0.078)
Technical + incentive	0.088	−0.010	0.510	0.008	0.005	−0.037
	(0.121)	(0.021)	(0.364)	(0.042)	(0.037)	(0.078)
Control mean	1.858	0.488	3.988	0.703	0.177	0.482
Kiln or worker level?	Worker	Worker	Kiln	Kiln	Worker	Kiln
All or target?	All	Target	All	Target	–	–
Observations	1,442	1,442	246	246	1,442	246

SEs in parentheses. All SEs are clustered at the kiln level for worker level analyses and are heteroskedasticity-robust for kiln level analyses. Columns 1 to 4: The dependent variable is a weighted count of the trafficking indicators observed; medium indicators receive 2/3rds the weight of strong and extreme indicators. Estimates are OLS estimates generated from regressing the weighted count of trafficking indicators on treatment arm dummy variables, with fixed effects for randomization strata. Columns 1 and 2 show regression results for the worker-level weighted count of indicators. Columns 3 and 4 show results for the weighted count of unique indicators in each kiln. Target indicators use the same weighting and include indicators that are most closely related to the incentives: DC1 (Made to be available day and night without adequate compensation outside of the scope of the contract); DC2 (Made to complete hazardous and/or arduous services without proper protective gear); and DC4 (Made to live in degrading conditions). Columns 5 and 6: Estimates are OLS estimates generated from regressing an indicator for trafficking on treatment arm dummy variables, with fixed effects for randomization strata. To generate kiln-level data, we code kilns as having trafficking if any of the workers interviewed at that kiln met the definition for trafficking. * (*P* < 0.1), ** (*P* < 0.05), and *** (*P* < 0.01).

Columns 5 and 6 in Table 1 repeat this analysis for prevalence of labor trafficking. This outcome is also measured at the worker level (whether or not a given individual is classified as trafficked) and at the kiln (whether or not any worker at the kiln is classified as trafficked). We find little evidence of statistically significant reductions, both at the worker and kiln level, using conservative coding (see *SI Appendix*, Table A5 Columns 5 and 6 for the comparable results using liberal coding).

Overall, there is little evidence that our interventions reduced either the number of trafficking indicators that workers experienced or the share of workers meeting the trafficking threshold. ITT estimates for both of our interventions are statistically insignificant for the outcomes that we study, and they are sufficiently precise to rule out socially meaningful effects.^§§^ Although our study focuses on short-run partial equilibrium effects, these results stand in contrast to the common empirical finding in noncoercive labor markets that productivity gains translate into gains in worker welfare through higher wages and better living standards [as implied by standard growth models (2, 3)].

#### 2.2.2. Child labor prevalence.

We also examine how our experimental interventions influenced child labor at brick kilns. Table 2 reports ITT estimates from Eq. **1** for three distinct outcomes. First, we measure its presence at a kiln using a kiln-level indicator for whether or not any surveyed worker reported observing child labor at a given kiln. Second, to measure its prevalence, we use two individual-level outcomes: a binary variable for whether or not a worker personally observed child labor, and a continuous measure reflecting the share of children on that worker’s team.^¶¶^ The first row of the table shows that we find no evidence that the technical intervention alone reduced child labor, either among younger children (under age 14) or all children present at kilns. By contrast, the second row shows some suggestive evidence that the technical+incentive information intervention may have reduced work among all children at kilns. However, after implementing our prespecified multiple hypothesis test adjustments, the resulting adjusted *P*-values (shown in square brackets) are statistically insignificant at conventional levels.^##^ We therefore do not strongly interpret these results to provide clear evidence of a meaningful decline in child labor.

**Table 2.** ITT estimates for child labor by study arm

Treatment arm	Observed child labor		Share of child workers		Any child labor at kiln	
	(1)	(2)	(3)	(4)	(5)	(6)
Technical only	−0.006	−0.030	−0.007	−0.002	0.022	−0.104
	(0.041)	(0.022)	(0.007)	(0.002)	(0.069)	(0.064)
Technical + incentive	−0.073*	−0.027	−0.009	−0.002	−0.079	−0.100
	(0.040)	(0.020)	(0.007)	(0.002)	(0.072)	(0.061)
	[0.346]					
Control mean	0.353	0.078	0.048	0.007	0.735	0.265
Kiln or worker level?	Worker	Worker	Worker	Worker	Kiln	Kiln
All child labor or under 14?	All	Under 14	All	Under 14	All	Under 14
Observations	1,198	1,198	1,198	1,198	245	245

SEs in parentheses and q-values in square brackets. All SEs are clustered at the kiln level for worker level analyses and are heteroskedasticity-robust for kiln level analyses. Q-values are calculated using the Benjamini–Krieger–Yekutieli (42) sharpened two-stage procedure. Estimates are OLS estimates generated from regressing child labor outcomes on treatment arm dummy variables, with fixed effects for randomization strata. Columns 1 and 2: binary indicator for whether a worker personally observed child labor. Columns 3 and 4: the share of workers in the respondent’s team who are children. Columns 5 and 6: kiln-level indicator for whether any surveyed worker reported observing child labor. For all columns, child labor is defined as workers under the age of 18 (columns 1, 3, 5) or under the age of 14 (columns 2, 4, 6). A test of equality between the treatment arm estimates in column 1 has a *P*-value of 0.102, for column 3 the *P*-value is 0.772, and for column 5 the *P*-value is 0.159. Sample includes data from 245 brick kilns, with a total of 1,198 workers interviewed. This differs from the Trafficking Indicators sample because sardars were not asked about children working at the kiln. In one kiln, only a sardar was interviewed, so the observation is lost. * (*P* < 0.1), ** (*P* < 0.05), and *** (*P* < 0.01).

#### 2.2.3. Other work conditions.

**Fig. 5.**
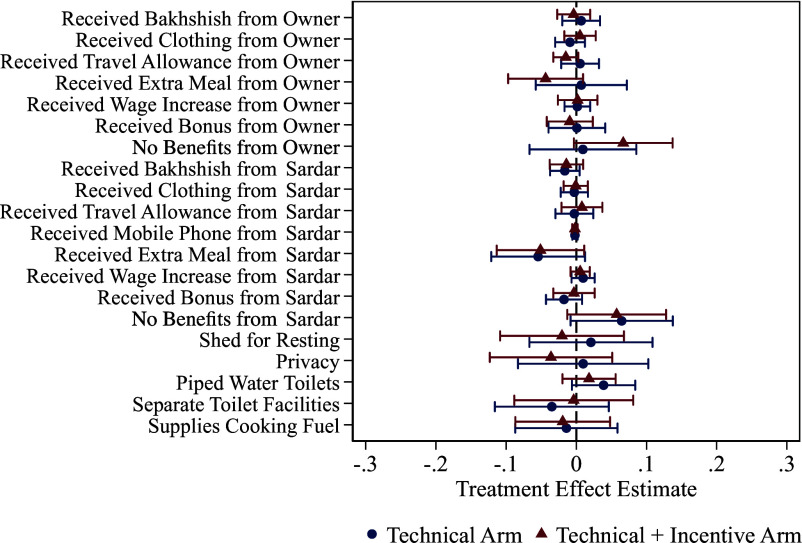
ITT estimates for individual incentivized work conditions. *Notes:* Sample: 246 kilns (1,442 workers) surveyed in both the worker survey as well as kiln performance monitoring. Graphs show the OLS estimates and 95% CIs generated from regressing an indicator for each amenity on treatment arm dummy variables with randomization strata fixed effects. Bahkshish is a term often used in South Asia to describe small informal payments made to individuals (similar to a tip), while bonuses are typically more formal and given at the conclusion of a service. Bonuses often are predetermined, while bahkshish amounts may be declared at the time of giving.

Although not prespecified, we also study how work conditions more broadly (beyond labor trafficking and child labor) may have changed in response to productivity gains. Focusing on specific worker amenities explicitly highlighted as part of the technical+incentive information arm script (*SI Appendix*, section A4), Fig. 5 shows ITT estimates for individual amenities from Eq. **1**. In general, these estimates are statistically indistinguishable from zero, and they are also similar in both the technical and technical+incentive information groups as well. Overall, they suggest little improvement across a wide range of amenities, consistent with what owners also reported (20).

Table 3 shows estimates for worker wages and health outcomes. It suggests that wages paid to kiln workers did not rise despite an increase in the return to worker effort (an implied increase in the marginal revenue product of labor). It also reports results for respiratory health, workplace injuries, and mental health conditions (anxiety and depression, measured using clinically validated GAD-7 and PHQ-9 questionnaires), showing that although respiratory symptoms, workplace injuries, anxiety, and depression are highly prevalent, none declined significantly as a result of our interventions (*SI Appendix*, Note 19).

**Table 3.** ITT estimates for worker wages and health outcomes

	Wages	Respiratory health					Other physical health		Mental health	
	Weekly wages	Any symptoms	Respiratory problems	Constant cough	Difficulty breathing	Wheezing/asthma	Work injury	Any physical symptoms	Anxiety (GAD-7)	Depression (PHQ-9)
Technical only	−29.47	0.032	−0.000	0.038**	−0.004	−0.012	−0.010	0.038	0.093	0.013
	(88.68)	(0.022)	(0.007)	(0.017)	(0.016)	(0.012)	(0.035)	(0.030)	(0.266)	(0.262)
				[0.179]						
Technical + incentive	−27.17	−0.006	−0.001	0.006	−0.013	−0.007	0.011	0.017	−0.038	−0.104
	(85.59)	(0.021)	(0.007)	(0.014)	(0.014)	(0.012)	(0.033)	(0.029)	(0.250)	(0.256)
Control mean	3,811.08	0.112	0.014	0.045	0.053	0.043	0.293	0.230	4.350	4.688
Observations	1,198	1,442	1,442	1,442	1,442	1,442	1,442	1,442	1,442	1,442

SEs in parentheses, clustered at the kiln level. Q-values shown in square brackets are calculated using the Benjamini–Krieger–Yekutieli (42) sharpened two-stage procedure. Estimates are OLS estimates from regressions of each outcome on treatment arm dummy variables, with fixed effects for randomization strata. Weekly Wages are measured in Bangladeshi Taka (BDT). Any Respiratory Symptoms equals 1 if the respondent reported any respiratory issue (either work-related respiratory problems or any of the three respiratory symptoms). Respiratory Problems equals 1 if respiratory problems were reported in the work injury module. Constant Cough, Difficulty Breathing, and Wheezing/Asthma are indicators for specific respiratory symptoms reported in the physical health module. Work Injury equals 1 if any work-related injury was reported. Any Physical Symptoms equals 1 if any physical health symptom was reported. Anxiety is an integer score (0 to 21) based on the GAD-7 anxiety scale. Depression is an integer score (0 to 27) based on the PHQ-9 depression scale. The sample in the first column includes data from 245 brick kilns, with a total of 1,198 workers interviewed. This differs from the other outcomes’ sample because sardars were not asked about children working at the kiln. * (*P* < 0.1), ** (*P* < 0.05), and *** (*P* < 0.01).

## 3. Conclusion

In this paper, we use a randomized controlled trial to study how a more efficient method of manufacturing bricks—and information about improving work conditions to incentivize adoption—influence coercive labor (including forced labor and labor trafficking), child labor, and work conditions in Bangladesh. Our setting provides an important test case for at least two reasons. First, coercive labor and child labor are thought to be pervasive in brick production in many countries (14). However, past studies documenting high prevalence rates at brick kilns in South Asia are generally based on small case studies of individual kilns (17, 26, 43–46). Among our study kilns, 50% of kilns had trafficked labor (and roughly 20% of workers were trafficked), and about 70% of kilns had child labor. Our analyses contribute important quantitative empirical evidence on the pervasiveness of labor exploitation in the brick sector in Bangladesh. Second, effective strategies to reduce coercive labor and child labor in environments with weak regulatory enforcement and state capacity like ours may require approaches that are aligned with the incentives of private business owners. However, we find little evidence that a profitable private sector innovation which improves kiln productivity/efficiency within a single brick season (or information about better work conditions to enhance its adoption) is sufficient to reduce labor trafficking or child labor in such settings.

Our findings that productivity gains did not improve short-term labor conditions in a coercive market (conditions which can feasibly change within a single brick season) also do not appear to be fully explained by unique market features of the brick sector in Bangladesh. Although we cannot be definitive about the reason for this lack of improvement in work conditions, a key factor that could help to explain these results in coercive labor markets is the exercise of monopsony power through the sardar system. Qualitative interviews that we conducted suggest that there is some degree of competition in brick labor markets—for example, workers are generally aware of work conditions and terms at nearby kilns, and alternative types of jobs are also available to kiln workers, including farming, fishing, operating rickshaws, masonry, and selling vegetables and goods in the informal sector. However, once workers arrive at kilns and begin the season, they face severe constraints to their ability to seek alternative employment or negotiate better terms, and they are typically working to pay debts owed to the sardar due to advance payment (sardars often come from the same villages as workers, creating informal enforcement mechanisms) (*SI Appendix*, Note 20). Our data show that 42.4% of workers experienced limited freedom of movement or communication (Fig. 2)–meaning that owners and sardars limit and/or supervise their movements and their communication. This lack of freedom creates a situation in which, conditional on workers agreeing to work at a kiln for a brick season, kiln owners exercise strong control over working conditions, effectively wielding monopsony power during the season (*SI Appendix*, Note 21). Under these conditions, even when worker productivity increases, owners appear largely able to capture the resulting surplus because, in practice, workers have no outside option or bargaining power during the season.

Overall, our findings suggest that productivity gains alone may be insufficient to improve work conditions in coercive labor markets, at least over shorter time horizons—and that absent more focused interventions, technological progress and productivity gains may do little to directly reduce labor exploitation in poorly regulated markets. Similarly, “light touch” strategies such as providing information to business owners about the benefits of positively incentivizing workers may also do little to improve labor standards. More generally, there is limited quantitative evidence on the factors responsible for coercive labor, including labor trafficking, as well as on strategies for effectively addressing them (13). This is a critical area for new empirical research.

## Supplementary Material

Appendix 01 (PDF)

## Data Availability

All data and code necessary to replicate the findings in this paper are provided in the Harvard Dataverse, along with complete instructions for replication (47). DOI: 10.7910/DVN/6OTWY2. All other data are included in the manuscript and/or *SI Appendix*.

## Footnotes

^*^Our study is partial equilibrium in nature, examining short-term effects. Therefore, it does not provide evidence on potential longer-term or general equilibrium effects that might originate through labor market-wide changes in wages, mobility, or outside options.

^†^Workers are often paid advances at the recruitment stage, but they are also paid in cash (including discretionary bonuses) during the season as well. Both pay, and a wide range of amenities that are central to work conditions, can reasonably be changed (based on managerial discretion) mid-season.

^‡^Specifically, *t* tests were done using the following variables: owner experience, owner education, existence of additional owners, knowledge of pilot intervention in Jashore, personal interaction with pilot kilns in Jashore, year of ZZK adoption, location, proximity to water, number of bricks fired in previous year, percent Class 1 bricks in the preceding year, production costs per thousand bricks, number of workers in each kiln job, and average weight of fired bricks.

^§^Among these kilns, 3 declined to participate in the study, 9 were closed by the government, and 36 ceased production early because of Ramadan (and so were not surveyed). See *SI Appendix*, Fig. A3 for more information. We note that this sample differs from the 276 in ref. 20 due to the timing of the worker survey and the kilns which were operating.

^¶^Our team addressed the complex issue of referring individuals (both children and adults) who are, or might be, suffering from harm to appropriate services in an environment in which trustworthy and reliable service providers are scarce. To develop our approach, we sought guidance from other researchers, non-governmental organizations (NGOs), and funding bodies involved in anti-trafficking work who have grappled with these challenges. This included qualitative interviews with workers, owners, and key informants in the area. The consensus from our planning work was that there is no universally recognized protocol for referrals and support, especially in the context of work at brick kilns which employ many seasonal migrant workers (the International Labor Organization (ILO) is currently developing guidelines for such circumstances). Given a lack of reliable support services in our study area, we decided to provide participants in our study with information about government hotlines, specifically advising about 109 telephone resources, and for rescue from worksites or other emergencies, 999 telephone resources. During our interviews with workers, we received no direct requests for assistance.

^#^Because we conducted our worker surveys near the end of the firing season, some brick molders had already left kilns for the season (molding bricks out of clay is the first step in the brick production process). To account for this, we developed a secondary sampling method. See *SI Appendix*, section A2 for more information about this method.

^‖^This method draws on the definition of human trafficking adopted by the United Nations Convention against Transnational Organized Crime’s Protocol to Prevent, Suppress and Punish Trafficking in Persons Especially Women and Children (Palermo Protocol) as well as the United States’ Trafficking Victims Protection Act (21). Labor trafficking in particular includes both debt bondage and forced labor. Debt bondage is defined as labor demanded as a means of servicing a loan or debt when the labor is undervalued and the debt is ill-defined or ever-increasing such that an individual cannot reasonably “repay” the debt. Forced labor is defined as labor coerced by means of violence or the threat of violence, restricted movement or confinement, or threats of punishment (21).

^**^Workers fearing retaliation may be reluctant to report adverse conditions, and thus underreport exploitative conditions. Underreporting is generally the central concern in measuring coercive labor at brick kilns in a variety of countries (15, 34). However, if workers perceived the presence of enumerators as an opportunity to advocate for improvements in work conditions, they could also potentially overreport exploitative conditions as well.

^††^Specifically, these are: being made to be available day and night without adequate compensation outside of the scope of the work agreement; performing hazardous and/or arduous services without proper protective gear; and living in degrading conditions (e.g. housing or shelter is unclean, provides no privacy, or is otherwise insufficient in a way that harms health).

^‡‡^We use the definition of hazardous conditions from the Worst Forms of Child Labor Convention of 1999: “Work which, by its nature or the circumstances in which it is carried out, is likely to harm the health, safety or morals of children.” Survey respondents are coded as observing child labor if they report that their own child works at the kiln or that at least one child under 18 works on their team at the kiln. Although the definition of child labor that we adopt requires that children ages 15 to 17 be working under “hazardous” conditions, given the universal lack of personal protective equipment (PPE) that we find among surveyed workers at study kilns (along with prior research on children in brick kilns; 17, 35, 36), we consider all jobs at study kilns to qualify as “hazardous.” This definition of child labor is also generally consistent with the Government of Bangladesh’s definition.

^§§^Although our preanalysis plan includes a correction for multiple hypothesis testing following Anderson (41) and using the Benjamini–Krieger–Yekutieli (42) sharpened two-stage procedure, we do not make this correction for labor trafficking indicators or cases given that the uncorrected estimates are statistically insignificant.

^¶¶^The share of child workers in each respondent’s team was not a prespecified outcome.

^##^In our preanalysis plan, we prespecified evaluating four outcomes jointly following Anderson (41) and the Benjamini–Krieger–Yekutieli (42) sharpened two-stage procedure: a binary outcome for labor trafficking at the kiln level, a binary outcome for child labor at the kiln level, a weighted count of indicators present at kilns, and a measure of the indicators targeted by the technical+incentive information arm at the kiln level. In our final analysis, we evaluate all outcomes both at the kiln level and at the worker level.
