# Supplementary material for: Productivity gains and work conditions in coercive labor markets: Experimental evidence from the Bangladesh brick sector
Source: Proc Natl Acad Sci U S A. 2026 Jul 16;123(29):e2528388123. doi: 10.1073/pnas.2528388123 (PMC13389626; doi:10.1073/pnas.2528388123)
Supplement: Supplementary file 1 — Appendix 01 (PDF) [file pnas.2528388123.sapp.pdf]

PNAS

Supporting Information for

Productivity Gains and Work Conditions in Coercive Labor Markets: Experimental Evidence from the Bangladesh Brick Sector

Grant Miller, Debashish Biswas, Aprajit Mahajan, Kimberly Singer Babiarz, Nina Brooks, Jessie Brunner, Sania Ashraf, Jack Shane, Alvise Scarabosio, Sameer Maithel, Shoeb Ahmed, Moogdho Mahzab, Mohammad Rofi Uddin, Mahbubur Rahman, and Stephen P. Luby

Corresponding Author: Grant Miller
E-mail: ngmiller@stanford.edu

This PDF file includes:

- Supporting text (SI Appendix Notes 1–21)
- Figs. A1 to A10
- Tables A1 to A7
- Worker Survey Questionnaire
- SI References

Contents

SI Appendix Notes . . . 2
A.1 Labor Trafficking Classification (Okech et al. 2025) . . . 5
A.2 Worker Sample Replacement . . . 7
A.3 Technical Intervention Details . . . 7
A.4 Incentive Information Given to Kiln Owners . . . 8
A.5 Responsibilities of Workers By Type . . . 12
A.5.1 Brick Molder . . . 12
A.5.2 Brick Loaders . . . 12
A.5.3 Firemen . . . 12
A.5.4 Brick Unloaders . . . 13
A.5.5 Managers . . . 13
A.5.6 Sardars . . . 13
A.5.7 Other Jobs at the Kiln . . . 13
A.6 Worker Survey Questionnaire . . . 35

List of Figures

A1 Map of Study Kiln Locations (Khulna Division) . . . 14
A2 Kilometers Traveled by Workers . . . 15
A3 Sample Selection Flow Chart . . . 16
A4 Study Timeline . . . 17
A5 Prevalence of Labor Trafficking Indicators at Study Kilns (Including “Liberal” Codings) . . . 18
A6 Prevalence of Labor Trafficking Indicators at Study Kilns - Control Arm (Including “Liberal” Codings) . . . 19
A7 Prevalence of Labor Trafficking Indicators at Study Kilns - Technical Arm (Including “Liberal” Codings) . . . 20
A8 Prevalence of Labor Trafficking Indicators at Study Kilns - Incentive Arm (Including “Liberal” Codings) . . . 21
A9 Share of Study Kilns by Number of Workers (Out of 6) Classified as Trafficked (Including “Liberal” Codings) . . . 22
A10 Robustness to Alternative Medium Indicators Weighting . . . 23

List of Tables

A1 Labor Trafficking Indicators based on Okech et al. (2025), with Survey Collection Status and Coding Variants . . . 24
A2 Balance Tests of Time-Invariant Kiln Characteristics . . . 29
A3 Balance Tests of Pre-Existing Worker Characteristics . . . 30
A4 Summary Statistics . . . 31
A5 ITT Estimates for Labor Trafficking by Study Arm (Liberal Coding) . . . 32
A6 Adoption and Impact of Technical Intervention on Efficiency and Productivity . . . 33
A7 Impact of Technical Intervention on Owner-Reported Costs . . . 34

SI Appendix Notes

SI Appendix Note 1. As defined in international law, labor trafficking (a form of human trafficking) generally occurs when a victim is compelled to provide labor through the use of force, fraud, or coercion, which can be subtle or overt, physical or psychological (United Nations 2000).

SI Appendix Note 2. If productivity raises the return to worker effort relative to the marginal cost of coercion, employers may increase coercion. Alternatively, productivity increases may decrease coercion by improving workers' outside options (Acemoglu et al. 2011; Ashraf et al. 2024; Domar 1970).

SI Appendix Note 3. Naidu et al. (2013) show that in 19th century Britain, the introduction of more productive technology sometimes increased coercive contract enforcement (to ensure stable labor supply during periods of peak demand). Ashraf et al. (2024) show that during Prussian industrialization, counties with greater elite-owned physical capital experienced faster serf emancipation. In recent experimental work (an exception in the literature), Sharma et al. (2024) show that alleviating liquidity constraints for micro-contractors in the Indian construction sector did not reduce forced labor.

SI Appendix Note 4. Global prevalence of coercive labor in brick production is difficult to estimate, but some estimate prevalence rates of bonded labor of 60% or higher (Kara 2014). Within Bangladesh, see Das et al. (2017) for information on work conditions at brick kilns.

SI Appendix Note 5. Bangladesh's Labour Act of 2006 formally outlawed labor for children under the age of 14 and hazardous labor (which can include work at brick kilns) for children under 18. However, due to the informal nature of most brick production, this law is not strongly enforced at brick kilns (Ministry of Labor and Employment 2010).

SI Appendix Note 6. Child labor is defined as any work by a child under the age of 18 which is hazardous, dangerous, or interferes with education. It also includes any child working under the age of 14, regardless of job (International Labour Organization 1973; International Labour Organization 1999).

SI Appendix Note 7. Zigzag kilns are a type of traditional coal-fired brick kiln prevalent in the informal sector in Bangladesh. They are considered "environmentally friendly" by the government and represent 70% of all kilns in the country (Brooks et al. 2024).

SI Appendix Note 8. "Class 1" bricks must have a "minimum compressive strength of 3,000 pounds per square inch, maximum water absorption of 20% dry weight after five hours of soaking in water, minimum weight of 3.5 kg per brick and the dimensions of 240 mm x 115 mm x 70 mm ... uniformly burnt, homogeneous in texture, uniform in color, free from cracks, nodules of free lime and other flaws, have plane rectangular faces with parallel sides and sharp straight right-angled edges" (Eil et al. 2020). "Class 2" bricks and lower relate to bricks that are of non-uniform colors, less uniformly burnt, and with deformed shapes or surface cracks (Eil et al. 2020).

SI Appendix Note 9. These improvements included: changing brick stacking patterns (air flows in a "zig-zag" pattern when bricks are stacked less densely) to improve airflow and combustion; more frequent coal feeding with smaller quantities of coal to improve combustion; closing kiln gates to reduce heat loss; creating a thicker ash layer to improve insulation; and using sawdust

or other biomass in front chambers to increase combustion efficiency (see Appendix Section A3 and Brooks et al. (2025)). Training also included separate sessions for firing and loading *sardars* followed by on-site assistance.

SI Appendix Note 10. Literature on the impact of anti-trafficking information campaigns is largely limited to campaigns targeting high-risk workers and the general public and focused on knowledge outcomes (see Henderson et al. (2024), Tjaden et al. (2021), and Zimmerman et al. (2021)). Boittin et al. (2025) evaluate the impact of informational interventions targeting both, finding that while knowledge in the general public improved, it did not among targeted migrant domestic workers.

SI Appendix Note 11. "Bonus" refers to any money paid over the agreed upon piece rate or salary, often at or near the end of the season. These are often conditional on reaching some quantity or quality of bricks produced. On the other hand, "Return bonus" refers to bonuses paid to workers who return to the same kiln in the following season.

SI Appendix Note 12. Other examples include: Liu et al. (2019), who show that Bangladeshi garment factories with better work conditions are considered more trustworthy by retailers; Luken et al. (2005), who show that targeted policies to meet corporate social responsibility requirements can improve short term profitability and long-term competitiveness; Adhvaryu et al. (2020), who show that improving work conditions (with more efficient lights that decrease indoor temperature on hot days) led to sizable energy savings and productivity gains; Harrison et al. (2010), who show that anti-sweatshop activism increased wages for workers, although these effects may have been offset by lower profits and greater risk of plant closure; and Verhoogen (2008), who shows that in Mexico's manufacturing sector, more productive firms can produce higher quality goods, and in turn pay higher wages to retain a high quality workforce.

SI Appendix Note 13. Following survey pre-testing and qualitative work, our team determined that a subset of indicators were both unlikely to be observed in our target population and were also likely to cause discomfort among respondents (for example, questions about sexual violence and hereditary slavery). These indicators were excluded from the survey; see Appendix Section A1 and Appendix Table A1 for a complete list of indicators and clarification of the ones we collected.

SI Appendix Note 14. For example, for degrading conditions, indicator 4 (see Appendix Section A1), the liberal coding requires that the housing provided at the kiln site harms health (no sanitation, etc.), while the conservative coding also requires that the individual was forced to live in employer-provided housing.

SI Appendix Note 15. See Hausman (2001) for a general discussion of estimation with measurement error in the dependent variable.

SI Appendix Note 16. We note that we prespecified an IV analysis, in which we use random assignment as an instrument for kilns adopting the technical intervention and estimate it using two-stage least squares. This prespecified IV analysis was intended for the kiln efficiency outcomes and the results are presented in Brooks et al. (2025). This analysis is not included here because the exclusion restriction may not hold in the case of the trafficking outcomes. In fact, the incentive information treatment may affect them directly, independent of adoption of the technical intervention.

SI Appendix Note 17. Although measurement differences prevent direct comparisons, the Bangladesh Labor Force Survey reports the share of workers across all sectors in Bangladesh lacking formal

labor contracts to be 84.9% in 2022 (Bangladesh Bureau of Statistics 2023).

SI Appendix Note 18. We note that although a majority of surveyed workers were in debt to their employers (70.3%), few workers described the debt in a way consistent with trafficking indicators. Only 0.5% report that they had not agreed to the debt and 0.1% considered their debt to be “high or increasing.”

SI Appendix Note 19. The only respiratory health outcome with an unadjusted statistically significant estimate is constant cough among workers in the technical-only arm ($p = 0.026$). However, this increase is insignificant after applying the Benjamini-Krieger-Yekutieli (Benjamini et al. 2006) sharpened two-stage procedure, which accounts for multiple respiratory-related outcomes.

SI Appendix Note 20. Such constraints have been documented in the kiln industry elsewhere in South Asia as well, see e.g., Guérin et al. (2007), Ercelawn et al. (2004), and Gupta (2003).

SI Appendix Note 21. The ability of kilns to replace workers mid-season varies to some extent by worker type. For more specialized workers such as firemen, it can be difficult for a kiln owner to replace a worker mid-season, while for less-skilled workers such as brick loaders or unloaders, it is relatively easier for kiln owners to find replacements during a season.

Section A.1: Labor Trafficking Classification (Okech et al. 2025)

Following the framework developed by Okech et al. (2020) and Okech et al. (2025), we classify workers as victims of labor trafficking if they meet one of three thresholds. Note that this framework encompasses all forms of human trafficking, and we list all indicators included in Okech et al. (2025) for completeness. However, this paper only measures and studies labor trafficking (not sex trafficking and other forms of human trafficking). Table A1 shows these indicators, noting which were collected in our survey and which have liberal and conservative codings (note that liberal and conservative codings are ours, not from Okech et al. (2025)).

- **Threshold 1**
  - Worker subject to any **ONE** of the following *extreme* indicators:
    - No freedom of movement or communication (FM3)
    - Made to work in commercial sex to repay debt or wage advance (PL3)
    - Ever been sold for labor or commercial sex work (V2)
    - Tradition or birth into slavery or bondage (DD2)
- **Threshold 2**
  - Worker is subject to *strong* type conditions from at least **TWO** of the following seven domains:
    1. **Recruitment:** Coercive (R1) or deceptive recruitment regarding nature of services (R2).
    2. **Employment Practices:** Had pay withheld and if worker quits they will not receive wages (EP1), or worker had high debt related to employment (EP2).
    3. **Personal Life:** Employer has control (PL1) or transferred control over a meaningful part of the worker's personal life (PL2).
    4. **Degrading Conditions:** Made to engage in illicit activities (DC3), or made to be available day and night without adequate compensation (DC1).
       - Conservative Coding (DC1): Worker is always required to work overtime and never receives compensation for it.
       - Liberal Coding (DC1): Worker is always or sometimes required to work overtime and never or sometimes receives compensation for it.
    5. **Freedom of Movement:** Constant surveillance of personal space (FM2) or confiscation of documents/identification papers (FM1).
       - Conservative Coding (FM1): Employer often confiscates documents.
       - Liberal Coding (FM1): Employer often or sometimes confiscates documents.
    6. **Debt & Dependency:** Debt was imposed on the worker without their consent (DD1).
    7. **Violence:** Worker witnessed physical violence against another worker (V1) or experienced physical (V3) or sexual violence (V4) against themselves or someone they care about.

- **Threshold 3**
  - Worker subject to **ONE** *strong* type condition from the above seven domains from Threshold 2

  AND

  - Worker subject to any **THREE** of the following *medium* type conditions:
    - Recruiter deceptive about living or working conditions (R3)
    - Paid recruitment fees (R4)
    - High or increasing debt from recruitment (EP3)
    - Made to work overtime beyond legal limits (EP4)
    - Absence of a formal contract (EP8)
    - Ever not received wages or had wages withheld (EP6)
    - Made to perform additional services outside contract or work overtime beyond legal limits (EP5)
      - Conservative Coding: Worker never receives overtime compensation
      - Liberal Coding: Worker sometimes or never receives overtime compensation
    - Recruitment linked to debt (EP7)
    - Made to work or engage in commercial sex for employer's private home or family (PL4)
    - Confiscation of mobile phones as a means of control (PL5)
      - Conservative Coding: Employer often confiscates communication devices
      - Liberal Coding: Employer often or sometimes confiscates communication devices
    - Hazardous labor without protective equipment (DC2)
      - Conservative Coding: No PPE provided and respondent said that they were exposed to dangerous work
      - Liberal Coding: Respondent stated they did not receive PPE, or did not receive gloves, safety shoes, and goggles (the three most important PPE items for work at brick kilns)
    - Made to live in degrading or inhumane conditions (DC4)
      - Conservative Coding: Housing does not have toilet, electricity, or privacy, and the respondent is forced to live in employer-supplied housing
      - Liberal Coding: Housing does not have toilet, electricity, or privacy
    - Limited freedom of movement or communication (FM4)
    - Constant surveillance at work (FM5)
    - Unable to refuse to provide services (DD4)
    - Threat of reporting to authorities (V5)
    - Emotional or psychological abuse (V6)
    - Threat of harm to personal or professional reputation (V7)
    - Threat of violence against you or someone you care deeply about (V8)

These thresholds are also represented graphically in Figure 1 in the main text.

Section A.2: Worker Sample Replacement

In our study protocol, we planned to randomly sample and survey six workers at each kiln (one sardar, one firemen, one brick molder, two brick loaders, and one brick unloader). However, given differences in their responsibilities, different types of workers at kilns are present at different times during the brick production process. At the time of our survey, some brick molders (the most common job in brick kilns, and generally the first to finish their work during a season) had already left kilns for the season. Additionally, some kilns closed early due to the timing of the end of Ramadan and the celebration of Eid al-Fitr. We therefore developed a replacement protocol for sampling workers when it was not possible to follow our original protocol. Specifically, if the planned number of brick molders, loaders, or unloaders could not be surveyed, we instructed enumerators to replace them with a different type of worker (excluding firemen) under a different sardar. Alternatively, if the planned number of firemen could not be surveyed, we instructed enumerators to replace them with any other type of worker.

Section A.3: Technical Intervention Details

Less dense brick stacking with multiple (two or three) zigzag air paths
The technical intervention introduced less dense brick stacking with multiple zigzag air paths, where existing practice was to densely pack bricks with only a single zigzag path for air to travel. This change allows for better distribution of air flow, leading to uniform distribution of heat and combustion of coal, therefore decreasing pollutant emissions from coal combustion. Additionally, this change also better maintains pressure, meaning that less energy is required to operate the fan blowing air through the kiln.

Single fireman continuous fuel feeding
In brick kilns, coal is fed through feed holes on the kiln's roof by firemen. The prevailing method of coal feeding is for three to four firemen to feed the kiln at intermittent intervals (feeding interval of 10-15 minutes, followed by a non-feeding interval of 15-20 minutes). This method leads to accumulation of fuel in the kiln, hampering complete combustion of the coal. In addition to incomplete combustion, accumulation of coal also leads to higher particulate emissions.

The technical intervention changes this method so that a single fireman continuously feeds the kiln for 30 minutes, after which the fireman stops and switches with his partner. With this method, fuel is fed in smaller quantities, allowing for adequate air and complete combustion. This allows for less wasted fuel and lower pollutant emissions. It also causes more uniform heat distribution across the kiln (and more consistent brick quality).

These first two interventions are the most important for improving operation of a kiln. Kilns observed adopting both practices were coded as adopters of the technical intervention.

Thicker ash layer on the kiln top
The layer of ash on the top of the kiln serves as a roof, providing insulation against heat loss. The prevailing practice is to have a 6-inch ash layer, but the technical intervention advocated increasing the thickness of the ash layer to 9 inches or more. This improvement in insulation reduces the fuel needed to fire bricks and increases the temperature among bricks on the top layer, increasing the

share of Class-1 bricks (i.e., the best quality bricks).

Closing kiln entry gates with an ash-filled cavity wall
Kiln gates allow workers to enter the kiln to stack "green bricks" and remove fired ones. When the bricks are being fired, these gates are closed and sealed, and the prevailing practice is to build a temporary wall one brick thick (roughly 10 inches). The intervention encouraged kilns to increase the thickness of this wall to 30 inches, including an inner wall of 15 inches, a 5-inch layer of ash for insulation, and an outer wall 10 inches thick. This new method increases insulation, decreases fuel requirements, and allows bricks stacked near kiln gates to reach high temperatures, increasing the share of Class-1 bricks.

Use of powdered biomass fuel in the newly inducted chamber in the fuel-feeding zone
In a zigzag kiln, fire moves through the kiln's firing chamber. As the fire moves, a new chamber enters the fuel-feeding zone every 8-12 hours. The temperature of a newly-inducted chamber is initially lower (<500°C). By prevailing practice, coal is fed into the newly-inducted chamber, but because the newly-inducted chamber has a low temperature, this coal does not burn completely, increasing pollutant emissions. The intervention encouraged kilns to feed sawdust and other powdery biomass with high concentrations of volatile matter and low ignition temperatures into the newly-inducted chamber. Because these fuels burn completely at lower temperatures, they increase a new chamber's temperature until it reaches 700°C, when coal is added.

Section A.4: Incentive Information Given to Kiln Owners

Kilns that were randomized into the technical+incentive arm received a detailed information session along with the hands-on training provided with the technical intervention. In these information sessions, our team described how our pilot work increased brick quality while decreasing fuel use, and that achieving these benefits depends on the ability to align worker incentives with the new production method, providing evidence that pilot firms that improved work conditions (either through higher wages or bonuses, or in-kind transfers) experienced greater benefits. The complete script is as follows:

[Begin Script]

I'm here to talk to you about how you can get more profit in this year's brick production. We are glad you are working with us to implement the new practices, but their success depends on every worker on your kiln. Our team is here to help with technical training and assistance to make sure your workers have the proper skills to implement everything correctly. **If everyone on your kiln works together and follows the instructions, you will use less coal and increase your production of Class-1 bricks. As a result, these new practices will increase your profit and your kiln will be more successful.**

How do we know this?

Our team worked with similar brick kilns in Jashore, and owners saw a 14% increase in the percentage of Class-1 bricks and a 20% reduction in coal spending per brick in kilns that successfully followed the recommended practices of single fireman continuous coal feeding and double zigzag brick setting, compared to kilns using traditional methods.

What's more interesting is that the owners from Jashore that provided more incentives and benefits to their workers had even higher Class-1 bricks (on average, 5 percentage points higher) and lower coal spending (on average, 0.42 Taka less per brick) compared to kilns that did not offer additional incentives.

How can you reap the same benefits?

The workers on your kiln are crucial for the success of this new practice. They have to learn the new practices and at first they may not want to change from the old way of doing things. If your workers invest the time to master the new skills it will lead to huge benefits for you. Now, you can imagine when they are learning the new practices they might work more slowly, which might reduce their pay. If they do not feel motivated to adopt the new practices, they may take shortcuts or not learn it properly unless you find a way to include them in the success you will have from these new practices.

You may also consider the time and effort you are putting in to having your workers trained on these new practices. They are learning many new skills which will make your kiln successful. You will benefit if you can use the same workers next season, because they will already have the experience and training on these new practices. If you can encourage workers to return, it will be very beneficial to your kiln operation and production.

Because all workers on your kiln must successfully adopt these practices and work together to increase your production and profit, we recommend any incentives or extra bonus be offered to all workers.

We have some suggestions that other kiln owners like you have used and found to be successful at increasing their kiln performance, getting better performance from workers, and commitments from workers to return to the same kiln:

1. Providing some extra monetary incentives to the workers to motivate them to follow this new practice properly. This will be easily covered by your increased profit/production soon. Because all workers on your kiln must successfully adopt these practices and work together to increase your production and profit, we recommend incentives be offered to all workers. Successful kiln owners have used incentives differently for different categories of workers, for example, firing workers are given lump sum bonuses after a circuit, whereas unloaders and loaders are given bonuses in terms of 1000 bricks.

2. There are easy improvements you can make for your workers to make them happier and healthier to motivate them be more productive. If your kiln gets a reputation for being a good place to work, where workers are well-taken care of, your workers are more likely to return next season and more workers will want to work for you.

How can you make incentives and benefits work for you?

When offering these incentives, it is very important that the workers themselves receive the benefit. Otherwise, they will not be motivated to adopt the new practices, trust will be lost, and your kiln will not benefit. You may encourage the Sardars to provide these benefits to workers so that the workers will adopt the practices. Some owners provide benefits directly to the workers to make sure they receive them. A common practice of successful owners is to announce a particular day and time and request all workers and sardars be present, then owners hand over bonuses/bakhshish by themselves. This practice is successful because everyone will give credit to the owner for the extra benefits.

It is also important that you provide the incentives and benefits in a timely manner and early in the season. If it is too late, the workers may not be encouraged to follow the new practices and you will not see the benefit in time.

[Ask: Any questions on what we have talked about so far?]

What are examples of monetary incentives and good working conditions that you can provide?

We have put together a list of suggestions from successful kiln owners for you to think about:

Monetary incentives:

1. You may offer a 'Bakhshish' from the higher earnings that you will get by adopting our suggested practices. For example, you can offer a Bakhshish to your workers such as 5-10%, which can be shared across all the workers. One successful kiln owner has provided 10000 Tk to the loading Sardar for adopting the new system and he committed to providing it subsequently in the next rounds of brick stacking. If you inform them at the beginning of each circuit about the Bakhshish and the importance of following the new practices to achieve a higher amount, it will motivate their performance during the circuit.

2. You may offer a bonus (onudan) to the workers if your kiln achieves a certain level of class-1 bricks in each circuit. We have provided a guideline for the bonuses depending on the share of class-1 bricks. For example, you may offer BDT 5000 if your kiln achieves 80-85% class-1 bricks in a cycle, BDT 6000 if you achieve 85-90% class-1 bricks, and BDT 7000 if you achieve >90% class-1 bricks. You can adjust the schedule given your kiln's performance. We suggest you inform workers at the beginning of the circuit about the bonus to motivate their performance and deliver the payment at the end of the circuit once the brick quality has been assessed.

3. You can also provide 'Bakhshish' of extra Taka 50 per 1000 bricks if your kiln achieves 80-85% class-1 bricks, extra Taka 100 per 1000 bricks if your kiln achieves 85-90% class-1 bricks, or extra Taka 150 per 1000 bricks if your kiln achieves >90% class-1 bricks.

4. Some of the recommended practices will require more time involvement for the workers. For example, in the new method, workers need to increase the ash layers by 9-12 inches from the previous setting. In the new method, fire travels faster and more loading of bricks is necessary to keep up the fire travel in a circuit. In both cases, you can consider increasing the wages of the workers by Taka 10-50 per 1000 bricks to account for the changes.

5. You may offer a return bonus if workers return to your kiln the next season. Inform them of the bonus offer before the end of the current season, so that it can encourage them to return the next year. For example, some kiln owners have offered a bonus equal to 20% of the workers current wages if they return the following season, which will be paid only after they return.

6. You might see that some of your workers want to leave for other working options during the firing season, especially on agricultural fields. To prevent workers who have been trained on these new and improved practices from leaving in the middle of an active season, kiln owners have provided instant bonuses in cash. By making your kiln a more desirable and better paying place to work, the workers will not want to leave for other options.

7. Many kiln owners have successfully retained a higher presence of workers by offering 'attendance bonuses.' You can offer some bonuses for the top 5 workers who are most regular in your kilns to motivate all the workers to avoid shirking.

Working conditions:

You will know best what type of working conditions are the most important for your workers, but we have put together a list of suggestions from successful kiln owners for you to think about:

1. You can provide shaded/resting areas for your workers. If workers rest in their free time, this can improve their productivity during the rest of the day.
2. You can provide accommodation for your workers. As you know, many of your workers have migrated from other places to work here. Providing accommodation facilities (spacious room, individual beds, windows, ventilation, hygienic toilets, electricity, and cooling/ceiling fan) would benefit the workers and increase their productivity.
3. Successful kiln owners in Jashore have offered improved meals like chicken or beef to their workers if they achieve good performance of class-1 bricks or without any condition.
4. Some kiln owners provide new clothing to their workers during religious festivals like Eid or Pahela Baisakh or during the winter season.
5. Workers' health is one of the most important aspects of worker productivity and success that you can improve as a motivated kiln owner. Workers especially firemen may be provided with saline to help them from dehydration. You can help workers to go to the nearest community clinics, and union and upazila health complexes if they have any medical needs.
6. To help prevent against injuries and accidents that will harm your workers and your production, it is important that workers have proper protective equipment. We suggest heat protective boots, masks, gloves and if possible, movable shed for firemen, masks and customized helmet for unloaders, masks for the brick loaders and ash layer providers. Providing such protective equipment will make workers feel protected and cared for and will motivate their production.
7. Workers may be concerned about their children's schooling while the kiln season is in progress. Bangladesh government has made primary schooling free to access. You can encourage and help workers to get their children admitted to the nearest government school. Also, if NGO schools (i.e. BRAC) are nearby, you can also encourage workers to send their kids to those schools.
8. Bangladesh government has recently reduced the price of the LPG cylinder gas. If your kiln does not have a pipeline gas connection, then you can provide LPG cylinder gas to the workers to facilitate cooking.
9. Many workers, especially firemen, come from an outside district and they stay at the kiln throughout the season apart from their families. Offering a monthly/quarterly leave to these workers can be helpful to meet with their families for refreshment and they will return to your kiln happy and motivated.

Which one of these do you think is feasible for you to do?

[Ask: owners to raise hands for different options and note their answers]

How can we help you think through it?

Closing pitch: Remember, by adopting these new practices your kiln will use less coal and produce more class 1 bricks, but their success depends on every worker on your kiln. By offering extra incentives or improved working conditions to your workers will encourage quicker learning and successful adoption of the new practices. This will not only increase your profit this season, but it may also help you retain your experienced workers for next year. For the incentives to successfully motivate workers and improve their performance, it is very important that you provide them to all workers and you offer them in a timely manner. If they follow the new practices, it means more profit for you, and everyone will benefit.

[End Script]

Section A.5: Responsibilities of Workers By Type

A.5.1: Brick Molder

Brick molders' main job is to shape clay into the molds of bricks, termed "green bricks." Due to the limited skill requirements, as well as lower levels of risk, this is the job most commonly held by women and children in the kilns. Additionally, the molder's role is mainly done at the beginning of the season, after which many will return home. They are also the majority of workers at a kiln (see Table A4).

A.5.2: Brick Loaders

Once molding is completed, the next task is to load the "green bricks" into the kiln. The brick loaders take the "green bricks" from the field and then load them into the kilns in a specific pattern. In our intervention, loaders played an important role as they had to follow a double/triple zig-zag structure to load the bricks. After brick molders, brick loaders constitute the highest number of workers in the kiln.

A.5.3: Firemen

Firemen are the workers with the most technical skills in a brick kiln. They normally get paid the highest (after sardars). Typically there are ten firemen in a kiln and they are always men. Firemen are responsible for feeding coals into the kiln system. This process is critical as the quality of the bricks mostly depends on how the fire is traveling in a kiln and how the temperature is distributed. In our intervention, one major component was to use single-fireman continuous feeding. The success of our technical intervention in part depended on the efficiency of the firemen's work.

A.5.4: Brick Unloaders

Once the bricks baking is done, a group of workers known as brick unloaders take the bricks out of the kiln and put them in a separate place to cool down. This process requires intense physical labor to carry heavy bricks out of the kiln. Brick unloading is mostly done at the end of every production cycle. Unloading is important to maintain the quality of the bricks as sometimes while unloading many of the bricks lose their intended shapes, lessening their value.

A.5.5: Managers

Managers are the most powerful individuals at the kiln after the owner. They oversee the entire production process, but do not engage in any labor with any particular team. They work directly with the owners and in the absence of the owners, they make executive decisions. The owners pay them either monthly or by the season. Typically there would be at least one manager, but it could go up to four or five in different kilns.

A.5.6: Sardars

Sardars are team leaders for each of the main teams at a kiln (i.e., Brick Loaders, Brick Unloaders, Firemen, and Molders). They help the team work efficiently and distribute payments from the owner to workers.

A.5.7: Other Jobs at the Kiln

Although the aforementioned jobs are the most common at kilns, there are many additional jobs that workers can also perform at the kiln. These include coal crushers, electricians, carpenters, and night guards. Kilns will also often have 10-12 daily laborers. These workers have a very loosely defined job, often including cleaning, meal preparation, and covering any needs in the production process.

Figure A1: Map of Study Kiln Locations (Khulna Division)

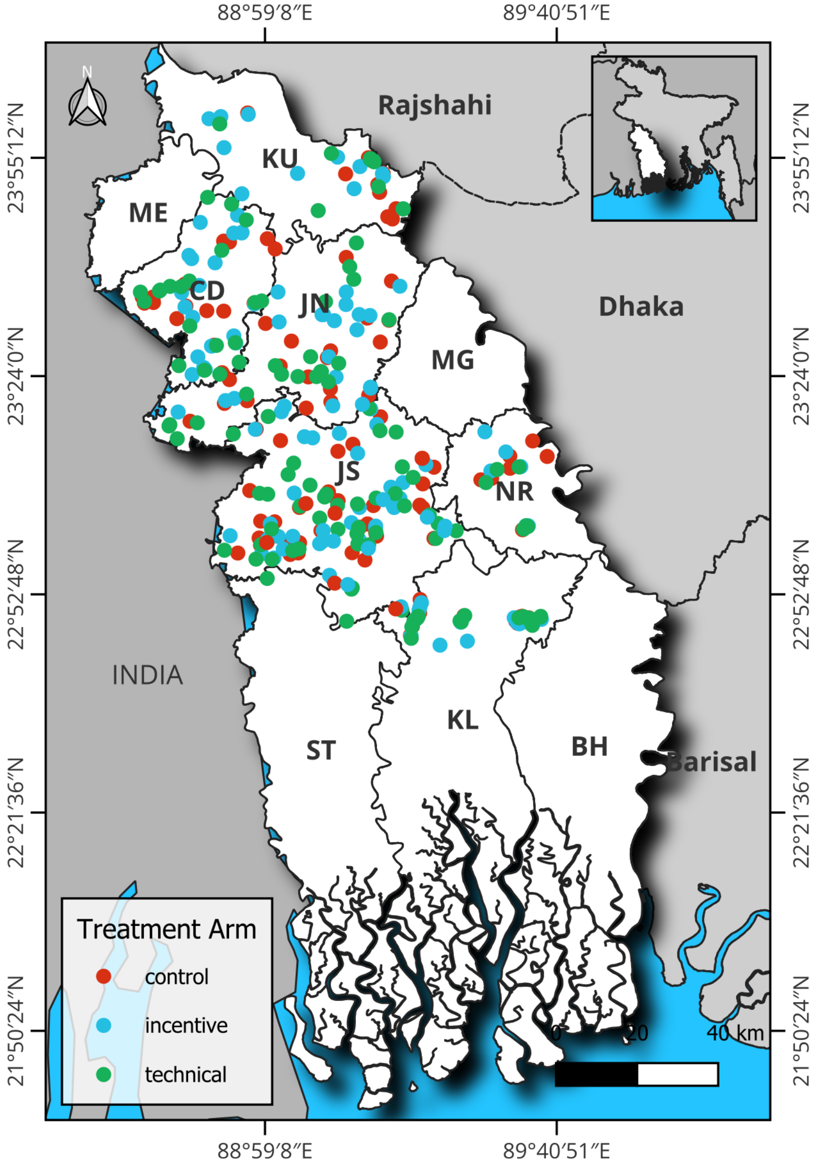

Figure A2: Kilometers Traveled by Workers

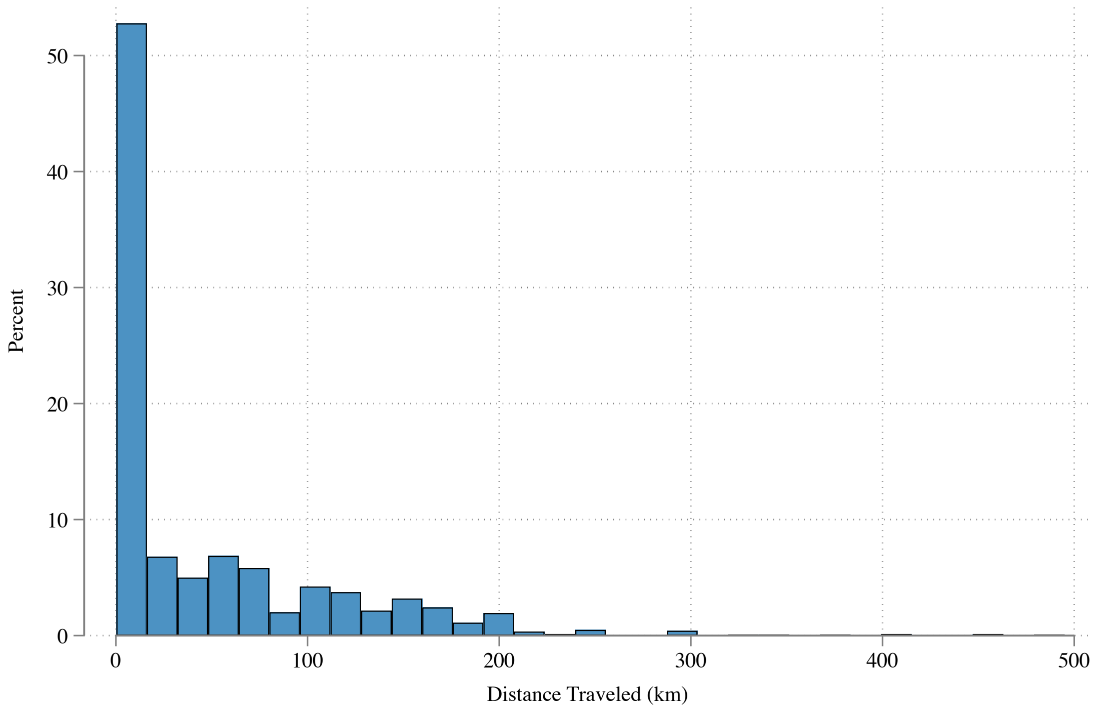

Figure A3: Sample Selection Flow Chart

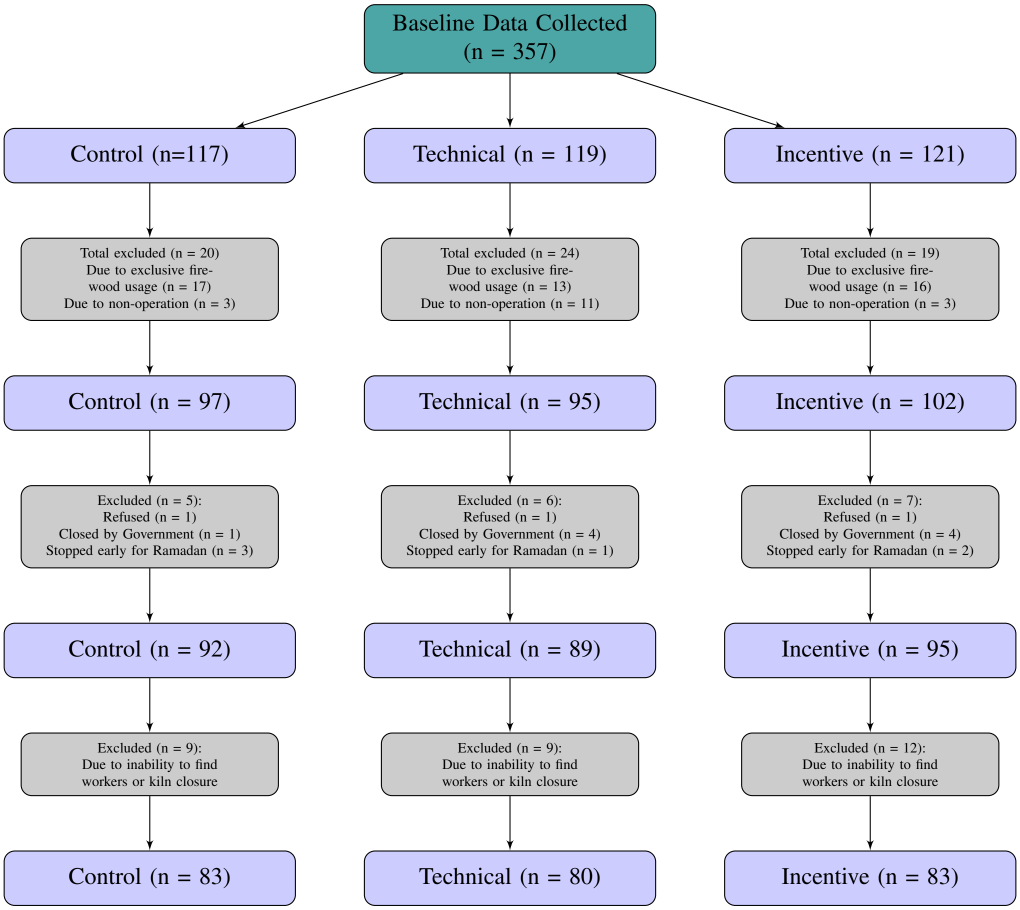

Figure A4: Study Timeline

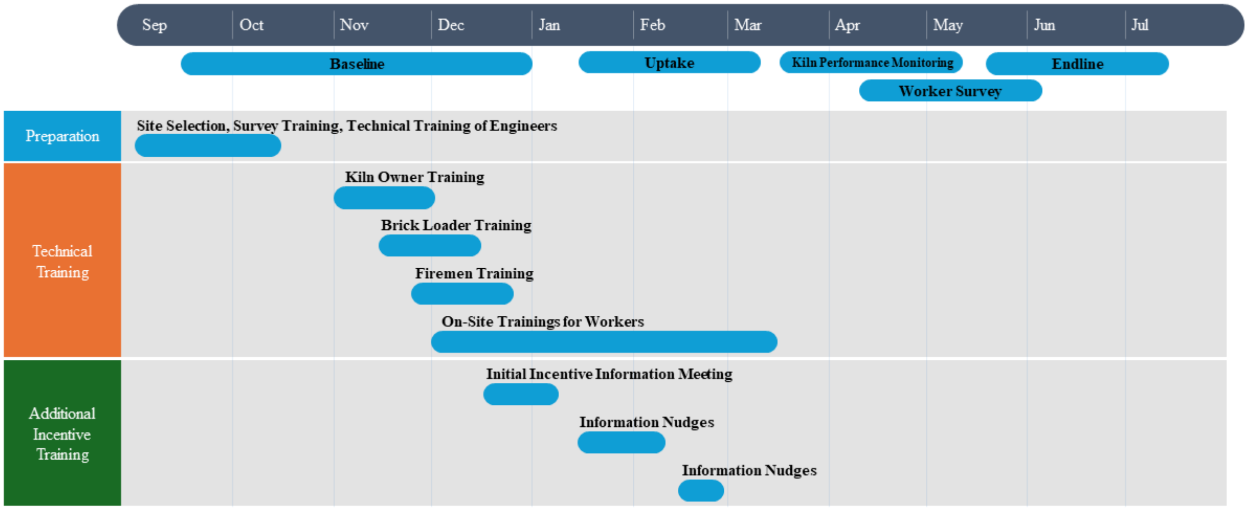

Figure A5: Prevalence of Labor Trafficking Indicators at Study Kilns (Including "Liberal" Codings)

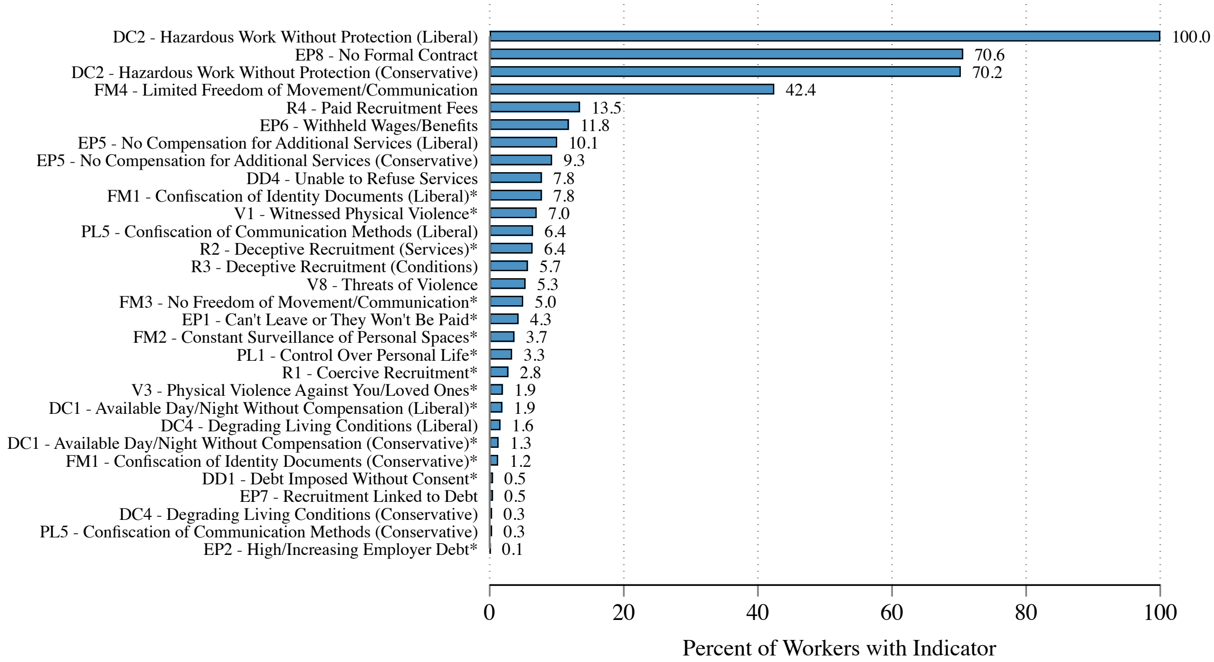

* = Strong Indicator. Sample includes data from 246 brick kilns, with a total of 1442 workers interviewed. All indicators come from Okech et al. (2020). Note that not all indicators mentioned in Okech et al. (2020) are included here, because our survey does not ask about sex trafficking or sexual violence. The graph shows both the conservative and the liberal coding of indicators requiring subjective judgment. See Appendix Section A1 for more information on trafficking

Figure A6: Prevalence of Labor Trafficking Indicators at Study Kilns - Control Arm (Including “Liberal” Codings)

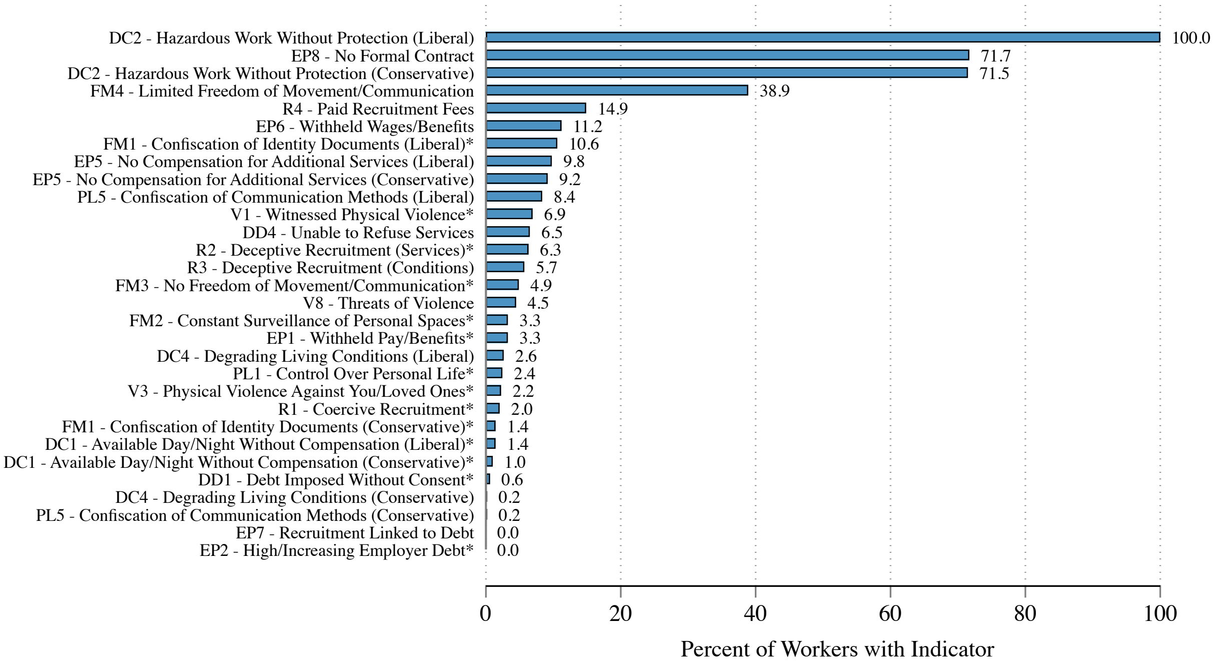

* = Strong Indicator. Control kiln sample includes data from 83 brick kilns, with a total of 490 workers interviewed. Note that not all indicators mentioned in Okech et al. (2020) are included here, because our survey does not ask about sex trafficking or sexual violence. The graph shows both the conservative and the liberal coding of indicators requiring subjective judgment. See Appendix Section A1 for more information on trafficking indicators.

Figure A7: Prevalence of Labor Trafficking Indicators at Study Kilns - Technical Arm (Including "Liberal" Codings)

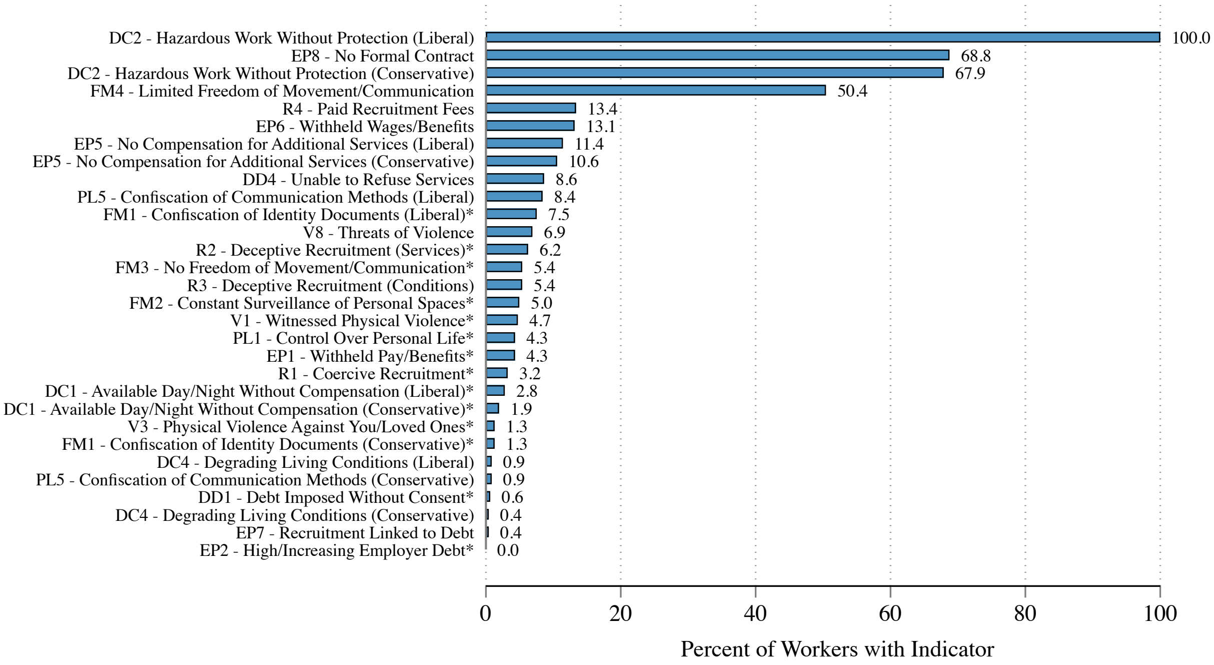

* = Strong Indicator. Technical kiln sample includes data from 81 brick kilns, with a total of 465 workers interviewed. Note that not all indicators mentioned in Okech et al. (2020) are included here, because our survey does not ask about sex trafficking or sexual violence. The graph shows both the conservative and the liberal coding of indicators requiring subjective judgment. See Appendix Section A1 for more information on trafficking indicators.

Figure A8: Prevalence of Labor Trafficking Indicators at Study Kilns - Incentive Arm (Including "Liberal" Codings)

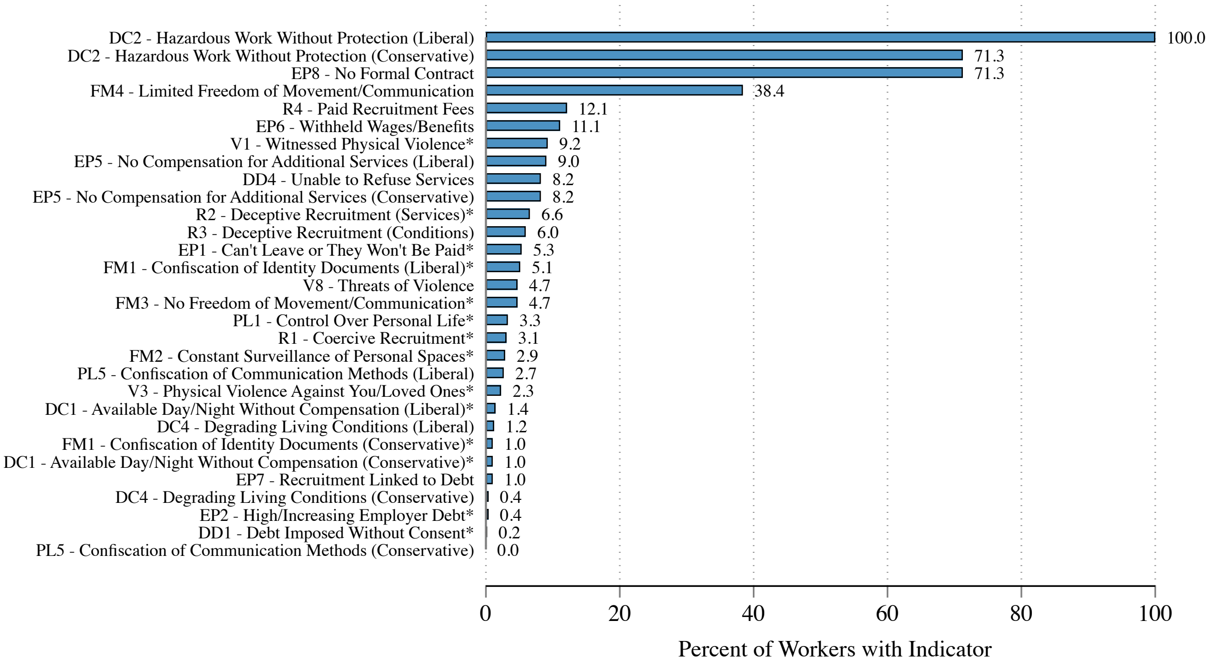

* = Strong Indicator. Technical+incentive kiln sample includes data from 82 brick kilns, with a total of 487 workers interviewed. Note that not all indicators mentioned in Okech et al. (2020) are included here, because our survey does not ask about sex trafficking or sexual violence. The graph shows both the conservative and the liberal coding of indicators requiring subjective judgment. See Appendix Section A1 for more information on trafficking indicators.

Figure A9: Share of Study Kilns by Number of Workers (Out of 6) Classified as Trafficked (Including “Liberal” Codings)

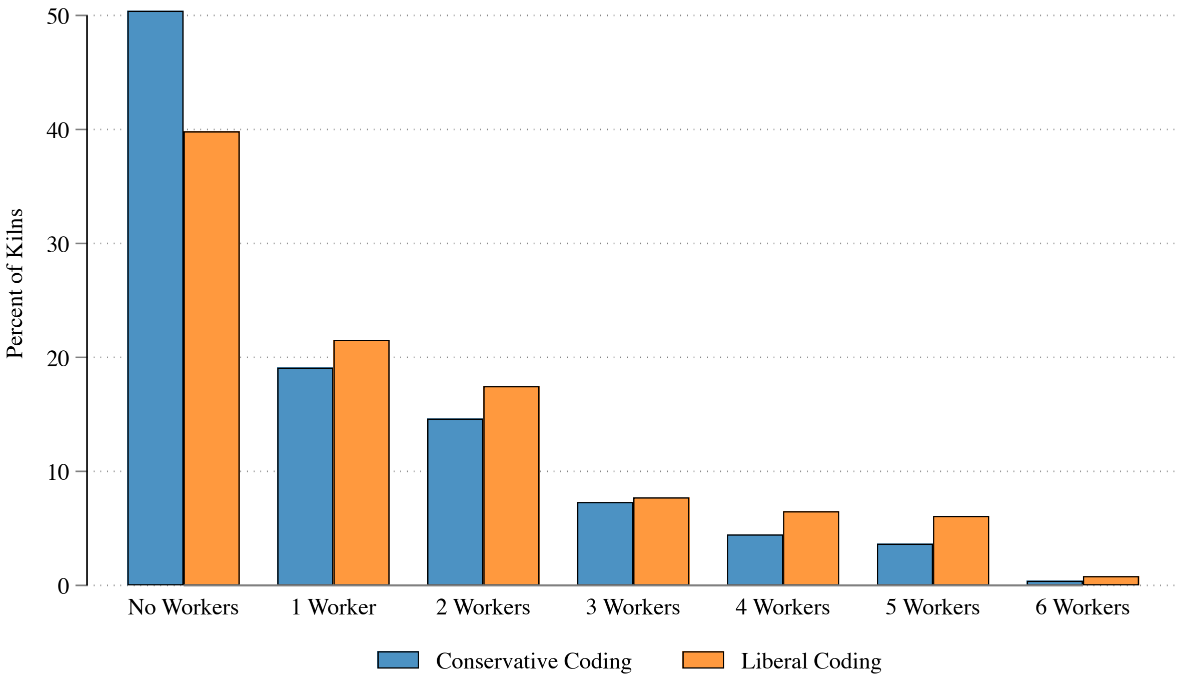

Sample includes data from 246 brick kilns, with a total of 1442 workers interviewed. Trafficking classifications follow Okech et al. (2020). See Appendix Section A1 for more information on trafficking classifications. 'Conservative Coding' places stricter requirements on indicators which have a degree of subjectivity, while 'Liberal Coding' places weaker requirements.

Figure A10: Robustness to Alternative Medium Indicators Weighting

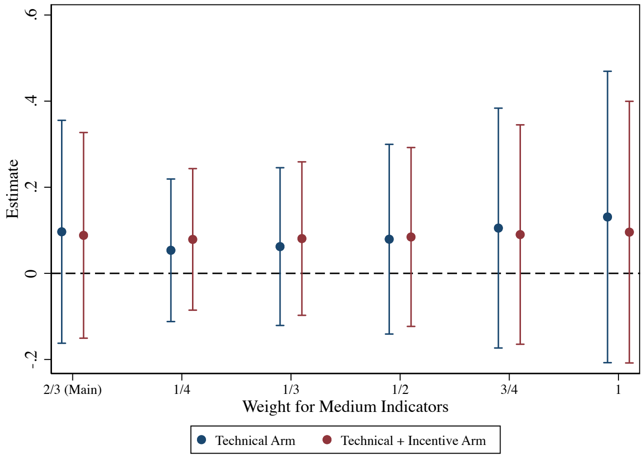

(a) All Indicators (Worker)

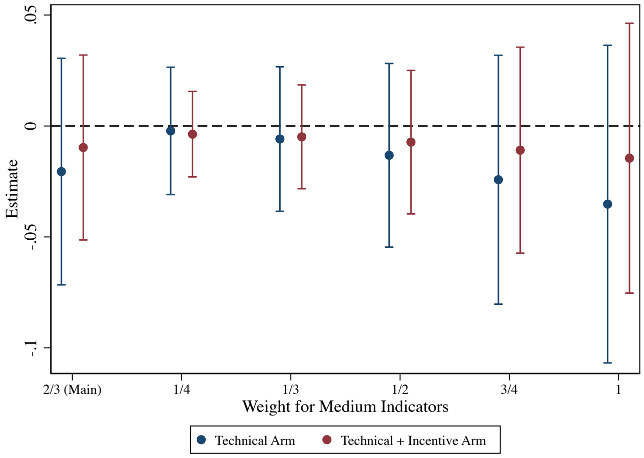

(b) Target Indicators (Worker)

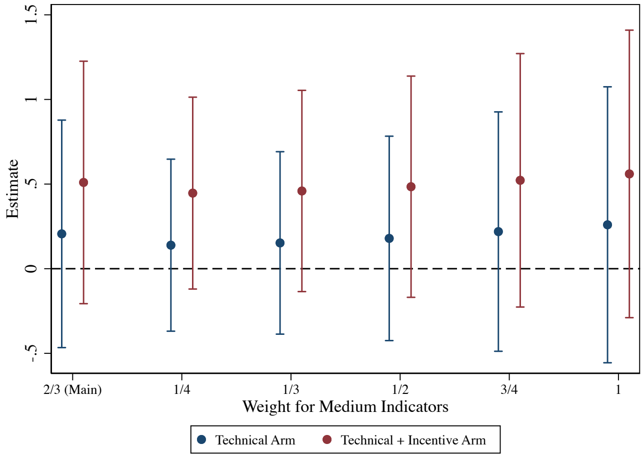

(c) All Indicators (Kiln)

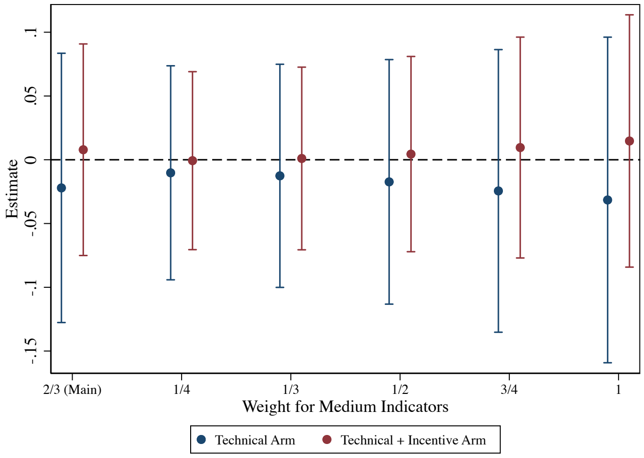

(d) Target Indicators (Kiln)

Notes: Sample: 246 kilns (1442 workers) surveyed in both the worker survey as well as kiln performance monitoring. Graphs show the OLS estimates and 95% confidence intervals generated from regressing the first four outcomes in Table 1 on treatment arm dummy variables with randomization strata fixed effects.

Table A1: Labor Trafficking Indicators based on Okech et al. (2025), with Survey Collection Status and Coding Variants

**Code**	**Type**	**Indicator Description**	**Collected in Survey?**	**Has Coding Variants? (Conservative and Liberal)**
**Recruitment**				
R1	Strong	Coercive recruitment (abduction, confinement during the recruitment process)	Yes	No
R2	Strong	Deceptive recruitment (nature of services or responsibilities required)	Yes	No
R3	Medium	Deceptive recruitment (regarding working conditions, content or legality of relevant contract, housing and living conditions, legal documentation or acquisition of legal status, location or employer, compensation/benefits, promise of marriage/love)	Yes	No
R4	Medium	Paid recruitment fees	Yes	No
**Employment Practices & Penalties**				
EP1	Strong	Had your pay, other promised compensation and/or benefits withheld and if you leave you will not get them	Yes	No
EP2	Strong	High or increasing debt related to an employer or other person who controls earnings (by falsification of accounts, inflated prices for goods/services purchased, reduced value of goods/services produced, excessive interest rate on loans, etc.)	Yes	No

Continued on next page

Table A1 – continued from previous page

Code	Type	Indicator Description	Collected in Survey?	Has Coding Variants? (Conservative and Liberal)
EP3	Medium	High or increasing debt related to a recruiter, intermediary or other individual (by falsification of accounts, etc.)	No	N/A
EP4	Medium	Made to work overtime beyond legal limits	No	N/A
EP5	Medium	Made to perform additional services or responsibilities (beyond what was agreed) without due compensation	Yes	Yes
EP6	Medium	Ever not received or had withheld promised wages, benefits, or other compensation.	Yes	No
EP7	Medium	Recruitment linked to debt (advance or loan)	Yes	No
EP8	Medium	Absence of a formal contract	Yes	No
**Personal Life & Properties**				
PL1	Strong	Another individual has control over any meaningful part of your personal life (i.e. blackmail, religious retribution, or exclusion from future employment, community, personal or social life, etc.)	Yes	No
PL2	Strong	Another individual has transferred control over any meaningful part of your personal life.	No	N/A
PL3	Extreme	Made to work or engage in commercial sex in order to repay outstanding debt or wage advance	No	N/A

Continued on next page

Table A1 – continued from previous page

Code	Type	Indicator Description	Collected in Survey?	Has Coding Variants? (Conservative and Liberal)
PL4	Medium	Made to work or engage in commercial sex for employer's private home or family	No	N/A
PL5	Medium	Confiscation of mobile phones or other communication methods as a way to have control over you.	Yes	Yes
PL6	Medium	Confiscation of other valuables as a way to have control over you.	No	N/A
**Degrading Conditions**				
DC1	Strong	Made to be available day and night without adequate compensation outside of the scope of the contract	Yes	Yes
DC2	Medium	Made to complete hazardous and/or arduous services without proper protective gear	Yes	Yes
DC3	Strong	Made to engage in illicit activities	No	N/A
DC4	Medium	Made to live in degrading conditions e.g. housing or shelter is unclean, provides no privacy, or is otherwise insufficient in a way that harms your health	Yes	Yes
**Freedom of Movement**				
FM1	Strong	Confiscation of or loss of access to identity papers or travel documents	Yes	Yes
FM2	Strong	Constant surveillance of personal spaces by employer, recruiter, or other individuals	Yes	No

Continued on next page

Table A1 – continued from previous page

Code	Type	Indicator Description	Collected in Survey?	Has Coding Variants? (Conservative and Liberal)
FM3	Extreme	No freedom of movement or communication	Yes	No
FM4	Medium	Limited freedom of movement or communication i.e. supervised communication, movement restricted or surveilled during off-hours.	Yes	No
FM5	Medium	Constant surveillance of place of work	No	N/A
**Debt or Dependency**				
DD1	Strong	Had a debt imposed on you without your consent	Yes	No
DD2	Extreme	Tradition, birth/descent into hereditary slavery or bonded status	No	N/A
DD3	Medium	Pre-existence of an intimate or dependent relationship such as romantic or familial relationship	No	N/A
DD4	Medium	Unable to refuse to provide services	Yes	No
**Violence & Threats of Violence**				
V1	Strong	Physical violence inflicted in front of you on other individuals	Yes	No
V2	Extreme	Ever been sold or witness ownership of another person in your situation being sold for labor or for sex	No	N/A
V3	Strong	Physical violence against you or someone you care deeply about	Yes	No
V4	Strong	Sexual violence against you or someone you care deeply about	No	N/A

Continued on next page

Table A1 – continued from previous page

**Code**	**Type**	**Indicator Description**	**Collected in Survey?**	**Has Coding Variants? (Conservative and Liberal)**
V5	Medium	Threat of denunciation to authorities against you or someone you care deeply about	No	N/A
V6	Medium	Emotional/psychological abuse against you or someone you care deeply about	No	N/A
V7	Medium	Threat of harm to your personal or professional reputation	No	N/A
V8	Medium	Threats of violence against you or someone you care deeply about	Yes	No

Notes: This table lists all labor trafficking indicators from Okech et al. (2025). Note that this framework encompasses all forms of human trafficking, and we list all indicators included in Okech et al. (2025) for completeness. However, this paper only measures and studies labor trafficking (not sex trafficking and other forms of human trafficking). "Collected in Survey" notes whether an indicator was collected in our survey. "Type" refers to the strength of the indicator (Extreme, Strong or Medium) used in the classification thresholds shown in Figure 1 in the main text. Note that Okech et al. (2025) only distinguish between Strong and Medium indicators, while we categorize as Extreme those indicators that individually make a worker classified as trafficked (Threshold 1). "Coding Variants" indicates whether the indicator has both conservative and liberal coding in our analysis. Conservative codings use stricter criteria (e.g., "often" confiscates documents), while liberal codings use more inclusive criteria (e.g., "often or sometimes" confiscates documents). See Appendix Section A1 for more details.

Table A2: Balance Tests of Time-Invariant Kiln Characteristics

Balance Variable	Technical+Incentive Arm Mean	Technical+Incentive Arm Std. Dev.	Technical Arm Mean	Technical Arm Std. Dev.	Control Arm Mean	Control Arm Std. Dev.	p-value for Test of Equality I vs. C	p-value for Test of Equality T vs. C	p-value for Test of Equality I vs. T
Owner Experience (Years)	15.602	8.625	17.062	10.247	15.169	9.910	0.849	0.308	0.355
Jashore Pilot Intervention Knowledge	0.361	0.484	0.355	0.482	0.358	0.483	0.825	0.973	0.810
Jashore Owner Interaction	0.455	0.510	0.545	0.510	0.458	0.509	0.798	0.333	0.529
Zigzag Year	2015	4	2014	4	2014	3	0.624	0.360	0.169
Water Adjacent	0.602	0.492	0.625	0.487	0.639	0.483	0.333	0.949	0.313
Bricks Fired (100,000s)	8.079	1.013	7.858	1.192	8.118	1.114	0.485	0.082	0.244
Circuits Completed	6.149	1.512	5.991	1.509	6.119	1.818	0.599	0.711	0.319
Class 1 Production Share (%)	65.193	11.496	67.312	8.380	65.880	10.543	0.946	0.110	0.193
Production Cost BDT (per 1K Bricks)	8869	941	8600	1314	8642	1065	0.077	0.960	0.130
Total Workers	110.133	28.621	111.938	32.697	110.940	35.788	0.903	0.571	0.656
Joint Ownership	0.313	0.467	0.325	0.471	0.398	0.492	0.262	0.264	0.994

All data collected through baseline owner survey. Sample includes 246 kilns with worker surveys and kiln performance monitoring. P-values from regressions with strata fixed effects. See Appendix Figure A2 for more details.

Table A3: Balance Tests of Pre-Existing Worker Characteristics

Balance Variable	Technical+Incentive Arm		Technical Arm		Control Arm		p-value for Test of Equality		
	Mean	Std. Dev.	Mean	Std. Dev.	Mean	Std. Dev.	I vs. C	T vs. C	I vs. T
Age	34.632	9.670	34.597	10.076	34.253	9.947	0.720	0.735	0.997
Years of Education	4.540	3.626	4.700	3.517	4.644	3.444	0.870	0.655	0.772
Male	0.910	0.287	0.929	0.257	0.906	0.292	0.932	0.374	0.468
Literacy	0.018	0.135	0.013	0.113	0.010	0.100	0.536	0.837	0.711
Job as Brick Loader	0.386	0.487	0.377	0.485	0.360	0.481	0.368	0.885	0.471
Job as Molder	0.092	0.290	0.106	0.308	0.098	0.297	0.830	0.499	0.397
Job as Fireman	0.226	0.419	0.263	0.441	0.275	0.447	0.111	0.782	0.071
Job as Brick Unloader	0.296	0.457	0.254	0.436	0.267	0.443	0.301	0.341	0.038
Worked at Same Kiln in Previous Season	0.742	0.438	0.705	0.457	0.682	0.466	0.258	0.855	0.272
Worked at Any Kiln in Previous Season	0.946	0.227	0.948	0.222	0.939	0.240	0.715	0.636	0.912
Worked with Same Sardar in Previous Season	0.747	0.435	0.677	0.468	0.629	0.484	0.008	0.290	0.093
Division of Residence (Khulna)	0.930	0.255	0.931	0.254	0.939	0.240	0.349	0.315	0.869
Division of Residence (Rajshahi)	0.049	0.217	0.060	0.238	0.053	0.224	0.803	0.347	0.485
Division of Residence (Rangpur)	0.000	0.000	0.004	0.066	0.002	0.045	0.266	0.504	0.149
Division of Residence (Dhaka)	0.008	0.090	0.002	0.046	0.002	0.045	0.241	0.960	0.269
Division of Residence (Barisal)	0.012	0.110	0.002	0.046	0.004	0.064	0.123	0.831	0.086
Traveled for Work?	0.462	0.499	0.502	0.501	0.509	0.500	0.944	0.674	0.626
Distance Traveled (km)	45.382	63.948	48.567	67.811	49.141	64.770	0.948	0.696	0.660

All data collected through worker survey. Sample includes 1442 workers from 246 kilns with worker surveys and kiln performance monitoring. P-values from regressions with strata fixed effects and standard errors clustered at kiln level. See Appendix Figure A2 for more details.

Table A4: Summary Statistics

	Mean	Standard Deviation	Minimum	Maximum
**Trafficking and Labor Indicators**				
R1 - Coercive Recruitment*	0.028	0.164	0	1
R2 - Deceptive Recruitment (Services)*	0.064	0.244	0	1
R3 - Deceptive Recruitment (Conditions)	0.057	0.232	0	1
R4 - Paid Recruitment Fees	0.135	0.341	0	1
EP1 - Can't Leave or They Won't Be Paid*	0.043	0.203	0	1
EP2 - High/Increasing Employer Debt*	0.001	0.037	0	1
EP5 - No Compensation for Additional Services (Liberal)	0.101	0.301	0	1
EP5 - No Compensation for Additional Services (Conservative)	0.093	0.290	0	1
EP6 - Withheld Wages/Benefits	0.118	0.323	0	1
EP7 - Recruitment Linked to Debt	0.005	0.070	0	1
EP8 - No Formal Contract	0.706	0.456	0	1
PL1 - Control Over Personal Life*	0.033	0.179	0	1
PL5 - Confiscation of Communication Methods (Liberal)	0.064	0.246	0	1
PL5 - Confiscation of Communication Methods (Conservative)	0.003	0.059	0	1
DC1 - Available Day/Night Without Compensation (Liberal)*	0.019	0.136	0	1
DC1 - Available Day/Night Without Compensation (Conservative)*	0.013	0.114	0	1
DC2 - Hazardous Work Without Protection (Conservative)	0.702	0.457	0	1
DC2 - Hazardous Work Without Protection (Liberal)	1.000	0.000	1.000	1.000
DC4 - Degrading Living Conditions (Conservative)	0.003	0.059	0	1
DC4 - Degrading Living Conditions (Liberal)	0.016	0.125	0	1
FM1 - Confiscation of Identity Documents (Liberal)*	0.078	0.268	0	1
FM1 - Confiscation of Identity Documents (Conservative)*	0.012	0.111	0	1
FM2 - Constant Surveillance of Personal Spaces*	0.037	0.188	0	1
FM3 - No Freedom of Movement/Communication*	0.050	0.218	0	1
FM4 - Limited Freedom of Movement/Communication	0.424	0.494	0	1
DD1 - Debt Imposed Without Consent*	0.005	0.070	0	1
DD4 - Unable to Refuse Services	0.078	0.268	0	1
V1 - Witnessed Physical Violence*	0.070	0.255	0	1
V3 - Physical Violence Against You/Loved Ones*	0.019	0.138	0	1
V8 - Threats of Violence	0.053	0.225	0	1
Worker is Trafficked (Conservative)	0.186	0.389	0	1
Worker is Trafficked (Liberal)	0.241	0.428	0	1
Weighted Count of Indicators (Conservative)	1.911	1.244	0.000	8.667
Weighted Count of Indicators (Liberal)	2.234	1.322	0.667	9.333
**Child Labor**				
Child Labor Exists	0.325	0.468	0	1
Child Labor Exists for Children Under 14	0.058	0.233	0	1
Share of Workers Under 18 in Respondent's Team	0.043	0.080	0.000	0.500
Share of Workers Under 14 in Respondent's Team	0.006	0.028	0.000	0.400
**Amenities to Workers**				
Supplies Cooking Fuel	0.607	0.488	0	1
Separate Toilet Facilities for Men and Women	0.162	0.369	0	1
Piped Water Toilets	0.047	0.212	0	1
Privacy in Dwelling	0.501	0.500	0	1
Shed for Rest During Work	0.477	0.500	0	1
No Benefits from Sardar	0.613	0.487	0	1
Received Bonus from Sardar	0.036	0.187	0	1
Received Wage Increase from Sardar	0.010	0.101	0	1
Received Extra Meals from Sardar	0.147	0.354	0	1
Received Mobile Phone from Sardar	0.001	0.026	0	1
Received Travel Allowance from Sardar	0.022	0.147	0	1
Received Clothing from Sardar	0.012	0.111	0	1
Received Bakhshish from Sardar	0.019	0.136	0	1
**Worker-Reported Wages and Health**				
Weekly Wages (Worker-Reported)	3784	1110	0	9100
Work-Related Injury	0.287	0.453	0	1
Physical Health Symptoms	0.249	0.433	0	1
Anxiety Score (GAD-7)	4.282	3.320	0.000	21.000
Depression Score (PHQ-9)	4.607	3.334	0.000	27.000
Respiratory Symptoms	0.125	0.331	0	1
Respiratory Problems	0.015	0.120	0	1
Constant Cough	0.061	0.239	0	1
Difficulty Breathing	0.049	0.215	0	1
Wheezing or Panting or Asthma	0.036	0.187	0	1
**Owner-Reported Wages**				
Molder Wages (Owner-Reported)	1024	123	600	1200
Brick Loader Wages (Owner-Reported)	313	42	200	400
Brick Unloader Wages (Owner-Reported)	207	32	160	310
Firemen Wages (Owner-Reported)	1083610	147867	675000	1400000
**Kiln-Level Outcomes**				
Trafficked Worker at Kiln (Conservative)	0.496	0.501	0	1
Trafficked Worker at Kiln (Liberal)	0.602	0.491	0	1
Weighted Count of Unique Indicators at Kiln (Conservative)	4.198	2.304	0.667	11.667
Weighted Count of Unique Indicators at Kiln (Liberal)	4.692	2.554	1.333	13.000
Weighted Count of Target Indicators at Kiln (Conservative)	0.706	0.321	0.000	2.333
Weighted Count of Target Indicators at Kiln (Liberal)	0.798	0.340	0.667	2.333
Child Labor Exists at Kiln	0.714	0.453	0	1
Child Labor Exists at Kiln for Children Under 14	0.188	0.391	0	1
N: 1442 individuals; 246 kilns; 1400 owner wage observations.				

* Strong Indicator

Table A5: ITT Estimates for Labor Trafficking by Study Arm (Liberal Coding)

	Weighted Count of Indicators				Trafficking Prevalence	
Treatment Arm	(1)	(2)	(3)	(4)	(5)	(6)
Technical Only	0.093	0.001	0.118	-0.031	0.015	-0.033
	(0.137)	(0.016)	(0.376)	(0.056)	(0.040)	(0.075)
Technical+Incentive	0.019	-0.011	0.280	-0.019	-0.033	-0.112
	(0.125)	(0.012)	(0.402)	(0.053)	(0.038)	(0.074)
Control Mean	2.219	0.699	4.598	0.807	0.253	0.651
Kiln or Worker Level?	Worker	Worker	Kiln	Kiln	Worker	Kiln
All or Target?	All	Target	All	Target	-	-
Observations	1442	1442	246	246	1442	246

Standard errors in parentheses. All standard errors are clustered at the kiln level for worker level analyses and are heteroskedasticity-robust for kiln level analyses. Columns 1-4: The dependent variable is a weighted count of the trafficking indicators observed; medium indicators receive 2/3rds the weight of strong and extreme indicators. Estimates are OLS estimates generated from regressing the weighted count of trafficking indicators on treatment arm dummy variables, with fixed effects for randomization strata. Columns 1-2 show regression results for the worker-level weighted count of indicators. Columns 3-4 show results for the weighted count of unique indicators in each kiln. Target indicators use the same weighting and include indicators that are most closely related to the incentives: DC1 (Made to be available day and night without adequate compensation outside of the scope of the contract); DC2 (Made to complete hazardous and/or arduous services without proper protective gear); and DC4 (Made to live in degrading conditions). Columns 5-6: Estimates are OLS estimates generated from regressing an indicator for trafficking on treatment arm dummy variables, with fixed effects for randomization strata. To generate kiln-level data, we code kilns as having trafficking if any of the workers interviewed at that kiln met the definition for trafficking.

* $p < 0.1$, ** $p < 0.05$, *** $p < 0.01$

Table A6: Adoption and Impact of Technical Intervention on Efficiency and Productivity

	Adopted Technical Intervention	Specific Energy Consumption (MJ/kg fired brick)	Specific Fuel Consumption (tons/100,000 bricks)	Fuel Spending (BDT/Brick)	Class 1 Bricks (% of total production)
Technical Only	0.45***	-0.10***	-1.70***	-0.41***	0.06***
	(0.06)	(0.03)	(0.41)	(0.10)	(0.01)
Technical+Incentive	0.44***	-0.12***	-1.91***	-0.31***	0.06***
	(0.06)	(0.03)	(0.44)	(0.09)	(0.01)
Observations	276	276	276	276	276
Control Mean	0.20	1.07	16.07	3.74	0.78

Heteroskedasticity-robust standard errors in parentheses. Estimates are OLS estimates generated from regressing each outcome on treatment arm dummy variables, with fixed effects for randomization strata. Adoption (column 1) is a binary for whether a kiln adopted the improved brick stacking and fuel feeding practices or not. Specific Energy Consumption (column 2) measures megajoules per kilogram of fired brick. Specific Fuel Consumption (column 3) measures tons of coal per 100,000 bricks produced. Fuel Spending (column 4) is measured in Bangladeshi Taka (BDT) per brick. Class 1 Bricks (column 5) represents the percentage of total production meeting Class 1 quality standards.

* $p < 0.1$, ** $p < 0.05$, *** $p < 0.01$

Table A7: Impact of Technical Intervention on Owner-Reported Costs

	Soil Cost (BDT per 1000 bricks)	Molding Cost (BDT per 1000 bricks)	Coal Preparation Cost (BDT per season)	Brick Loading Cost (BDT per 1000 bricks)	Brick Unloading Cost (BDT per 1000 bricks)	Firemen Cost (BDT per season)	Total Production Cost (BDT per 1000 bricks)
Technical Only	1.59	6.58	13239.60	5.85	3.35	18452.90	-112.10
	(14.89)	(17.36)	(8852.78)	(5.81)	(4.63)	(22541.56)	(93.07)
Technical+Incentive	22.62	2.30	2502.05	6.27	4.09	22234.53	2.64
	(15.36)	(16.59)	(8255.78)	(5.90)	(4.54)	(22297.62)	(95.72)
Observations	276	276	276	276	276	276	276
Control Mean	902.30	1026.79	214463.91	307.52	204.54	1.07e+06	9234.78

Heteroskedasticity-robust standard errors in parentheses. Estimates are OLS estimates generated from regressing each cost measure on treatment arm dummy variables, with fixed effects for randomization strata. All costs are owner-reported and measured in Bangladeshi Taka (BDT). Columns 1, 2, 4, 5, and 7 show costs per 1,000 bricks. Columns 3 and 6 show total seasonal costs for coal preparation and firemen, respectively. Total Production Cost (column 7) represents the owner's estimate of total cost per 1,000 bricks, including all inputs.

* $p < 0.1$, ** $p < 0.05$, *** $p < 0.01$

Section A.6: Worker Survey Questionnaire

The full worker survey instrument is reproduced on the following pages. The survey was administered in Bangla, but we also include the original English, the question identifiers (QSL), and any applicable skip logic.

BRICK KILN: WORKER SURVEY

Module 0: Identifier

QSL	QUES: ENG	QUES: BNG	Skip Note
**I0**	Who is the respondent?<br>1= Worker<br>2= Sardar	উত্তরদাতা কে?<br>1= শ্রমিক<br>2= সর্দার	
date	Date	তারিখ	
consent	Was consent granted?<br><br>1=Yes; 0=No	সম্মতি দেওয়া হয়েছিল?<br><br>1=হ্যাঁ; 0=না	If no, Skip to C1
I1	Name of worker	উত্তরদাতা নাম	
I2	Phone number	ফোন নম্বর	
I3	Age (in years)	বয়স (বছরে)	
I4	Sex<br><br>1=Male; 2=Female	লিঙ্গ<br><br>1=পুরুষ; 2=মহিলা	Skip to I5, If I0=2
5	How many years of education do you have? *(Years of education completed since class 1)*	আপনি কোন শ্রেণী পর্যন্ত পড়াশুনা করেছেন।<br><br>*(ক্লাস ১ থেকে শিক্ষার কত বছর শেষ হয়েছে)*	If I5=0, then go to I6, else go to I7
I6	Can you read and write?<br><br>1=Yes; 0=No	আপনি পড়তে এবং লিখতে পারেন?<br><br>1=হ্যাঁ; 0=না	
I7	Kiln ID (5 digits)	ইট ভাটার আইডি	
I8	District (select 1 from)<br>1=Chuadanga<br>2=Jhenaidah<br>3=Khushtia<br>4=Narail<br>5=Magura<br>6=Khulna<br>7=Jashore	জেলা (নিচের ১টি জেলা নির্বাচন করুন)<br>1=চুয়াডাঙ্গা<br>2=ঝিনাইদহ<br>3=কুষ্টিয়া<br>4=নড়াইল<br>5=মাগুরা<br>6=খুলনা<br>7=যশোর	Skip to I10, If I0=2
I9	What is the name of your current sardar?	আপনার বর্তমান সর্দারের নাম কী	Skip to I11, If I0=1
I10	What is the name of your current manager/kiln owner?	আপনার বর্তমান মালিকের নাম কী	
I11	How long have you worked with him? (months)	আপনি কতদিন যাবত বর্তমান সর্দারের/মালিকের সাথে কাজ করেছেন? (মাসে লিখুন)	
			**Skip I11a-I11e, If I0=1**
I11a	How did you learn about him and his brick kilns?<br>1= Social network who work in kilns<br>2= News paper<br>77= Others	আপনি কিভাবে তার সম্পর্কে এবং তার ইট ভাটাগুলি সম্পর্কে জানলে?<br>1 = ইট ভাটাগুলিতে কাজ করা সামাজিক নেটওয়ার্ক<br>2= খবরের কাগজ<br>77= অন্যান্য	
I11b	Do you have a formal contract with him?<br>1= Yes, written indefinite contract	আপনার তার সাথে কোন লিখিত চুক্তি আছে কি?<br><br>1 = হ্যাঁ, অনির্দিষ্ট চুক্তি লেখা আছে।<br>2 = হ্যাঁ, নির্দিষ্ট সময়কাল বা প্রকল্প ভিত্তিক চুক্তি আছে।	

QSL	QUES: ENG	QUES: BNG	Skip Note
	2 = Yes, fixed-term or project-based contract<br>3 = Oral contract<br>4 = No contract at all	3 = মৌখিক চুক্তি আছে।<br>4 = কোন চুক্তি নেই।	
I11c	Do you get an advance from the owner to confirm the contract?<br><br>1= Yes; 0= No	আপনি কি চুক্তি নিশ্চিত করার জন্য মালিকের কাছ থেকে অগ্রিম পান?<br><br>1=হ্যাঁ; 0=না	
I11d	What is the payment of the contract based on? (Select all that applies)<br>1= Number of bricks<br>2= Quality of bricks<br>3= Number of workers<br>77= Other	চুক্তি প্রদানের অর্থ কিসের ভিত্তিতে হয়? (প্রযোজ্য সমস্ত নির্বাচন করুন)<br>1= ইটের সংখ্যা<br>2 = ইটের গুণমান<br>3 = শ্রমিকের সংখ্যা<br>77= অন্যান্য	
I11e	How many other kilns do you also work for? [Insert 0 if this is the only one]	আপনি আরও কতগুলি ভাটায় কাজ করেন? [একমাত্র এটি হলে 0 বসান]	
I12a	How many workers report to you?	কতজন কর্মী আপনাকে রিপোর্ট করে?	Skip I12a, if I0=1
I12b	Do you have a formal contract with your workers?<br>1= Yes, written indefinite contract<br>2 = Yes, fixed-term or project-based contract<br>3 = Oral contract<br>4 = No contract at all	আপনার কি কর্মীদের সাথে কোন নির্দিষ্ট চুক্তি আছে?<br><br>1 = হ্যাঁ, লিখিত অনির্দিষ্ট চুক্তি<br>2 = হ্যাঁ, নির্দিষ্ট-মেয়াদী বা প্রকল্প ভিত্তিক চুক্তি<br>3 = মৌখিক চুক্তি<br>4 = মোটেও কোন চুক্তি নেই	Skip I12b, if I0=1
I13	What is your job?<br>1= Moulder<br>2= Brick loaders<br>3= Fireman<br>4= Brick unloader<br>5= Coal crusher	আপনার কাজ কি?<br>1 =ইটের ছাঁচকার<br>2 = ইট বোঝাই মিস্ত্রি<br>3 = পোড়াই মিস্ত্রি<br>4 = ইট আনলোডার/ খালাসি<br>5 = কয়লা ফাইনকারি	Skip I13, if I0=2
I14	What other job did you do this season? (select all that applies)<br><br>0= Not allowed to do anything else<br>1= Moulder<br>2= Brick loaders<br>3= Fireman<br>4= Brick unloader<br>5= Coal crusher<br>6= Clay preparation<br>7= Loading bricks on trucks<br>77=Other (Specify)	আপনি এই মৌসুমে অন্য কোন কাজ করতে করেছেন? (প্রযোজ্য সমস্ত নির্বাচন করুন)<br><br>0= অন্য কোন কাজ করার অনুমতি নাই<br>1= কাটামিস্ত্রি<br>2 = বোঝাইমিস্ত্রি<br>3 = পোড়াইমিস্ত্রি<br>4 = ইট আনলোডার<br>5 = কয়লা ফাইনকারি মিস্ত্রি<br>6 = কাচা মাটি প্রস্তুতিতে<br>7 = ট্রাকগুলিতে ইট ভর্তি করতে<br>77=অন্যান্য	Skip I14, if I0=2
I14a	Have you worked in a brick kiln before?<br><br>1=Yes; 0=No	আপনি কি ভাটায় আগে কখনও কাজ করেছেন?<br><br>1=হ্যাঁ; 0=না	I0=1 and I14a=0, go to I18, else go to I15
I15	Have you worked at this kiln before?<br>0=No<br>1= Yes, in the last firing season	আপনি কি এই ভাটায় আগে কাজ করেছেন?<br>0 = না<br>1 = হ্যাঁ, গত ফায়ারিং মৌসুমে	I0=1 and I15=0, go to I16, else go to I15a
I15a	Did you work with the same owner during that time?	আপনি কি তখন একই মালিকের সাথে কাজ করেছিলেন?	Skip I15a, if I0=1

QSL	QUES: ENG	QUES: BNG	Skip Note
	1=Yes; 0=No	1=হ্যাঁ; 0=না	
I16	Where was the other kiln located?<br>1= Same district<br>2= Another district but same division<br>3= Another division<br>99= Don't remember	অন্য ভাটাটি কোথায় ছিল?<br><br>1 = একই জেলা<br>2 = অন্য জেলায় কিন্তু একই বিভাগ<br>3 = অন্য বিভাগে<br>99 = মনে নেই	Skip I16, if I0=2
I17	Did you work with the same sardar during that time?<br><br>1=Yes; 0=No	আপনি কি তখন একই সর্দারের সাথে কাজ করেছিলেন?<br><br>1=হ্যাঁ; 0=না	Skip I16, if I0=2
I18	What do you do for the rest of the year if not working at a brick kiln?<br><br>1= Farmer<br>2= Agri-labor<br>3= Construction labor<br>4= Mason (*Rajmistri*)<br>5= Non-agri labor<br>6= Van/Rickshaw puller<br>6= Garment's worker<br>77=Other (Specify)	আপনার যখন ইটের ভাটায় কাজ থাকে না, তখন অন্য কোন কাজ করেন?<br><br>1= কৃষক<br>2= কৃষি শ্রমিক<br>3= নির্মাণকর্মী<br>4= রাজমিস্ত্রী<br>5= শ্রমিকঃ কৃষি শ্রমিক ছাড়া<br>6= ভ্যান/রিকশা চালক<br>7= গার্মেন্টস কর্মী<br>77= অন্যান্য (নির্দিষ্ট করুন)	

Module 1: Migration

QSL	QUES: ENG	QUES: BNG	Skip Note
M2	Now I will ask you about where you usually live. What is the division?<br>1=Dhaka<br>2=Khulna<br>3=Rajshahi<br>4=Rangpur<br>5=Barisal<br>6=Chittagong<br>7=Sylhet<br>8=Mymensingh	আপনি সাধারণতঃ কোথায় বাস করেন। কোন বিভাগ?<br>1 = ঢাকা<br>2 = খুলনা<br>3 = রাজশাহী<br>4 = রংপুর<br>5 = বরিশাল<br>6 = চট্টগ্রাম<br>7 = সিলেট<br>8 = ময়মনসিংহ	
M3	What is the District?	কোন জেলা?	
M4	What is the Upazila?	কোন উপজেলা?	
M5	Did you travel away from your home to come to work at this job?<br><br>1 = Yes, I traveled<br>2 = No, I did not travel away from home	আপনি কি এই কাজ করার জন্য আপনার বাড়ি থেকে অনেক দূরে এসেছেন?<br><br>1 = হ্যাঁ, আমি বাড়ি থেকে অনেক দূরে এসেছি<br>2 = না, আমি বাড়ি থেকে বেশী দূরত্বে আসিনি	If M5=2, then skip to M7
M5a	What is the distance between your house and the kiln? (in km)	আপনার বাড়ি এবং ভাটার মধ্যে দূরত্ব কত? (কিঃ মিঃ এ লিখুন)	
M6	Do you intend to go back to your permanent residence after the work is over/end of the firing season?<br><br>1=Yes; 0=No	আপনি কি কাজ শেষ হওয়ার পরে (এই পোড়াই মৌসুম শেষে) আপনার স্থায়ী ঠিকানায়/বাড়িতে ফিরে যেতে চান?<br><br>1=হ্যাঁ; 0=না	
	Skip M7-M12, if I0=2		
M7	Did your family travel /work with you? (multiple answer possible)<br><br>1 = No, I came alone<br>2 = Yes, I came with my spouse<br>3 = Yes, I came with my children<br>4 = Yes, I came with other family members	আপনার পরিবার কি আপনার সাথে এসেছে /কাজ করে? (একাধিক উত্তর প্রযোজ্য)<br><br>1 = না, আমি একা এসেছি<br>2 = হ্যাঁ, আমি আমার স্বামী/স্ত্রী নিয়ে এসেছি<br>3 = হ্যাঁ, আমি আমার বাচ্চাদের সাথে নিয়ে এসেছি<br>4 = হ্যাঁ, আমি পরিবারের অন্যান্য সদস্যদের সাথে এসেছি	If M7=3, then go to M8; OR If M7=1, then go to M13, M7=4, then go to M13
M8	If M7=3, How many children (<18 years) came with you?	যদি M7=3 হয়, তবে কতজন ছেলেমেয়ে (১৮ বছর এর কম) আপনার সাথে এসেছিল?	
M9	Does your spouse also work here in the kiln?<br>1= Yes, my spouse always works at the kiln with me on the same team<br>2= Yes, my spouse always works at the kiln on another team<br>3= Yes, my spouse sometimes works at the kiln with me on the same team<br>4= Yes, my spouse sometimes works at the kiln on another team<br>5= Yes, my spouse often works at the same kiln on another team<br>6= My spouse does not work in the kilns with me, but does other work for kiln workers (such as cooking or cleaning)	আপনার স্বামী/ স্ত্রী কি এখানে ভাটায় কাজ করে?<br><br>1=হ্যাঁ, আমার স্বামী/স্ত্রী সবসময় একই দলে আমার সাথে ভাটায় কাজ করে<br>2= হ্যাঁ, আমার স্বামী/স্ত্রী সবসময় অন্য দলের সাথে ভাটায় কাজ করে<br>3=হ্যাঁ, আমার স্বামী/স্ত্রী মাঝে মাঝে একই দলে আমার সাথে ভাটায় কাজ করে<br>4= হ্যাঁ, আমার স্বামী/স্ত্রী মাঝে মাঝে অন্য দলের সাথে ভাটায় কাজ করে<br>5= হ্যাঁ, আমার স্বামী/স্ত্রী প্রায়ই একই ভাটাতে অন্য দলে কাজ করে	If M7=3, then go to M10, else go to M11

QSL	QUES: ENG	QUES: BNG	Skip Note
	7= My spouse does not work in the kilns with me and also does not work for kiln workers	6= আমার স্বামী/স্ত্রী আমার সাথে ভাটায় কাজ করে না, তবে অন্যান্য কর্মীদের জন্য অন্য কাজ করে (যেমন রান্না বা পরিষ্কার করা)<br>7= আমার স্বামী/স্ত্রী আমার সাথে ভাটায় কাজ করে না এবং ভাটার কর্মীদের জন্যও কাজ করে না	
M10	Do one or more of your children (<18 years) also work here in the kiln?<br><br>1= Yes, my children always work at the kiln with me on the same team<br>2= Yes, my children always work at the kiln on another team<br>3= My children sometimes work in the kilns with me on the same team<br>4= My children sometimes work in the kilns with me on another team<br>5= My children often work in the same kiln with another team<br>6= My children do not work in the kilns with me, but they do other work for kiln workers (such as cooking or cleaning)<br>7= My children do not work in the kilns with me and also do not work for kiln workers	একই ভাটায় আপনার কোন ছেলেমেয়ে (১৮ বছর এর কম) কি কাজ করে?<br><br>1=হ্যাঁ, আমার ছেলে/মেয়েরা সবসময় একই দলে আমার সাথে ভাটায় কাজ করে<br>2= হ্যাঁ, আমার ছেলে/মেয়েরা সবসময় অন্য দলের সাথে ভাটায় কাজ করে<br>3= আমার ছেলে/মেয়েরা মাঝে মাঝে আমার সাথে একই দলে ভাটায় কাজ করে<br>4= আমার ছেলে/মেয়েরা মাঝে মাঝে অন্য দলের সাথে একই ভাটাতে কাজ করে<br>5= আমার ছেলে/মেয়েরা প্রায়ই অন্য দলের সাথে একই ভাটাতে কাজ করে<br>6= আমার ছেলে/মেয়েরা আমার সাথে ভাটায় কাজ করে না, তবে তারা ভাটা শ্রমিকদের জন্য অন্যান্য কাজ করে (যেমন রান্না বা পরিষ্কার করা)<br>7= আমার ছেলে/মেয়েরা আমার সাথে ভাটায় কাজ করে না এবং ভাটার কর্মীদের জন্যও কাজ করে না	
M11	How many of your children (<18 years) work here?	আপনার কতজন ছেলে/মেয়ে (১৮ বছর এর কম) এখানে কাজ করে?	If M11=0, then go to M13
M12	What is their age?	তাদের বয়স কত?	Loop question; for every child in M11
			**Skip M12a & M12b, If I0=1**
M12a	Where do you usually recruit your workers from?<br>1= From my district<br>2= From both my and other districts	আপনি সাধারণত আপনার কর্মীদের কোথা থেকে নিয়োগ করেন?<br>1= আমার জেলা থেকে<br>2= আমার এবং অন্য জেলা উভয় থেকে নিয়োগ করি	
M12b	Is it generally difficult to recruit brick kiln workers?<br>1 = Yes difficult, and i need to try to persuade workers to join my team<br>2 = Yes difficult, but I don't need to do anything to persuade workers to join<br>3 = No, not difficult to find workers	সাধারণত ইট ভাটায় শ্রমিক নিয়োগ করা কি কঠিন?<br><br>1= হ্যাঁ কঠিন, এবং আমার দলে যোগদানের জন্য কর্মীদের রাজি করার চেষ্টা করতে হবে<br>2= হ্যাঁ কঠিন, কিন্তু কর্মীদের যোগদান করতে রাজি করার জন্য আমার কিছু করার দরকার নেই<br>3= না, শ্রমিক পাওয়া কঠিন নয়	
			If M8>0, then answer the ques M18-M19, else go to next module**. (Skip M18 & M19, If I0=2)**
M18	Does the owner do anything to facilitate the education of the children of workers?<br>1=Yes; 0=No	শ্রমিকদের বাচ্চাদের শিক্ষার সুবিধার্থে মালিক কি কিছু করেন?<br><br>1=হ্যাঁ; 0=না	
M19	If yes, what is provided?<br><br>1= school fees<br>2= materials (e.g., bags, books, pens, pencils)<br>3= school at the kiln	যদি হ্যাঁ, তাহলে কি দেয়া হয়?<br><br>1= স্কুলের বেতন<br>2= শিক্ষার উপকরণ (যেমনঃ ব্যাগ, বই, কলম, পেন্সিল)<br>3= ভাটায় অবস্থিত স্কুল	

Module 2: Work Conditions

QSL	QUES: ENG	QUES: BNG	Skip Note
			**Skip W6 to W7, If I0=2**
W6	About how many people do you work with at this job?	আপনি দলে কতজন লোকের সাথে এই কাজ করেন?	(W6-W6c only for those groups they work)
W6a	About how many people (in your team) working here appear under the age of 14?	এখানে আপনার দলে কাজ করা কত লোক 14 বছরের কম বয়সের বলে মনে হয়?	
W6b	About how many people (in your team) working here appear to be between 14 and 18 years old?	এখানে আপনার দলে কাজ করা কত লোক 14 থেকে 18 বছরের মধ্যে রয়েছে বলে মনে হয়?	
W6c	About how many people (in your team) working here appear older than 18 years old?	এখানে আপনার দলে কাজ করা কতজন লোক 18 বছরের বেশি বয়সের চেয়ে বেশি বলে?	
W7	At this kiln, does the owner provide cooking fuel?<br><br>1=Yes; 0=No	এই ভাটায়, মালিক কি রান্নার জ্বালানি সরবরাহ করে?<br><br>1=হ্যাঁ; 0=না	
W8	What is the source of drinking water?<br><br>1= Tube-Well<br>2= Tap Water<br>3= Well Water<br>77= Others	খাবার পানির উৎস কি?<br><br>1= টিউব-ওয়েল<br>2= ট্যাপ এর পানি<br>3= কূপের পানি<br>77= অন্যান্য	
W9	Is there any toilet facility in the brick kiln site for workers?<br><br>1=Yes; 0=No	শ্রমিকদের জন্য ইট ভাটার এলাকায় কোনও টয়লেট সুবিধা রয়েছে?<br><br>1=হ্যাঁ; 0=না	If W9=1 then go to W11, else W10
W10	Do you have access to a toilet facility if you want to use one?<br><br>1= Yes, nearby public toilet<br>2= Yes, nearby private toilet<br>0= No	আপনার যদি টয়লেট ব্যবহার প্রয়োজন হয়, তবে কি কোন টয়লেট সুবিধা রয়েছে?<br><br>1= হ্যাঁ, কাছাকাছি পাবলিক টয়লেট<br>2= হ্যাঁ, কাছাকাছি নিজেদের (ব্যক্তিগত) টয়লেট<br>0= না	If W9=0 then go to W13, else go to W11
W11	If there is a toilet facility (yes to the above question):<br><br>Are separate toilets for male and female workers provided? ----1=Yes; 0=No	যদি কোন টয়লেট সুবিধা থাকে (উপরের প্রশ্নের হ্যাঁ):<br><br>পুরুষ ও মহিলা শ্রমিকদের জন্য পৃথক টয়লেট আছে কি? ---1=হ্যাঁ; 0=না	
W12	Whether piped water supply is provided in the toilets?<br><br>1=Yes; 0=No	টয়লেটে কি পাইপের মাধ্যমে পানির ব্যবস্থা আছে?<br><br>1=হ্যাঁ; 0=না	
W13	Is there a shed for you to rest in between work?<br>1=Yes; 0=No	কাজের মধ্যে বিশ্রাম নেওয়ার জন্য আপনার জন্য কি কোনও ছাউনি/চালা আছে?<br>1=হ্যাঁ; 0=না	If W13=0, then go to W14, else go to W15
W14	if W13=0, Is there a shed for other workers?<br>1=Yes; 0=No	যদি W13=0 হয়, তবে অন্যান্য শ্রমিকদের বিশ্রামের জন্য কি ছাউনি/চালা আছে?<br>1=হ্যাঁ; 0=না	
W15	Is there housing provided for you on or near the site?<br>1=Yes; 0=No	আপনার জন্য কি ভাটার বা তার কাছাকাছি আবাসন ব্যবস্থা করা হয়েছে?<br>1=হ্যাঁ; 0=না	If W15=0, then go to

QSL	QUES: ENG	QUES: BNG	Skip Note
			W16, else go to W17
W16	if W15=0, is there housing provided for other workers?<br>1=Yes; 0=No	যদি W15=0 হয়, তবে অন্য শ্রমিকদের জন্য আবাসন ব্যবস্থা আছে কি?<br>1=হ্যাঁ; 0=না	If W15=0 and W16=0, then skip to W18.
W17	Is electricity available in the housing? (skip W17 if there is no accommodation & no electricity at the kiln)<br>1=Yes; 0=No	আবাসন গুলিতে কি বিদ্যুৎ পাওয়া যায়? (যদি কোন বাসস্থান না থাকে এবং ভাটায় বিদ্যুৎ না থাকে তবে W17 এড়িয়ে যান)<br>1=হ্যাঁ; 0=না	If W15=0 then go to W18
W17a	Do you live onsite?<br>1= Yes<br>2= No, I live nearby <30 minutes away<br>3= No, I travel more than 30 minutes every day to come to the kiln	আপনি কি ভাটাতে থাকেন?<br>1= হ্যাঁ<br>2= না, আমি ভাটার কাছাকাছি ৩০ মিনিট এর কম দূরত্বে থাকি<br>3= না, আমার ভাটাতে আসতে প্রতিদিন ৩০ মিনিটেরও বেশি সময় লাগে	If W15=0, then go to W18
W17b	Sometimes people live in housing provided by their employer and are not allowed to live elsewhere. Were you forced to live in housing provided by your employer?<br>1=Yes; 0=No	কখনও কখনও ভাটার শ্রমিকরা তাদের মালিক দ্বারা সরবরাহিত আবাসনগুলিতে বাস করে এবং অন্য কোথাও থাকার অনুমতি পায় না। আপনি কি আপনার মালিক দ্বারা সরবরাহিত আবাসনগুলিতে থাকতে বাধ্য হয়েছিল?<br>1=হ্যাঁ; 0=না	
W17c	Do you have privacy where you live (e.g. have a door, curtains)?<br>1=Yes; 0=No	আপনি যেখানে থাকেন সেখানে কি আপনার গোপনীয়তা আছে (যেমন: একটি দরজা; পর্দা আছে)?<br>1=হ্যাঁ; 0=না	
W18	***(Now think about other brick kilns you know about around here.)*** Out of 10 kilns in the area, how many do you think have workers in the following age groups? (Don't know=99)<br>1. [___] <14 years<br>2. [___] 14-17 years<br>3. [___] 18-24 years	***(এখন আপনি জানেন এমন অন্যান্য ইট ভাটা সম্পর্কে চিন্তা করুন।)*** এই এলাকার 10টি ভাটার মধ্যে কতটি ভাটায় নিম্নলিখিত বছর বয়সী শ্রমিক আছে? (জানিনা=99)<br>1. [___] < ১৪ বছর<br>2. [___] ১৪-১৭ বছর<br>3. [___] ১৮-২৪ বছর	Skip W18, If I0=2
W19	Did you provide/Were you provided any of the following for your work? (multiple answer)<br>1. Gloves [___]<br>2. Helmet [___]<br>3. Safety shoes/ boots [___]<br>4. Goggles [___]<br>5. Face mask [___]<br>6. Nothing provides [___]<br>77. Other [___]	আপনি কি আপনার কাজের জন্য নিম্নলিখিত কোনটি প্রদান করেছেন/আপনাকে কি প্রদান করা হয়েছে? (একাধিক উত্তর গ্রহণযোগ্য)<br>1. দস্তানা [___]<br>2. হেলমেট [___]<br>3. সুরক্ষা জুতা/ বুট [___]<br>4. সুরক্ষা চশমা [___]<br>5. মাস্ক [___]<br>6. কিছুই দেয়না [___]<br>77. অন্যান্য [___]	

Module: Incentives/compensation

QSL	QUES: ENG	QUES: BNG	Skip Note
	Enumerator instruction: *Regular wages/salary means the normal pay and does not include any bonus or other benefit.*	***সাক্ষাৎকার গ্রহনকারীর নোট:*** *নিয়মিত মজুরি/বেতন মানে সাধারণ বেতন এবং কোনও বোনাস বা অন্য সুবিধা অন্তর্ভুক্ত না করে ।*	
IN0	How much wage did you expect at the beginning of this job?<br><br>1= Per unit of count (BDT per 1000 bricks)	এই কাজের শুরুতে আপনি কিভাবে মজুরি পাওয়ার আশা করেন?<br><br>1= প্রতি ইউনিট গণনা করে (টাকা প্রতি 1000 ইট)	Skip IN0, If I0=2

QSL	QUES: ENG	QUES: BNG	Skip Note
	2= Per unit of weight<br>4= Daily<br>5= Weekly<br>6= Monthly<br>7= Duration of the firing season<br>***(ENUMERATOR record units)***	2= ওজন প্রতি ইউনিট<br>3= প্রতিদিন<br>4= সাপ্তাহিক<br>5= মাসিক<br>6= ফায়ারিং মৌসুম সময়কাল<br>***(সাক্ষাৎকার গ্রহনকারীর নোট:*** ইউনিট নির্ধারণ করুন)	
IN1	How often did you expect to be paid, after the advance in the beginning of the season?<br><br>1= Weekly<br>2= Monthly<br>3= At the end of contract<br>4= Never<br>77= Others (Specify)	মৌসুমের শুরুতে অগ্রিম পাবার পর আপনি কিভাবে মজুরি পাবেন বলে আশা করেছিলেন?<br><br>1= সাপ্তাহিক<br>2= মাসিক<br>3= চুক্তি শেষে<br>4= কখনও না<br>77= অন্যান্য (উল্লেখ্য করুন)	Skip IN1, If I0=1
IN2	How often did you actually receive wages from sardar?<br>1= Daily<br>2= Weekly<br>3= Monthly<br>4= At the end of contract<br>5= Never<br>77= Others (Specify)	আপনি আসলে কিভাবে সর্দারের কাছ থেকে মজুরি পেয়েছিলেন?<br>1= প্রতিদিন<br>2= সাপ্তাহিক<br>3= মাসিক<br>4= চুক্তি শেষে<br>5= কখনও না<br>77= অন্যান্য (উল্লেখ্য করুন)	Skip IN2, If I0=2
			**Skip IN2a-IN2c, If I0=1**
IN2a	How often did you actually receive wages from owner?<br>1= Weekly<br>2= Monthly<br>3= At the end of contract<br>4= Never<br>77= Others (Specify)	আপনি আসলে কিভাবে মালিকের কাছ থেকে মজুরি পেয়েছিলেন?<br>1= সাপ্তাহিক<br>2= মাসিক<br>3= চুক্তি শেষে<br>4= কখনও না<br>77= অন্যান্য (উল্লেখ্য করুন)	
IN2b	For many reasons there may be delays in paying your workers. In this brick season, did you ever have to delay payment to wokers?<br>1= Often<br>2= Sometimes<br>3= Never	অনেক কারণে, আপনার কর্মীদের বেতন দিতে বিলম্ব হতে পারে। এই মৌসুমে, আপনাকে কি কখনও শ্রমিকদের অর্থ প্রদানে বিলম্ব করতে হয়েছে?<br>1= প্রায়ই<br>2= মাঝে মাঝে<br>3 = কখনই না	
IN2c	What was the reason for the delay?<br>1 = Owner did not pay me on time<br>2 = Owner paid me less than I expected<br>3 = Other expenses came up<br>4 = Workers did not finish their job on time<br>5 = Other	বিলম্বের কারণ কী ছিল?<br>1 = মালিক আমাকে সময়মতো টাকা দেননি<br>2 = মালিক আমাকে আমার প্রত্যাশার চেয়ে কম অর্থ প্রদান করেছেন<br>3 = অন্যান্য খরচ উঠে এসেছে<br>4 = শ্রমিকরা সময়মতো তাদের কাজ শেষ করেনি<br>5 = অন্যান্য	Skip IN2c, if IN2b=3
IN3	Did you receive the wages you expected?<br><br>1 = Yes, the same wages or more<br>2 = No, less wages<br>3 = No wages at all	আপনি কি প্রত্যাশিত মজুরি গ্রহণ করেছেন?<br><br>1 = হ্যাঁ, একই মজুরি বা আরও কিছু বেশি<br>2 = না, কম মজুরি<br>3 = মোটেও মজুরি নেই	Skip IN3, If I0=2
IN4	How much do you earn per week (on average) from working in the kiln?	সপ্তাহে (গড়ে) আপনি ভাটাটিতে কাজ করে কত উপার্জন করে?	Skip IN3, If I0=2

QSL	QUES: ENG	QUES: BNG	Skip Note
	1. From wages or salary [___]<br>2. Extra hours [___]<br>3. Value of meals [___]<br>4. Value of housing [___]<br>5. Value of transportation [___]<br>Total amount (Enumerator to fill in) [___]	মজুরি বা বেতন থেকে [___]<br>অতিরিক্ত ঘন্টা [___]<br>খাবারের জন্য [___]<br>আবাসন জন্য [___]<br>পরিবহণের জন্য [___]<br>মোট পরিমাণ (সাক্ষাৎকার গ্রহণকারী পূরণ করবে) [___]	
IN4a	How much does your family earn per week (on average) from working in the kiln?<br><br>1. From wages or salary [___]<br>2. Extra hours [___]<br>3. Value of meals [___]<br>4. Value of housing [___]<br>5. Value of transportation [___]<br>Total amount (Enumerator to fill in) [___]	সপ্তাহে (গড়ে) আপনার পরিবার ভাটাটিতে কাজ করে কত উপার্জন করে?<br><br>মজুরি বা বেতন থেকে [___]<br>অতিরিক্ত ঘন্টা [___]<br>খাবারের জন্য [___]<br>আবাসন জন্য [___]<br>পরিবহণের জন্য [___]<br>মোট পরিমাণ (সাক্ষাৎকার গ্রহণকারী পূরণ করবে) [___]	Skip if M7=1 and I0=2
IN5	In this current firing season, did you get any of the following benefits, bonuses, or incentives from the sardar? Read out all options<br><br>0= Nothing was offered<br>1= Bonus<br>2= Wage increase<br>3= Extra meal<br>4= Motorcycle<br>5= Mobile phone<br>6= Other electronic devices<br>7= Travel allowance for to-and-from travel from home<br>8= Clothing<br>9= Return bonus<br>10= Bakhshish<br>77= Other (specify)	এই ফায়ারিং মৌসুমে, আপনি কি সর্দারের কাছ থেকে নিম্নলিখিত কোনও সুবিধা, বোনাস বা প্রণোদনা পেয়েছেন? সমস্ত বিষয়গুলো উল্লেখ্য করুন<br><br>0= কিছুই দেওয়া হয়নি<br>1= বোনাস<br>2= বেতন বৃদ্ধি<br>3= অতিরিক্ত খাবার<br>4= মটরসাইকেল<br>5= মোবাইল ফোন<br>6= অন্যান্য বিদ্যুৎতিক দব্য<br>7= বাড়ি থেকে আসা-যাওয়ার খরচ<br>8= পোশাক<br>9= রিটার্ন বোনাস<br>10= বখশিশ<br>77= অন্যান্য (উল্লেখ্য করুন)	If I0=1 and IN5=0, then go to IN9, else go to IN6
IN5a	In this current firing season, did you provide get any of the following benefits, bonuses, or incentives from the sardar to your workers? Read out all options<br><br>0= Nothing was offered<br>1= Bonus<br>2= Wage increase<br>3= Extra meal<br>4= Motorcycle<br>5= Mobile phone<br>6= Other electronic devices<br>7= Travel allowance for to-and-from travel from home<br>8= Clothing<br>9= Return bonus<br>10= Bakhshish<br>77= Other (specify)	এই ফায়ারিং মৌসুমে, আপনি কি আপনার কর্মীদের সর্দারের কাছ থেকে নিম্নলিখিত কোনও সুবিধা, বোনাস বা প্রণোদনা দিয়েছেন? সমস্ত বিষয়গুলো উল্লেখ্য করুন<br><br>0= কিছুই দেওয়া হয়নি<br>1= বোনাস<br>2= বেতন বৃদ্ধি<br>3= অতিরিক্ত খাবার<br>4= মটরসাইকেল<br>5= মোবাইল ফোন<br>6= অন্যান্য বিদ্যুৎতিক দব্য<br>7= বাড়ি থেকে আসা-যাওয়ার খরচ<br>8= পোশাক<br>9= রিটার্ন বোনাস<br>10= বখশিশ<br>77= অন্যান্য (উল্লেখ্য করুন)	If I0=2 and IN5a=0, then go to IN9, else go to IN6
IN6	If IN5/IN5a = 1,2,7,9,10 is selected How much was offered (in BDT) [if don't know then put 9999]	যদি IN5/IN5a=1/2/7/9/10 হয়, তবে কতটা দেওয়া হয়েছিল (টাকায়) [যদি কতটাকা বোনাস দিবে সে সম্পর্কে না জানে তবে ৯৯৯৯ বসান]	

QSL	QUES: ENG	QUES: BNG	Skip Note
	1= Bonus [___]<br>2= Wage increase [___]<br>7= Travel allowance for to-and-from travel from home [___]<br>9= Return bonus [___]<br>10= Bakhshish [___]	1= বোনাস [___]<br>2= বেতনবৃদ্ধি [___]<br>7= বাড়ি থেকে আসা-যাওয়ার খরচ [___]<br>9= রিটার্ন বোনাস [___]<br>10= বখশিশ [___]	
IN7	If IN5/IN5a = 3,4, 5, 6 is selected, how many were offered? [1-5]<br><br>3= Extra meal [___]<br>4= Motorcycle [___]<br>5= Mobile phone [___]<br>6= Other electronic devices [___]	যদি IN5/IN5a=3/4/5/6 হয়, তবে কতবার দেওয়া হয়েছিল? [1-5]<br><br>3= অতিরিক্ত খাবার [___]<br>4= মোটরসাইকেল [___]<br>5= মোবাইল ফোন [___]<br>6= অন্যান্য বিদ্যুৎতিক জিনিস [___]	
IN8	Was the wage increase conditional on kiln performance, such as quantity of class-1 bricks or total number of bricks produced?<br><br>1=Yes; 0=No	ভাটার কোনও পারফরম্যান্সের শর্তসাপেক্ষে কি মজুরী বৃদ্ধির করা হয়ছিল, যেমন ক্লাস-১ ইটের উৎপাদন বৃদ্ধি বা মোট ইটের পরিমাণ বৃদ্ধি?<br><br>1=হ্যাঁ; 0=না	If IN6=2, then answer this question
IN8a	What was the specific performance metric for the wage increase?<br><br>Enumerator instruction: an example of a performance metric is "if the kiln produces 80% class-1 bricks"	মজুরি বৃদ্ধির জন্য নির্দিষ্ট পারফরম্যান্সে নির্দেশিকা কি ছিল?<br><br>সাক্ষাৎগ্রহণকারী নির্দেশনা: পারফরম্যান্সে একটি উদাহরণ হল "যদি ভাটা 80% ক্লাস-1 ইট উৎপাদন করে"	If IN8=1, then answer this question
IN8b	Was the wage increase conditional on kiln performance, such as quantity of class-1 bricks or total number of bricks produced?<br><br>1=Yes; 0=No	ভাটার কোনও পারফরম্যান্সের শর্তসাপেক্ষে কি বাড়তি খাবার দেয়া হয়ছিল, যেমন ক্লাস-১ ইটের উৎপাদন বৃদ্ধি বা মোট ইটের পরিমাণ বৃদ্ধি?<br><br>1=হ্যাঁ; 0=না	If IN7=1, then answer this question
IN8c	What was the specific performance metric for the wage increase?<br><br>Enumerator instruction: an example of a performance metric is "if the kiln produces 80% class-1 bricks"	বাড়তি খাবারের জন্য নির্দিষ্ট পারফরম্যান্সে নির্দেশিকা কি ছিল?<br><br>সাক্ষাৎগ্রহণকারী নির্দেশনা: পারফরম্যান্সে একটি উদাহরণ হল "যদি ভাটা 80% ক্লাস-1 ইট উৎপাদন করে"	If IN8b=1, then answer this question
IN8d	Was the bouns conditional on kiln performance, such as quantity of class-1 bricks or total number of bricks produced?<br><br>1=Yes; 0=No	ভাটার কোনও পারফরম্যান্সের শর্তসাপেক্ষে কি বোনাস দেয়া হয়ছিল, যেমন ক্লাস-১ ইটের উৎপাদন বৃদ্ধি বা মোট ইটের পরিমাণ বৃদ্ধি?<br><br>1=হ্যাঁ; 0=না	If IN7=1, then answer this question
IN8e	What was the specific performance metric for the bouns?<br><br>Enumerator instruction: an example of a performance metric is "if the kiln produces 80% class-1 bricks"	বোনাস এর জন্য নির্দিষ্ট পারফরম্যান্সে নির্দেশিকা কি ছিল?<br><br>সাক্ষাৎগ্রহণকারী নির্দেশনা: পারফরম্যান্সে একটি উদাহরণ হল "যদি ভাটা 80% ক্লাস-1 ইট উৎপাদন করে"	If IN8d=1, then answer this question
IN9	In this current firing season, did the owner offer you anything directly? (Multiple answer possible)<br><br>0= Nothing was offered<br>1= Bonus<br>2= Wage increase<br>3= Extra meal<br>4= Motorcycle<br>5= Mobile phone	এই ফায়ারিং মৌসুমে, ভাটার মালিক আপনাকে কি সরাসরি কিছু দিয়েছিলেন? (একাধিক উত্তর প্রযোজ্য)<br><br>0= কিছুই দেওয়া হয়নি<br>1= বোনাস<br>2= বেতনবৃদ্ধি<br>3= অতিরিক্ত খাবার<br>4= মোটরসাইকেল	If IN9=0, then go to IN14, else go to IN10

QSL	QUES: ENG	QUES: BNG	Skip Note
	6= Other electronic devices<br>7= Travel allowance for to-and-from travel from home<br>8= Clothing<br>9= Return bonus<br>10= Bakhshish<br>77= Other (specify)	5= মোবাইল ফোন<br>6= অন্যান্য বিদ্যুৎতিক যন্ত্র<br>7= বাড়ি থেকে আসা-যাওয়ার খরচ<br>8= পোশাক<br>9= রিটার্ন বোনাস<br>10= বখশিশ<br>77= অন্যান্য (উল্লেখ্য করুন)	
IN10	If IN9 = 1,2,7,9,10 is selected How much was offered (in BDT)<br><br>1= Bonus [___]<br>2= Wage increase [___]<br>7= Travel allowance for to-and-from travel from home [___]<br>9= Return bonus [___]<br>10= Bakhshish [___]	যদি IN9=1/2/7/9/10 হয়, তবে কতটা দেওয়া হয়েছিল (টাকায়)<br><br>1= বোনাস [___]<br>2= বেতনবৃদ্ধি [___]<br>7= বাড়ি থেকে আসা-যাওয়ার খরচ [___]<br><br>9= রিটার্ন বোনাস [___]<br>10= বখশিশ [___]	
IN11	If IN9= 3,4, 5, 6 is selected, how many were offered? [1-5]<br><br>3= Extra meal [___]<br>4= Motorcycle [___]<br>5= Mobile phone [___]<br>6= Other electronic devices [___]	যদি IN9=3/4/5/6 হয়, তবে কতবার দেওয়া হয়েছিল? [1-5]<br><br>3= অতিরিক্ত খাবার [___]<br>4= মোটরসাইকেল [___]<br>5= মোবাইল ফোন [___]<br>6= অন্যান্য বিদ্যুৎতিক জিনিস [___]	
IN12	**"[for each option of wage increase, extra meal, or bonus selected]"**<br>Was this conditional on kiln performance, such as quantity of class-1 bricks or total number of bricks produced?<br><br>1=Yes; 0=No	**"[মজুরি বৃদ্ধি, অতিরিক্ত খাবার, বা বোনাস নির্বাচিত প্রতিটি অপশনের জন্য]"**<br>উপরের সকল বোনাস দেয়া হয় ভাটার পারফরম্যান্সে উপর ভিত্তি করে, এই শর্তসাপেক্ষে; যেমন ক্লাস-১ ইট পরিমাণ বা মোট উৎপাদিত ইটের সংখ্যা ছিল?<br><br>1=হ্যাঁ; 0=না	If IN12= 0, then go to IN14, else go to IN13
IN13	If yes, provide detail on what the specific performance metric was<br><br>**Enumerator instruction:** *an example of a performance metric is "if the kiln produces 80% class-1 bricks"*	যদি হ্যাঁ, নির্দিষ্ট কর্মক্ষমতা মানদন্ড কি ছিল তার বিস্তারিত প্রদান করুন<br><br>***সাক্ষাৎকার গ্রহনকারীর নোট:*** *কর্মক্ষমতা মানদন্ড এর একটি উদাহরণ হল "যদি ভাটা 80% ক্লাস-1 ইট উৎপাদন করে"*	
IN14	Did you expect a bonus? (can include any incentive)<br><br>1=Yes; 0=No	আপনি কি বোনাস আশা করেছিলেন? (IN9 এ যেকোন প্রণোদনা হতে পারে)<br><br>1=হ্যাঁ; 0=না	If IN14=0, then go to TR1.
IN15	if IN14=Y, why did you expect it?<br>0=The owner announced it earlier<br>1= The sardar announced it earlier<br>2= I heard it from others in the kiln<br>77= Other (Specify)	যদি IN14=1 হয়, তবে আপনি কেন বোনাস আশা করেছিলেন?<br>0= মালিক এটি আগে ঘোষণা করেছিলেন<br>1= সর্দার এটি আগে ঘোষণা করেছিল<br>2= আমি এটি ভাটায় অন্যদের কাছ থেকে শুনেছি<br>77= অন্যান্য (নির্দিষ্ট করুন)	Skip If I0=2
IN15a	if IN14=Y, why did you expect it?<br><br>0=The owner announced it earlier<br>1= I heard it from others in the kiln<br>77= Other (Specify)	যদি IN14=1 হয়, তবে আপনি কেন বোনাস আশা করেছিলেন?<br><br>0= মালিক এটি আগে ঘোষণা করেছিলেন<br>1= আমি এটি ভাটায় অন্যদের কাছ থেকে শুনেছি<br>77= অন্যান্য (নির্দিষ্ট করুন)	Skip IN15a, if I0=1
IN16	What type of bonus did you expect?	আপনি কি ধরণের বোনাস আশা করেন?	

QSL	QUES: ENG	QUES: BNG	Skip Note
	0= Nothing<br>1= Bonus<br>2= Wage increase<br>3= Extra meal<br>4= Motorcycle<br>5= Mobile phone<br>6= Other electronic devices<br>7= Travel allowance for to-and-from travel from home<br>8= Clothing<br>9= Return bonus<br>10= Bakhshish<br>77= Other (specify)	0= কিছুই না<br>1= বোনাস<br>2= বেতনবৃদ্ধি<br>3= অতিরিক্ত খাবার<br>4= মোটরসাইকেল<br>5= মোবাইল ফোন<br>6= অন্যান্য বিদ্যুৎতিক যন্ত্র<br>7= বাড়ি থেকে আসা-যাওয়ার খরচ<br>8= পোশাক<br>9= রিটার্ন বোনাস<br>10= বখশিশ<br>77= অন্যান্য (উল্লেখ্য করুন)	
IN17	If IN16 = 1,2,7,9,10 is selected How much was offered (in BDT) [if don't know then put 9999]<br><br>1= Bonus [___]<br>2= Wage increase [___]<br>7= Travel allowance for to-and-from travel from home [___]<br>9= Return bonus [___]<br>10= Bakhshish [___]	যদি IN16=1/2/7/9/10 হয়, তবে কতটা দেওয়া হয়েছিল (টাকায়) [যদি কতটাকা বোনাস দিবে সে সম্পর্কে না জানে তবে ৯৯৯৯ বসান]<br><br>1= বোনাস [___]<br>2= বেতনবৃদ্ধি [___]<br>7= বাড়ি থেকে আসা-যাওয়ার খরচ [___]<br><br>9= রিটার্ন বোনাস [___]<br>10= বখশিশ [___]	
IN18	If IN16= 3,4, 5, 6 is selected, how many were offered? [1-5]<br><br>3= Extra meal [___]<br>4= Motorcycle [___]<br>5= Mobile phone [___]<br>6= Other electronic devices [___]	যদি IN16=3/4/5/6 হয়, তবে কতবার দেওয়া হয়েছিল? [1-5]<br><br>3= অতিরিক্ত খাবার [___]<br>4= মোটরসাইকেল [___]<br>5= মোবাইল ফোন [___]<br>6= অন্যান্য বিদ্যুৎতিক জিনিস [___]	
IN19	Is what you received different than what you expected?<br><br>1=Yes; 0=No	আপনি যা পেয়েছিলেন তা কি প্রত্যাশার চেয়ে কম ছিল?<br><br>1=হ্যাঁ; 0=না	
IN20	if IN19=Yes, why was it different?<br>0= Owner changed his mind<br>1= We heard about it but never got it from the sardar<br>2= Kiln performance was not as expected<br>77= Other (Specify)	যদি IN19=হ্যাঁ হয়, তবে কেন পার্থক্য ছিল?<br>0 = মালিক তার মন পরিবর্তন করেছেন<br>1 = আমরা এটি সম্পর্কে শুনেছি কিন্তু সর্দারের কাছ থেকে এটা পাইনি<br>2 = ভাটার উৎপাদন প্রত্যাশার মতো ছিল না<br>77 = অন্যান্য (নির্দিষ্ট করুন)	Skip If I0=2
IN20a	if IN19=Yes, why was it different?<br>0= Owner changed his mind<br>1= Kiln performance was not as expected<br>77= Other (Specify)	যদি IN19=হ্যাঁ হয়, তবে কেন পার্থক্য ছিল?<br>0 = মালিক তার মন পরিবর্তন করেছেন<br>1 = ভাটার উৎপাদন প্রত্যাশার মতো ছিল না<br>77 = অন্যান্য (নির্দিষ্ট করুন)	Skip If I0=1

Module: Core indicators

QSL	QUES: ENG	QUES: BNG	Skip Note
	**Read aloud:** *We are now going to talk about different work situations you have experienced. In responding, please think*	**পড়ুন:** *আমরা এখন আপনার অভিজ্ঞতার বিভিন্ন কাজের পরিস্থিতি সম্পর্কে কথা বলতে যাচ্ছি। প্রতিক্রিয়া জানাতে, অনুগ্রহ করে ইট ভাটায় পূর্ববর্তী সমস্ত কাজ সম্পর্কে চিন্তা*	Skip TR5 to TR8, if I0=2

QSL	QUES: ENG	QUES: BNG	Skip Note
	about all previous jobs at brick kilns, including this kiln and any other kiln you may have worked at. Please also remember your answers are confidential and will never be communicated to any employers or sardars.	*করুন, এই ভাটা সহ এবং অন্য যে কোন ভাটিতে আপনি কাজ করেছেন। অনুগ্রহ করে মনে রাখবেন আপনার উত্তরগুলি গোপনীয় এবং কখনই কোন নিয়োগকর্তা বা সর্দারদের সাথে যোগাযোগ করা হবে না।*	
	Skip TR4 to T7 if I0=2		
TR4	Have you ever worked at a brick kiln without a formal contract?<br><br>1= I have rarely or never had a formal contract<br>2 = I sometimes work without a formal contrat<br>3 = I always have a formal contract	আপনার কি কখনও কোন নির্দিষ্ট চুক্তি ছাড়া কোন ভাটায় কাজ করেছেন?<br><br>1 = আমি খুব কমই বা কোন আনুষ্ঠানিক চুক্তি করিনি<br>2 = আমি মাঝে মাঝে আনুষ্ঠানিক চুক্তি ছাড়াই কাজ করি<br>3 = আমি সবসময় একটি আনুষ্ঠানিক চুক্তি আছে	
TR5	How did you usually find jobs at kilns?<br><br>1= Hired directly by the sardar (not previously known)<br>2= Friend or family member (can be the sardar)<br>3= Third party recruiter	আপনি সাধারণত কিভাবে ভাটার কাজটি খুঁজে পান?<br><br>1= সরাসরি সর্দার কর্তৃক নিয়োগকৃত (আগের পরিচিত নয়)<br>2= বন্ধু বা পরিবারের সদস্য (সর্দার হতে পারে)<br>3= তৃতীয় পক্ষের নিয়োগকারী	If TR5=3, then go to TR6, else go to TR7
TR6	If TR5=3 Have you ever found that a job at a kiln was different from what the sardar said? (multiple answer possible)<br><br>1 = No, my jobs have always been the same or better than promised<br>2 = More difficult work than said<br>3 = More hours than said<br>4 = Worse pay than said or incomplete pay<br>5 = Working conditions were worse<br>6 = Living conditions or housing were worse<br>7 = Legal documentation was not given<br>8 = Job was not in the promised location<br>9 = Other differences	যদি TR5=3 হয়, তবে সর্দার যা বলেছিল তার সাথে কাজটির কি মিল রয়েছে? (একাধিক উত্তর প্রযোজ্য)<br><br>1 = না, আমার কাজ সবসময় একই বা প্রতিশ্রুতি চেয়ে ভাল<br>2 = যা বলেছে তার চেয়ে আরও কঠিন কাজ<br>3 = যা বলেছে তার চেয়ে বেশি ঘন্টার কাজ<br>4 = যা বলেছে তার চেয়ে কম বা অসম্পূর্ণ বেতন<br>5 = যা বলেছে কাজের পরিবেশে আরও খারাপ<br>6 = বসবাসের অবস্থা বা আবাসন ব্যবস্থা আরও খারাপ ছিল<br>7 =আইন কোন কাগজপত্র দেয়া হয়নি<br>8 = না, যে জায়গায় কাজের কথা বলেছে প্রতিশ্রুত অনুযায়ী সে জায়গায় কাজ ছিল না<br>9 = অন্যান্য পার্থক্য	
TR7	Sometimes, people are coerced, tricked, or forced to work at a job against their will. This can happen through direct or implied threats or consequences. Have you ever felt coerced or forced when you were recruited to work for a job at a kiln?<br>1 = Yes, by force<br>2 = Yes, by threat<br>3 = No, not coerced	কখনও কখনও, শ্রমিকদের জোর করে, প্রতারিত করে বা তাদের ইচ্ছার বিরুদ্ধে কাজ করতে বাধ্য করা হয়। এটি সরাসরি বা অন্তর্নিহিত হুমকি বা পরিণতির মাধ্যমে ঘটতে পারে। আপনি কি কখনো জবরদস্তি বা বাধ্যতা অনুভব করেছেন যখন কোন ইট ভাটায় চাকরির জন্য আপনাকে নিয়োগ করা হয়েছিল?<br>1 = হ্যাঁ, জোর করে<br>2 = হ্যাঁ, হুমকি দ্বারা<br>3 = না, জোর করা হয়নি	
TR8	Have you ever been confined or held against your will by a recruiter/sardar?<br><br>1 = Yes, sometimes<br>2 = Yes, always<br>3 = No, never	আপনি কি কখনও একজন নিয়োগকারী/সর্দার দ্বারা আপনার ইচ্ছার বিরুদ্ধে বন্দী বা আটকে পড়েছেন?<br><br>1 = হ্যাঁ, কখনও কখনও<br>2 = হ্যাঁ, সর্বদা<br>3 = না, কখনই না	

QSL	QUES: ENG	QUES: BNG	Skip Note
TR8a	Have you ever been confined or held against your will by an employer/owner?<br><br>1 = Yes, sometimes<br>2 = Yes, always<br>3 = No, never	আপনি কি কখনও একজন নিয়োগকর্তা/মালিক দ্বারা আপনার ইচ্ছার বিরুদ্ধে বন্দী বা আটকে পড়েছেন?<br><br>1 = হ্যাঁ, কখনও কখনও<br>2 = হ্যাঁ, সর্বদা<br>3 = না, কখনই না	Skip TR8a, if I0=1
	Skip TR10 to TR10b, if I0=2		
TR10	Did you ever have to repay any expenses for recruiting after accepting a job at a kiln, including cost of transportation, food, or other expenses?<br><br>1 = No, there have never been costs<br>2 = No, I always pay my own expenses<br>3 = Yes, I have had to repay, or still owe a debt to one or more recruiters	একটি ভাটায় চাকরি গ্রহণ করার পর আপনাকে কি কখনও নিয়োগের জন্য কোন খরচ পরিশোধ করতে হয়েছে, যার মধ্যে পরিবহন খরচ, খাবার বা অন্যান্য খরচ রয়েছে?<br><br>1 = না, কোনও খরচ করতে হয়নি<br>2 = না, আমি আমার নিজের খরচ দিয়েছি<br>3 = হ্যাঁ আমাকে টাকা পরিশোধ করতে হয়েছিল, বা এখনও আমার নিয়োগকারী টাকা পায়	
TR10a	If yes, about how much are the expenses for recruiting usually? (98=Not remember)	যদি হ্যাঁ, সাধারণত নিয়োগের জন্য কত খরচ হয়? (মনে নাই=98)	
TR10b	If yes, was your debt deducted from your pay?<br><br>1=Yes; 0=No	যদি হ্যাঁ, আপনার টাকা কি আপনার বেতন থেকে কেটে নেওয়া হয়?<br>1=হ্যাঁ; 0=না	
TR11	Sometimes, people work for employers who do not let them leave their jobs. Have you ever felt you were not able to stop working at a brick kiln job if you wanted?<br><br>1=Yes; 0=No; 2= No because of advance salary payment	কখনও কখনও, লোকেরা নিয়োগকারীদের জন্য কাজ করে যারা তাদের চাকরি ছাড়তে দেয় না। আপনি কি কখনও অনুভব করেছেন যে আপনি চাইলে ইট ভাটায় কাজ করা বন্ধ করতে পারবেন না?<br><br>1=হ্যাঁ; 0=না; 2= না, কারণ মজুরী বাবদ অগ্রীম টাকা নিয়েছে	
TR11a	If TR11=1, Have you ever felt that if you left a job at a kiln, that you would not receive all the wages you are owed?<br><br>1=Yes; 0=No	যদি TR11=1, আপনি যদি চাকরি ছেড়ে দেন, আপনি কি মনে করেন যে আপনি আপনার পাওনা সমস্ত মজুরি পাবেন?<br><br>1=হ্যাঁ; 0=না	Skip TR11a, if I0=2
TR12	Do you ever have deductions made from your wages for food, housing, interest, or debt (your own food/housing/debt or that of your workers)?<br><br>1 = No deductions have been made<br>2 = Yes, but the deductions seemed fair<br>3 = Yes, and the deductions seemed unfair (for example, because the prices charged for food were too high, the interest rate was too high, or because I was being cheated)	কখনও কি খাদ্য, আবাসন, সুদ বা ঋণ এর জন্য আপনার মজুরি থেকে টাকা কাটা হয়েছে?<br><br>1= না কখনো কোন টাকা কাটা হয়নি<br>2= হ্যাঁ, তবে টাকা কাটা ন্যায্য ছিল<br>3= হ্যাঁ, কাটা হয়েছে এবং বিষয়টি যৌক্তিক ছিলনা (উদাহরণঃ খাবারের জন্য চার্জ করা দামগুলি খুব বেশি, সুদের হার খুব বেশি, বা আমাকে প্রতারণা করা হয়েছে)	
	**Read aloud: Now I want to talk about the number of hours you tned to work.**	**পড়ুন: এখন আমি আপনি কত ঘন্টা কাজ করেন সে সম্পর্কে কথা বলতে চাই।**	
TR13	How many hours per day do you usually work when you work at brick kilns?	আপনি যখন ইটের ভাটায় কাজ করেন তখন আপনি সাধারণত প্রতিদিন কত ঘন্টা কাজ করেন?	
TR14	How many days per week do you usually work when you work at brick kilns?	আপনি যখন ইট ভাটায় কাজ করেন তখন আপনি সাধারণত প্রতি সপ্তাহে কত দিন কাজ করেন?	

QSL	QUES: ENG	QUES: BNG	Skip Note
TR15	Have you ever been able to take any leave or time off from a brick kiln job?<br><br>1= I can never take leave<br>2= I have sometimes been able to take leave<br>3= I can always take leave<br>4= Can’t take leave	আপনি কি কখনও একটি ইট ভাটার কাজ থেকে কোন ছুটি বা সময় নিতে সক্ষম হয়েছেন?<br><br>1= আমি কখনই ছুটি নিতে পারি না<br>2= মাঝে মাঝে ছুটি নিতে পেরেছি<br>3= আমি সবসময় ছুটি নিতে পারি<br>4= ছুটি নিতে পারিনা	
	Skip TR16 to TR19, if I0=2		
TR16	Have you ever been made to work longer hours than expected?<br><br>1=Yes, always<br>2 = Yes, sometimes<br>3= No, never	আপনি কি কখনও প্রত্যাশিত সময়ের চেয়ে বেশি ঘন্টা কাজ করতে বাধ্য হয়েছেন?<br><br>1=হ্যাঁ, সবসময়<br>2 = হ্যাঁ, মাঝে মাঝে<br>3= না, কখনই না	
TR17	Have you ever been made to be available day and night at a kiln job?<br><br>1 = Yes, always<br>2 = Yes, sometimes<br>3 = No never	আপনি কি কখনও একটি ভাটা দিনরাত কাজ করা হয়েছে?<br><br>1=হ্যাঁ, সবসময়<br>2 = হ্যাঁ, মাঝে মাঝে<br>3= না, কখনই না	
TR18	Have you ever been compensated for this overtime?<br><br>1=Yes, always paid extra for overtime<br>2=Yes, sometimes paid extra for overtime<br>3=No, never paid extra for overtime	আপনি কি ওভারটাইমের জন্য আপনার প্রাপ্য মজুরী পেয়েছিলেন?<br><br>1=হ্যাঁ, ওভারটাইমের জন্য সবসময় অতিরিক্ত অর্থ প্রদান করা হয়<br>2=হ্যাঁ, কখনও কখনও ওভারটাইমের জন্য অতিরিক্ত অর্থ প্রদান করা হয়<br>3=না, ওভারটাইমের জন্য কখনই অতিরিক্ত অর্থ প্রদান করবেন না	
TR19	Have you ever felt like you would face retaliation if you refuse to work overtime? This could be punishments, financial penalties, or being fired<br><br>1 = I have felt like I would face punishments or penalties<br>2 = I have felt like I would be fired<br>3 = I have never felt like there would be consequences for not working overtime<br>88= Not applicable	আপনি কি কখনো অনুভব করেছেন যে আপনি ওভারটাইম কাজ করতে অস্বীকার করলে আপনি প্রতিশোধের সম্মুখীন হবেন? এটি হতে পারে শাস্তি, আর্থিক জরিমানা বা চাকরিচ্যুত করা<br><br>1 = আমি অনুভব করেছি যে আমি শাস্তি বা শাস্তির সম্মুখীন হব<br>2 = আমি অনুভব করেছি যে আমাকে বরখাস্ত করা হবে<br>3 = আমি কখনই অনুভব করিনি যে ওভারটাইম কাজ না করার পরিণতি হবে<br>88 = প্রযোজ্য নয়	

Control and Surveillance

QSL	QUES: ENG	QUES: BNG	Skip Note
TR20	Sometimes employers exert control over the people who work for them, even during their free time. Have any brick kiln employers or sardars ever attempted to control your personal life in any of these ways?<br>(select any you have experienced answer possible)<br><br>1= Through blackmail<br>2= By threatening religious retribution<br>3= By threatening to exclude you or your family from future employment<br>4= By threatening to or actually isolating you from your family<br>5= By threatening to or actually isolating you from your friends (being ostracised)<br>6= By threatening family members<br>0= Never experienced any of these<br>9=Did not respond	কখনও কখনও নিয়োগকর্তারা তাদের জন্য যারা কাজ করেন তাদের উপর নিয়ন্ত্রণ প্রয়োগ করেন, এমনকি তাদের অবসর সময়েও। কোন ইট ভাটা মালিক বা সর্দার কি কখনও এই উপায়ে আপনার ব্যক্তিগত জীবন নিয়ন্ত্রণ করার চেষ্টা করেছেন? (আপনার সম্ভাব্য উত্তর আছে এমন যেকোনো একটি নির্বাচন করুন)<br><br>1= ব্ল্যাকমেইল মাধ্যমে<br>2= ধর্মীয় প্রতিশোধের হুমকি দিয়ে<br>3= আপনাকে বা আপনার পরিবারকে ভবিষ্যতের কর্মসংস্থান থেকে বাদ দেওয়ার হুমকি দিয়ে<br>4= আপনার পরিবার থেকে আপনাকে বিচ্ছিন্ন /আলাদা করে দেবার হুমকি দেয়<br>5= আপনাকে আপনার বন্ধুদের থেকে বিচ্ছিন্ন (বের করে দেয়ার) করার হুমকি দেয়<br>6= পরিবারের সদস্যদের হুমকি দিয়ে<br>0= এগুলোর কোনো অভিজ্ঞতা হয়নি কখনো<br>9= কোন উত্তর দেয় নাই	Skip if I0=2
TR20a	Sometimes employers exert control over the people who work for them, even during their free time. Have any brick kiln owners ever attempted to control your personal life in any of these ways? (select any you have experienced answer possible)<br><br>1= Through blackmail<br>2= By threatening religious retribution<br>3= By threatening to exclude you or your family from future employment<br>4= By threatening to or actually isolating you from your family<br>5= By threatening to or actually isolating you from your friends (being ostracised)<br>6= By threatening family members<br>0= Never experienced any of these<br>9=Did not respond	TR20a. কখনও কখনও নিয়োগকর্তারা তাদের জন্য যারা কাজ করেন তাদের উপর নিয়ন্ত্রণ প্রয়োগ করেন, এমনকি তাদের অবসর সময়েও। কোন ইট ভাটা মালিক কি কখনও এই উপায়ে আপনার ব্যক্তিগত জীবন নিয়ন্ত্রণ করার চেষ্টা করেছেন? (আপনার সম্ভাব্য উত্তর আছে এমন যেকোনো একটি নির্বাচন করুন)<br><br>1= ব্ল্যাকমেইল মাধ্যমে<br>2= ধর্মীয় প্রতিশোধের হুমকি দিয়ে<br>3= আপনাকে বা আপনার পরিবারকে ভবিষ্যতের কর্মসংস্থান থেকে বাদ দেওয়ার হুমকি দিয়ে<br>4= আপনার পরিবার থেকে আপনাকে বিচ্ছিন্ন /আলাদা করে দেবার হুমকি দেয়<br>5= আপনাকে আপনার বন্ধুদের থেকে বিচ্ছিন্ন (বের করে দেয়ার) করার হুমকি দেয়<br>6= পরিবারের সদস্যদের হুমকি দিয়ে<br>0= এগুলোর কোনো অভিজ্ঞতা হয়নি কখনো<br>9=কোন উত্তর দেয় নাই	Skip if I0=1
TR21	Sometimes employers may interfere with employee's personal property. Has any employer or sardar ever confiscated your mobile phone, or limited methods of communication?<br><br>Mobile phone: (1= Yes, often; 2= Yes, sometimes; 0 = No; 9=Did not respond)<br><br>Identification or travel documents: (1= Yes, often; 2= Yes, sometimes; 0= No; 9=Did not respond)	কখনও কখনও মালিকরা শ্রমিকদের ব্যক্তিগত সম্পত্তিতে হস্তক্ষেপ করতে পারে। আপনার মালিক বা অন্য কোনও ব্যক্তি কি আপনার মোবাইল ফোন বা যোগাযোগের অন্য মাধ্যম নিয়েগিয়েছিল?<br><br>মোবাইল ফোনঃ (1= হ্যাঁ, প্রায়ই; 2= হ্যাঁ, কখনও কখনও; 0= না; 9=কোন উত্তর দেয় নাই)<br>পরিচয় বা ভ্রমণের নথিঃ (1= হ্যাঁ, প্রায়শই; 2= হ্যাঁ, কখনও কখনও; 0= না; 9=কোন উত্তর দেয় নাই)	
TR22	Sometimes employees limit or monitor their employees even when they are not working (or in off hours). Has any employer or sardar ever:	কখনও কখনও মালিকরা যখন তাদের কর্মীদের কাজ না তখনও তাদের উপর নজরদারি করে (বা বিরতির সময়ও) । আপনার নিয়োগকর্তা বা তার কোন লোক কি কখনও এই কাজ করেছেন:	

QSL	QUES: ENG	QUES: BNG	Skip Note
	**[1= Yes, often; 2= Yes, sometimes; 0 = No; 9=Did not respond]**<br>Monitored your movement when not working<br>Limited your movement when not working<br>Monitored your communication when not working<br>Limited your communication when not working<br>Supervised your communication when not working<br>Supervised or watch you during non-work hours	**[1= হ্যাঁ, প্রায়ই; 2= হ্যাঁ, কখনও কখনও; 0= না;** 9=কোন উত্তর দেয় নাই]<br>কাজ না করার সময় আপনার চলা-ফেরা পর্যবেক্ষণ করুন<br>কাজ না করার সময় আপনার চলা-ফেরা নিয়ন্ত্রণ করা<br>কাজ না করার সময় আপনার যোগাযোগ পর্যবেক্ষণ করা<br>কাজ না করার সময় আপনার যোগাযোগ নিয়ন্ত্রণ করা<br>কাজ না করার সময় আপনার যোগাযোগের নজরদারি করা<br>আপনি যখন কাজ করেন না তখনও আপনাকে তদারকি বা নজরদারিতে রাখা	
Debt and Enslavement			
TR23	Were you ever in debt to this or any previous your brick kiln owners?<br>1 = Yes, but it has always been my decision<br>2 = I have had debt I did not agree with imposed on me<br>3 = I have had debt I did not consent to imposed on me<br>4 = No I have never been indebted to my employer<br>9=Did not respond	আপনি কি কখনও আপনার নিয়োগকর্তার কাছ থেকে ঋণ নিয়ে ছিলেন?<br>1 = হ্যাঁ, তবে আমার সিদ্ধান্ত<br>2 = হ্যাঁ, তবে আমি এটির সাথে একমত ছিলাম না<br>3= হ্যাঁ, তবে আমি এটির সাথে একমত হইনি এবং আমি আমার সম্মতি দিইনি<br>4= না, আমি কখনও ঋণ নেইনি<br>9=কোন উত্তর দেয় নাই	

Violence

QSL	QUES: ENG	QUES: BNG	Skip Note
TR24	Sometimes employers use physical violence against their employees. Have you ever experienced any physical violence by any employer, recruiter, or other individuals above you? (multiple answer possible)<br>1 = Yes, against myself<br>2 = Yes, against family members<br>3 = Yes, but directed at others<br>4 = No, but I heard threats of violence<br>5 = No, not even threats<br>9=Did not respond	কখনও কখনও মালিক তাদের কর্মীদের শারীরিক নির্যাতন করে। আপনি কি কখনও আপনার মালিক, নিয়োগকারী বা অন্যান্য ব্যক্তিদের দ্বারা কখনও কোনও শারীরিক নির্যাতনের শিকার হয়েছেন? [একাধিক উত্তর প্রযোজ্য]<br>1 = হ্যাঁ, নিজের সাথে হয়েছে<br>2 = হ্যাঁ, পরিবারের সদস্যদের সাথে হয়েছে<br>3 = হ্যাঁ, তবে অন্যদের সাথে হয়েছে<br>4= না, তবে আমি নির্যাতন করবে এমন হুমকি শুনেছি<br>5= না, এমন কি হুমকিও নয়<br>9=কোন উত্তর দেয় নাই	If TR24=2, go to TR25, else go to OH1
TR25	If TR29=2, Which family member(s) were these threats directed toward?<br>1=Child<br>2=spouse/partner<br>3=other family member?<br>9=Did not respond	পরিবারের কোন সদস্য?<br>1=শিশু<br>2=স্ত্রী<br>3=পরিবারের অন্যান্য সদস্য<br>9=কোন উত্তর দেয় নাই	

Health Effects

QSL	QUES: ENG	QUES: BNG	Skip Note
OH1	Are you exposed to any of the following during your work in kilns?	আপনি কি ভাটায় আপনার কাজের জন্য নিম্নলিখিতগুলির কোনওটির সংস্পর্শে এসেছেন?	
	**[1= Yes; 0 = No]**	**[1= হ্যাঁ, 0= না]**	
	1=Dust, fumes,<br><br>2= Fire, gas, flames, extreme heat<br>3= Loud noise or vibration<br>4 = Dangerous tools (brick cuts, knives, blades etc.)<br>5= Work at heights<br>6= Chemicals (pesticides, glues, explosives etc.)	1= ধূলিকণা, ধোঁয়া, জোরে শব্দ বা কম্পন<br><br>2= আগুন, গ্যাস, অগ্নিশিখা<br>3= প্রচন্ড তাপ<br>4= বিপজ্জনক সরঞ্জাম (ইটের আঘাতে, ছুরি, ব্লেড ইত্যাদি)<br><br>5= উচুতে কাজ করা<br>6= রাসায়নিক (কীটনাশক, আঠালো, বিস্ফোরক ইত্যাদি)	
	**If I4 =1/M, then don't aks the option-6**	**উত্তরদাতা যদি পুরুষ হয়, তবে** OH2-এর কোড 6 **বলবেন না**	
OH2	Because of this job, in the past year did you have	এই কাজের কারণে, গত এক বছরে আপনার ছিল	
	**[1= Yes; 0 = No]**	**[1= হ্যাঁ, 0= না]**	
	1=Any serious injuries or amputation?<br>2=Fracture, twists, displacements, hernia, or pulled muscles?<br>3=Burns by ice, salt, lubricant, fire, hot water, or other chemicals?<br>4=Any respiratory problems such as asthma, bronchitis, tuberculosis, or shortness of breath?<br>5=Blindness or any vision loss?<br>6=Deafness or other hearing problems?<br>**7=Change in your menstrual cycle (for women only)**<br>8=Psychological problems such as depression or panic?<br>9=None	1=কোন গুরুতর আঘাত বা বিচ্ছেদ?<br>2=ফ্র্যাকচার, মোচড়, স্থানচ্যুতি, হার্নিয়া বা টানা পেশী?<br><br>3=বরফ, লবণ, লুব্রিক্যান্ট, আগুন, গরম জল বা অন্যান্য রাসায়নিক দ্বারা পোড়া?<br>4=হাঁপানি, ব্রঙ্কাইটিস, যক্ষ্মা বা শ্বাসকষ্টের মতো কোনও শ্বাসকষ্টজনিত সমস্যা?<br>5=অন্ধত্ব বা কোন দৃষ্টি ক্ষতি?<br>6=বধিরতা নাকি অন্যান্য শ্রবণ সমস্যা?<br>**7=আপনার মাসিকের** সময়কাল **পরিবর্তন (কেবল মহিলাদের জন্য)**<br>8=মানসিক সমস্যা যেমন হতাশা বা আতঙ্ক?<br>9=কিছুই না	
OH4	How many days were you away or unable to work because of this injury?<br><br>00= if not away from work<br>99= Don't know<br>98= if they expect never to return due to injury	এই আঘাতের কারণে আপনি কত দিন দূরে ছিলেন বা কাজ করতে অক্ষম ছিলেন?<br><br>00= যদি কাজ থেকে দূরে না থাকে<br>99= জানেন না<br>98= আঘাতের কারণে কখনও কাজে ফিরে আসতে পারবে না বলে আশা করেছিল	
OH5	Have you ever had any of the following symptoms?<br>**Please Code:**<br>0=Not at all<br>1=Today; 2=Yesterday; 3=In the past 1 week;<br>4=In the past 2 weeks<br>5= Longer than past 2 weeks	আপনার কি নিম্নলিখিত লক্ষণগুলির কোনটি আছে? যদি থকে তবে প্রত্যেকটির জন্য কোড করুনঃ<br>0=একদম না; 1= আজ;<br>2= গতকাল;<br>3= গত 1 সপ্তাহে;<br>4= গত 2 সপ্তাহে<br>5= গত 2 সপ্তাহের চেয়ে বেশি	
	Wheezing/panting/asthma	হুইজিং/প্যান্টিং/হাঁপানী	
	Constant cough	নিয়মিত কাশি	
	Difficulty breathing	শ্বাস নিতে অসুবিধা	
	Fever	জ্বর	
	Skin rash	চামড়া ফুসকুড়ি	
	Diarrhea	ডায়রিয়া	
	Toothache	দাঁতে ব্যথা	

QSL	QUES: ENG	QUES: BNG	Skip Note
	Eye infection/irritation	চোখের সংক্রমণ/জ্বালা	
	Ear infection/irritation	কানের সংক্রমণ/জ্বালা	

Anxiety GAD-7

Over the last 2 weeks, how often have you been bothered by any of the following problems? গত দুই সপ্তাহ বা ১৪ দিনে কতদিন নিচে উল্লখেতি সমস্যা গুলো আপনাকে উদ্বিগ্ন বা বিব্রত করেছিলেন		Not at All (একদম নয়)	Several days (বেশ কয়েকদিন)	More than half of the days (সপ্তাহের অর্ধেক বেশিদিন)	Nearly everyday প্রায় প্রতিদিন
AN1	Feeling nervous, anxious, or on edge<br>কোন কারণে বিচলিত কিংবা উদ্বিগ্ন অনুভব করা	0	1	2	3
AN2	Not being able to sleep, stop or control worrying<br>ঘুমাতে না পারা বা দুশ্চিন্তা দূর করতে না পারা	0	1	2	3
AN3	Worrying too much about different things<br>বিভিন্ন বিষয়ে মাত্রাতিরিক্ত চিন্তা করা	0	1	2	3
AN4	Trouble relaxing<br>বিশ্রাম নিতে অসুবিধা হওয়া (অস্থিরতা)	0	1	2	3
AN5	Being so restless that it is hard to sit still<br>এতো অস্থির লাগছে যে আপনি কোথাও শান্তভাবে বসে থাকতে পারছিলে না	0	1	2	3
AN6	Becoming easily annoyed or irritable<br>সহজেই বিরক্ত রাগান্বিত হয়ে যাওয়া (খিটখিটে মেজাজ)	0	1	2	3
AN7	Feeling afraid, as if something awful might happen<br>সবসময় ভয়ে থাকা যেন খারাপ কিছু ঘটতে যাচ্ছে	0	1	2	3

QSL	QUES: ENG	QUES: BNG	Skip Note
AN8	If you checked any problems, how difficult have they made it for you to do your work, take care of things at home, or get along with other people?<br><br>0. Not difficult at all<br>1. Somewhat difficult<br>2. Very difficult<br>3. Extremely difficult	এগুলো কি আপনার জন্য আপনার কাজ করা, ঘরের কাজ করা বা অন্য মানুষের সাথে থাকা কঠিন করে তুলেছে?<br><br>0. মোটেও কঠিন নয়<br>1. কিছুটা কঠিন<br>2. খুব কঠিন<br>3. অত্যন্ত কঠিন	

PHQ-9

	Over the last 2 weeks, how often have you been bothered by any of the following problems? গত দুই সপ্তাহ বা ১৪ দিনে কতদিন নিচে উল্লখেতি সমস্যা গুলো আপনাকে উদ্বগ্নি বা বিব্রত করেছিলেন	**Not at All** (একদম নয়)	**Several days** (বেশ কয়েকদিন)	**More than half of the days** (সপ্তাহের অর্ধেক বেশিদিন)	**Nearly everyday** প্রায় প্রতিদিন
PHQ1	Little interest of pleasure in doing things (গত দুই সপ্তাহ বা ১৪ দিনে কতদনি আপনি কোন কিছু করতে কম আগ্রহ ও আনন্দ পেয়েছিলেন?)	0	1	2	3
PHQ2	Feeling down, depressed or hopeless (গত দুই সপ্তাহ বা ১৪ দিনে কতদনি আপনার মন মরা ভাব, বিষাদ বা হতাশা বোধ হয়েছিল?)	0	1	2	3
PHQ3	Trouble falling or staying asleep or sleeping too much (গত দুই সপ্তাহ বা ১৪ দিনে কতদনি আপনার ঘুম আসতে বা ঘুমিয়ে থাকতে অসুবিধা হয়েছিল অথবা অতিরিক্ত ঘুম হয়েছিল?)	0	1	2	3
PHQ4	Feeling tired or having little energy (গত দুই সপ্তাহ বা ১৪ দিনে কতদনি আপনার ক্লান্ত অথবা শক্তিহীন/শরীরে বল নাই এমন অনুভব হয়েছিলো?)	0	1	2	3
PHQ5	Poor appetite or over eating (গত দুই সপ্তাহ বা ১৪ দিনে কতদনি আপনার ক্ষিধা কম লেগেছিল বা অতিরিক্ত খেয়েছিলেন?)	0	1	2	3
PHQ6	Feeling bad about yourself—or that you are a failure or have let yourself or your family down (গত দুই সপ্তাহ বা ১৪ দিনে কতদনি আপনার নিজের সম্পর্কে খারাপ ধারণা হয়েছিল অথবা নিজেকে অসফল/ নিজে কিছুই করতে পারেন নাই এমন মনে হয়েছিল, অথবা আপনি নিজেকে বা পরিবারকে হতাশ করেছেন এমন মনে হয়ছেলি ?)	0	1	2	3
PHQ7	Trouble concentrating on things, such as reading the newspaper or watching television (গত দুই সপ্তাহ বা ১৪ দিনে কতদনি আপনার কোন কিছুতে মনোযোগ দিতে সমস্যা হয়েছিল যেমন-খবরের কাগজ পড়া, রান্নাকরা, ঘরের অন্যকোন কাজ অথবা টেলিভিশন দেখা ?)	0	1	2	3
PHQ8	Moving or speaking so slowly that other people could have noticed or the opposite –being so fidgety or restless that you have been around a lot more than usual (গত দুই সপ্তাহ বা ১৪ দিনে কতদনি আপনি এতই আস্তে চলাফেরা করেছেন অথবা কথা বলেছেন যে, হয়ত অন্য মানুষেরা সেটা খেয়াল করেছে । অন্যদিকে আপনি -এত বেশী ছটফট বা অস্থির ছিলেন যে আপনি সাধারনভাবে যেভাবে চলাফেরা করেন তার থেকে বেশি গতিতে চলাফেরা করেছেন ?)	0	1	2	3
PHQ9	Thoughts that you would be better off dead or of hurting yourself in some way (গত দুই সপ্তাহ বা ১৪ দিনে কতদনি আপনি জীবন রাখার চেয়ে না রাখাই ভালো এমন ধারণা পোষণ করেছিলেন অথবা নিজেকে কোন ভাবে আঘাত/ক্ষতি করার কথা ভেবেছিলেন ?)	0	1	2	3

Socio economic status

S1	How many of the following does your family own?<br>1=yes; 0=No	আপনার খানায় নিম্নলিখিত জিনিসগুলো আছে কী? (প্রত্যেকটি জিনিসের আলাদা আলাদা উত্তর দিন।)<br>[1=হ্যাঁ; 0=না]	Number of assets (সংখ্যা লিখুন)
	Radio (working)	রেডিও (এখন কাজ করছে)	>0
	Television (working)	টেলিভিশন (এখন কাজ করছে)	
	Mobile phone	মোবাইল ফোন	
	Non-mobile telephone	অ-মোবাইল ফোন	
	Computer	কম্পিউটার	
	Refrigerator	রেফ্রিজারেটর	
	Almirah or waredrobe	আলমিরাহ বা ওয়ারড্রোব	
	Electric fan	বৈদ্যুতিক পাখা	
	DVD player	ডিভিডি প্লেয়ার	
	Water pump	জল পাম্প	
	IPS/generator	আইপিএস/জেনারেটর	
	Air conditioner	এয়ার কন্ডিশনার	
	Car/truck/minibus	গাড়ি/ট্রাক/মিনিবাস	
	Autobike/tempo/CNG	অটোবাইক/টেম্পো/সিএনজি	
	Rickshaw/van	রিকশা/ভ্যান	
	Bicycle	সাইকেল	
	Motorcycle	মোটরসাইকেল	
	Boat w/motor	নৌকা ডাব্লু/মোটর	
	Boat without motor	মোটর ছাড়া নৌকা	
	Sewing machine	সেলাই যন্ত্র	
	Mobile phone	মোবাইল ফোন	
	A land line	একটি ল্যান্ড লাইন	
S2	How much your household's monthly income (approximate)?	আপনার পরিবারের মাসিক আয় (আনুমানিক) কত?	
	Thank you for you time. If for any reason you wish to talk to someone to help you with problems you are facing<br>999 for immediate help<br>109 for psychological help		
O1	Observation checklist (do not ask the respondent)<br>While on this kiln have you seen any of the following? (Select if yes)	পর্যবেক্ষণ চেকলিস্ট (উত্তরদাতাকে জিজ্ঞাসা করবেন না)<br>এই ইট ভাটাতে থাকাকালীন আপনি নিম্নলিখিত কোনটি দেখেছেন? (যদি হ্যাঁ নির্বাচন করুন)	
	Children who look younger than 18 years molding bricks	18 বছরের কম দেখতে বয়সী শিশুরা ইট তৈরি করে	
	Children (<18) loading or unloading bricks	শিশুরা (<18) ইট লোড বা আনলোড করছে	
	Children (<18) doing other jobs (e.g. cleaning, cooking)	শিশুরা (<18) অন্যান্য কাজ করছে (যেমন পরিষ্কার করা, রান্না করা)	
	Children (<18) on the top of the brick kiln	শিশুরা (<18) ইটের ভাটার উপরেচ	
	Children (<18) on or near coal piles	শিশুরা (<18) কয়লার স্তূপের উপর বা কাছাকাছি	
	Children (<18) near machinery, such as coal crushers or soil mixers	শিশুরা (<18) যন্ত্রপাতির কাছাকাছি, যেমন কয়লা ক্রাশার বা মাটি মিক্সার	
	nothing found	উপরের কোনটি পায়নি	
O2	During the conduct of this survey, did you notice any surveillance by the owner, a manager, sardar, or other workers?<br>[0= None to 5 Yes some of the time]	এই জরিপ পরিচালনার সময়, আপনি কি মালিক, ম্যানেজার, সর্দার বা অন্যান্য কর্মীদের দ্বারা কোন নজরদারি লক্ষ্য করেছেন?<br>[0= কেউ না থেকে 5=হ্যাঁ কিছু সময়]	

O3	Please note any other relevant observations here	এখানে অন্য কোন প্রাসঙ্গিক পর্যবেক্ষণ থাকলে নোট করুনঃ	
C1	Why was consent not granted?	সম্মতি কেন প্রদান করেনি?	If consent=0, thenanswer C1-C4
	1= Busy	1 = ব্যস্ত	
	2= Unwilling to participate	2 = অংশ নিতে অনিচ্ছুক	
	3= Told not to participate	3 = অংশ নিতে না বলা	
	4= Unknown reason	4 = অজানা কারণ	
	77= Other (Specify)	77 = অন্যান্য (নির্দিষ্ট করুন)	
C2	Which worker category are they from?<br>1= moulders<br>2= brick loaders<br>3= fireman<br>4= brick unloaders<br>5= Coal crusher	আপনি নিচের কোন দলের সাথে কাজ করেন?<br>1 = কাটামিস্ত্রি দল<br>2 = পোড়াইমিস্ত্রি দল<br>3 = ইট বোঝাই দল<br>4 = খালাসি / ইট আনলোডিং দল<br>5 = কয়লা ফাইনকারি	
C3	What's their age (years)?	বয়স কত (বছরে)	
C4	What's their sex?<br>1= Male<br>2= Female	লিঙ্গ<br>1=পুরুষ;<br>2=মহিলা	

SI References

Acemoglu, Daron and Alexander Wolitzky (2011). "The Economics of Labor Coercion." *Econometrica* 79.2, pp. 555–600. DOI: https://doi.org/10.3982/ECTA8963. eprint: https://onlinelibrary.wiley.com/doi/pdf/10.3982/ECTA8963. URL: https://onlinelibrary.wiley.com/doi/abs/10.3982/ECTA8963.

Adhvaryu, Achyuta, Namrata Kala, and Anant Nyshadham (2020). "The Light and the Heat: Productivity Co-Benefits of Energy-Saving Technology." *Review of Economics and Statistics* 102.4, pp. 779–792.

Ashraf, Quamrul H et al. (Apr. 2024). "Structural Change, Elite Capitalism, and the Emergence of Labour Emancipation." *The Review of Economic Studies* 92.2, pp. 808–836. ISSN: 0034-6527. DOI: 10.1093/restud/rdae043. eprint: https://academic.oup.com/restud/article-pdf/92/2/808/57334102/rdae043.pdf. URL: https://doi.org/10.1093/restud/rdae043.

Bangladesh Bureau of Statistics (2023). *Bangladesh Labour Force Survey 2022*. Survey Report. Dhaka, Bangladesh: Bangladesh Bureau of Statistics.

Benjamini, Yoav, Abba M. Krieger, and Daniel Yekutieli (Sept. 2006). "Adaptive linear step-up procedures that control the false discovery rate." *Biometrika* 93.3, pp. 491–507. ISSN: 0006-3444. DOI: 10.1093/biomet/93.3.491. eprint: https://academic.oup.com/biomet/article-pdf/93/3/491/1080958/933491.pdf. URL: https://doi.org/10.1093/biomet/93.3.491.

Boittin, Margaret L. et al. (2025). "Growing Awareness to Reduce Labor Abuse: An Experimental Test of a Migrant Domestic Workers' Rights-Awareness Campaign." *Journal of Law and Economics*. forthcoming.

Brooks, Nina et al. (2024). "Building Blocks of Change: The Energy, Health, and Climate Co-Benefits of More Efficient Brickmaking in Bangladesh." *Energy Research & Social Science* 117, p. 103738.

Brooks, Nina et al. (2025). "Reducing Emissions and Air Pollution from the Informal Brick Sector: Evidence from a Randomized Controlled Trial in Bangladesh." *Science* 388 (eadr7394). DOI: 10.1126/science.adr7394.

Das, Sajan et al. (2017). "Socioeconomic Conditions and Health Hazards of Brick Field Workers: A Case Study of Mymensingh Brick Industrial Area of Bangladesh." *Journal of Public Health and Epidemiology* 9.7, pp. 198–205.

Domar, Evsey D. (1970). "The Causes of Slavery or Serfdom: A Hypothesis." *The Journal of Economic History* 30.1, pp. 18–32. ISSN: 00220507, 14716372. URL: http://www.jstor.org/stable/2116721 (visited on 05/19/2025).

Eil, Andrew et al. (2020). *Dirty Stacks, High Stakes: An Overview of the Brick Sector in South Asia*. World Bank Publications - Reports 33727. World Bank.

Ercelawn, A. and M. Nauman (May 2004). "Unfree Labour in South Asia: Debt Bondage at Brick Kilns in Pakistan." *Economic and Political Weekly* 39.22, pp. 2235–2242.

Guérin, Isabelle et al. (2007). "Labour in Brick Kilns: A Case Study in Chennai." *Economic and Political Weekly* 42 (No. 7), pp. 599–606.

Gupta, Jayoti (2003). "Informal Labour in Brick Kilns: Need for Regulation." *Economic and Political Weekly* 38.31, pp. 3282–3292. ISSN: 0012-9976. JSTOR: 4413859. (Visited on 01/19/2024).

Harrison, Ann and Jason Scorse (Mar. 2010). "Multinationals and Anti-sweatshop Activism." *American Economic Review* 100.1, pp. 247–73.

Hausman, Jerry (2001). "Mismeasured Variables in Econometric Analysis: Problems from the Right and Problems from the Left." *Journal of Economic Perspectives* 15.4, pp. 57–67.

Henderson, Savanna, Jeni Sorensen, and Valentina Farinelli (2024). *Human Trafficking Protection: Improving Interventions to Protect Survivors of Forced Labor and Human Trafficking*. Evidence Review. Innovations for Poverty Action, Human Trafficking Research Initiative. URL: https://poverty-action.org/human-trafficking-protection-improving-interventions-protect-survivors-forced-labor-and-human.

International Labour Organization (1973). *C138 - Minimum Age Convention, 1973 (No. 138)*. https://www.ilo.org/dyn/normlex/en/f?p=NORMLEXPUB:12100:0::NO::P12100_ILO_CODE:C138. Adopted 26 June 1973, Entry into force 19 June 1976.

— (1999). *C182 - Worst Forms of Child Labour Convention, 1999 (No. 182)*. https://www.ilo.org/dyn/normlex/en/f?p=NORMLEXPUB:12100:0::NO::P12100_ILO_CODE:C182. Adopted 17 June 1999, Entry into force 19 Nov 2000.

Kara, Siddharth (2014). *Bonded Labor: Tackling the System of Slavery in South Asia*. Columbia University Press.

Liu, Xiaojin et al. (2019). "Toward Improving Factory Working Conditions in Developing Countries: An Empirical Analysis of Bangladesh Ready-Made Garment Factories." *Manufacturing & Service Operations Management* 21.2, pp. 379–397.

Luken, Ralph and Rodney Stares (2005). "Small Business Responsibility in Developing Countries: A Threat or an Opportunity?" *Business Strategy and the Environment* 14.1, pp. 38–53.

Ministry of Labor and Employment (2010). *National Child Labour Elimination Policy 2010*. Government of the People's Republic of Bangladesh.

Naidu, Suresh and Noam Yuchtman (Feb. 2013). "Coercive Contract Enforcement: Law and the Labor Market in Nineteenth Century Industrial Britain." *American Economic Review* 103.1, pp. 107–44. DOI: 10.1257/aer.103.1.107. URL: https://www.aeaweb.org/articles?id=10.1257/aer.103.1.107.

Okech, David, Lydia Aletraris, and Katherine R. McLaughlin (2025). "Human Trafficking Prevalence Research: Lessons and Practical Guidance from the Prevalence Research Innovation Forum (PRIF)." *Journal of Human Trafficking* 11.4, pp. 511–528. DOI: 10.1080/23322705.2025.2567784. eprint: https://doi.org/10.1080/23322705.2025.2567784. URL: https://doi.org/10.1080/23322705.2025.2567784.

Okech, David, Lydia Aletraris, and Elyssa Schroeder (2020). *Human Trafficking Statistical Definitions: Prevalence Reduction Innovation Forum*. Tech. rep. University of Georgia African Programming and Research Initiative to End Slavery. DOI: 10.13140/RG.2.2.31986.12484. URL: https://doi.org/10.13140/RG.2.2.31986.12484.

Sharma, Anisha, Manisha Shah, and Beata Łuczywek (Sept. 2024). "Understanding the Impact of Low-Cost Loans on Forced Labor." 32912. DOI: 10.3386/w32912. URL: https://www.nber.org/papers/w32912.

Tjaden, Jasper and Felipe Alexander Dunsch (2021). "The effect of peer-to-peer risk information on potential migrants – Evidence from a randomized controlled trial in Senegal." *World Development* 145, p. 105488. ISSN: 0305-750X. DOI: https://doi.org/10.1016/j.worlddev.2021.105488. URL: https://www.sciencedirect.com/science/article/pii/S0305750X21001005.

United Nations (2000). *Protocol to prevent, suppress and punish trafficking in persons especially women and children, supplementing the United Nations Convention against Transnational Organized Crime*. General Assembly resolution 55/25. URL: https://www.ohchr.org/en/instruments-mechanisms/instruments/protocol-prevent-suppress-and-punish-trafficking-persons.

Verhoogen, Eric (May 2008). "Trade, Quality Upgrading, and Wage Inequality in the Mexican Manufacturing Sector." *The Quarterly Journal of Economics* 123.2, pp. 489–530.

Zimmerman, Cathy et al. (May 2021). "Human Trafficking: Results of a 5-Year Theory-Based Evaluation of Interventions to Prevent Trafficking of Women From South Asia." *Frontiers in Public Health* 9, p. 645059. DOI: 10.3389/fpubh.2021.645059.
